# COLUMBUS—An Efficient and General Program Package for Ground and Excited State Computations Including Spin–Orbit Couplings and Dynamics

**DOI:** 10.1021/acs.jpca.5c02047

**Published:** 2025-07-08

**Authors:** Felix Plasser, Hans Lischka, Ron Shepard, Péter G. Szalay, Russell M. Pitzer, Rodolpho L. R. Alves, Adelia J. A. Aquino, Jochen Autschbach, Mario Barbatti, Jhonatas R. Carvalho, Julio C. V. Chagas, Leticia González, Andreas Hansen, Bhumika Jayee, Miklos Kertesz, Francisco B. C. Machado, Spiridoula Matsika, Silmar A. do Monte, Saikat Mukherjee, Dana Nachtigallová, Reed Nieman, Vytor P. Oliveira, Markus Oppel, Carol A. Parish, Jiri Pittner, Luan G. F. dos Santos, Armin Scrinzi, Mahesh K. Sit, Rene F. K. Spada, Mushir Thodika, Daniel C. A. Valente, Álvaro Vázquez-Mayagoitia, Elizete Ventura, Julia Westermayr, Aleksandr Zaichenko, Zhiyong Zhang

**Affiliations:** † Department of Chemistry, 152008 Loughborough University, Loughborough LE11 3TU, United Kingdom; ‡ Department of Chemistry and Biochemistry, 6177 Texas Tech University, Lubbock, Texas 79409, United States; § Chemical Sciences and Engineering Division, 1291 Argonne National Laboratory, Lemont, Illinois 60439, United States; ∥ Laboratoy of Theoretical Chemistry, Institute of Chemistry, ELTE Eötvös Loránd University, H-1117 Budapest, Hungary; ⊥ Department of Chemistry and Biochemistry, 2647 The Ohio State University, Columbus, Ohio 43210, United States; # Departamento de Química, CCEN, 28097 Universidade Federal da Paraíba, 58059-900 João Pessoa, Brazil; ∇ Department of Mechanical Engineering, Texas Tech University, Lubbock, Texas 79409, United States; ○ Department of Chemistry, University at Buffalo, State University of New York, Buffalo, New York 14260-3000, United States; ◆ 128791 Aix Marseille University, CNRS, ICR, 13397 Marseille, France; ¶ Department of Chemistry, Aeronautics Institute of Technology, 12228-900 São José dos Campos, Brazil; †† Advanced Scientific Computing and Modeling Laboratory, Aeronautics Institute of Technology, 12228-900 São José dos Campos, Brazil; ‡‡ Institute of Theoretical Chemistry, Faculty of Chemistry, University of Vienna, 1090 Vienna, Austria; §§ Vienna Research Platform on Accelerating Photoreaction Discovery, University of Vienna, 1090 Vienna, Austria; ∥∥ Mulliken Center for Theoretical Chemistry, 9374 University of Bonn, 53115 Bonn, Germany; ⊥⊥ Physical Sciences and Computational Division, 6865 Pacific Northwest National Laboratory, Richland, Washington 99354, United States; ## Chemistry Department and Institute of Soft Matter, 8368 Georgetown University, Washington, District of Columbia 20057-1227, United States; ∇∇ Department of Chemistry, 6558 Temple University, 1901 N 13th St, Philadelphia, Pennsylvania 19122, United States; ○○ Faculty of Chemistry, Nicolaus Copernicus University in Torun, 87100 Torun, Poland; ◆◆ Institute of Organic Chemistry and Biochemistry, Academy of Sciences of the Czech Republic, CZ-16610 Prague, Czech Republic; ¶¶ IT4Innovations, VŠBTechnical University of Ostrava, 708 00 Ostrava, Czech Republic; ††† Department of Chemistry, Gottwald Center for the Sciences, 6888 University of Richmond, Richmond, Virginia 23173, United States; ‡‡‡ J. Heyrovsky Institute of Physical Chemistry, Academy of Sciences of the Czech Republic, v.v.i., Dolejškova 3, 18223 Praha 8, Czech Republic; §§§ Department of Physics, Ludwig Maximilian University, Theresienstrasse 37, 80333 Munich, Germany; ∥∥∥ Departamento de Física, 74360 Instituto Tecnológico de Aeronáutica, 12228-900 São José dos Campos, São Paulo, Brazil; ⊥⊥⊥ National Renewable Energy Laboratory, 15013 Denver West Parkway, Golden, Colorado 80401, United States; ### Computational Science Division, Argonne National Laboratory, 9700 South Cass Avenue, Lemont, Illinois 60439, United States; ∇∇∇ Wilhelm-Ostwald-Institute for Physical and Theoretical Chemistry, Leipzig University, 04103 Leipzig, Germany; ○○○ ScaDS.AI (Center for Scalable Data Analytics and Artificial Intelligence) Dresden/Leipzig, 04105 Leipzig, Germany; ◆◆◆ Stanford Research Computing, Stanford University, Stanford, California 94305, United States; ¶¶¶ Institut Universitaire de France, 75231 Paris, France; α Department of Chemistry, Northwestern University, Evanston, Illinois 60208, United States

## Abstract

The COLUMBUS program system provides the tools for performing high-level multireference (MR) computations, including the multireference configuration interaction (MRCI) method and its multireference averaged quadratic coupled cluster (MR-AQCC) extension, allowing computations on a wide range of fascinating atomic and molecular systems, including the treatment of open-shells and complicated excited state phenomena. The inclusion of spin–orbit coupling (SOC) directly within the MRCI step enables the description of systems containing heavy elements, such as lanthanides and actinides, whose properties are strongly influenced by SOC. Analytic energy gradients and nonadiabatic couplings at the correlated MRCI level provide the foundation for a variety of dynamics studies, giving insight into ultrafast photochemistry. New and ongoing method developments in COLUMBUS include the computation of spin densities, improved descriptions of ionic states, enhancements to the AQCC method, and the porting of COLUMBUS to graphical processing units (GPUs). New external interfaces enable an enhanced description of electronic resonances and molecules in strong laser fields. This work highlights these new developments while providing a detailed account of the diverse applications of COLUMBUS in recent years.

## Graphical abstract

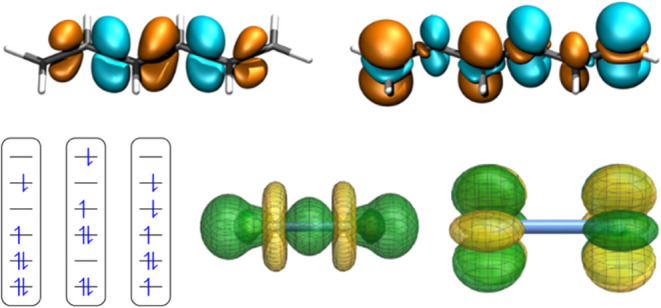

## 1 Introduction

Within the last decades, quantum chemistry has become an indispensable component of molecular science investigations. Whereas ground-state reactivity can often be described using standard density functional theory (DFT) computations, there are significant and vital classes of problems where DFT fails, most notably in cases involving strongly correlated electrons, distorted structures, broken bonds, and doubly excited states.^1,2^ In such cases, multireference (MR) methods provide an attractive option by allowing the treatment of all relevant electronic configurations of a given molecular system on an equal footing. Aside from accurate energies, it is often also essential to be able to compute other electronic properties, including energy gradients, allowing one to determine molecular geometries and their dynamic evolution, transition dipole moments for optical transition strengths, as well as nonadiabatic (NAC) and spin–orbit coupling (SOC) matrix elements to determine interstate transition probabilities.

In the spirit of the above discussion, the purpose of COLUMBUS is to provide a platform for flexible and high-level multireference computations along with the functionality to compute the relevant properties and interstate coupling terms. Within the landscape of multireference programs, COLUMBUS stands out through its flexible multiconfigurational self-consistent field (MCSCF) module and its flexible, powerful, and well-parallelized multireference configuration interaction (MRCI) module. The latter includes an extension for variational spin–orbit configuration interaction and various extensivity corrections. Furthermore, COLUMBUS provides energy gradients, nonadiabatic couplings, spin–orbit couplings, and other electronic properties at the MRCI and MCSCF levels.

The purpose of this paper is to present the computational capabilities of COLUMBUS, including ongoing developments, and to illustrate them through a range of recent applications. We start with a brief overview of the program structure and main capabilities of Columbus (Section 2). Subsequently, Section 3 introduces recent advances, including the computation of MCSCF spin densities within the originally spin-free graphical unitary group approach (GUGA), new treatments of ionic states as defined in valence-bond theory—long recognized as a challenge^3–5^ for many multireference frameworks—and a detailed account of the averaged quadratic coupled cluster (AQCC) approach within a linear response formalism (AQCC-LRT). This section also covers current efforts to port COLUMBUS to GPUs and highlights two new external interfaces, used for computing electronic resonances and modeling attosecond experiments, respectively. Section 4 presents selected case studies where multireference methods are essential, including calibration of the semiempirical fractional occupation number weighted electron density (FOD)^6^ method against multireference unpaired densities and applications to graphene defects, phenalenyl oligomers, polyradicals, and ionic hydrocarbons. We conclude this part with the intricate case of a uranium–uranium bond, where both multireference and SOC effects are significant. Section 5 focuses on excited-state treatments, beginning with the analysis of covalent and ionic character and continuing with studies on doped periacenes and the triatomic CUO molecule, again emphasizing spin–orbit coupling effects. Finally, Section 6 demonstrates the use of COLUMBUS in various photodynamics simulations, including recent developments that leverage trajectory data for machine learning applications.

## 2 Overview

A summary of the COLUMBUS program structure is given in Figure 1. We describe these features here and refer to the various sections that exemplify their use. At the heart of COLUMBUS are its MCSCF and MRCI modules. Both are based on the GUGA formalism,^7^ which enables a flexible definition of various wave function types and an efficient computation of the associated matrix elements. These methods enable the accurate treatment of challenging electronic structures, such as those found in graphene defects (Section 4.2), polyradicals (Section 4.3), and nonstandard bonding situations (Section 4.5). In MCSCF and MRCI computations, COLUMBUS allows for complete active space SCF (CASSCF) and restricted active space SCF (RASSCF), as well as more specialized and highly customized active space definitions (one such example is described in Section 6.1). COLUMBUS utilizes an uncontracted MRCI with singles and doubles (MR-CISD) approach^8,9^ implemented using a direct configuration interaction (CI) algorithm, which avoids the explicit calculation of the Hamiltonian matrix elements. Combined with an efficient parallelization algorithm, CI dimensions of several billion can be handled routinely on standard computer clusters, making many interesting calculations possible,^10–12^ as illustrated in various examples below. Further improvement, aimed at utilizing GPUs, is currently ongoing (Section 3.4).

**Figure 1:**
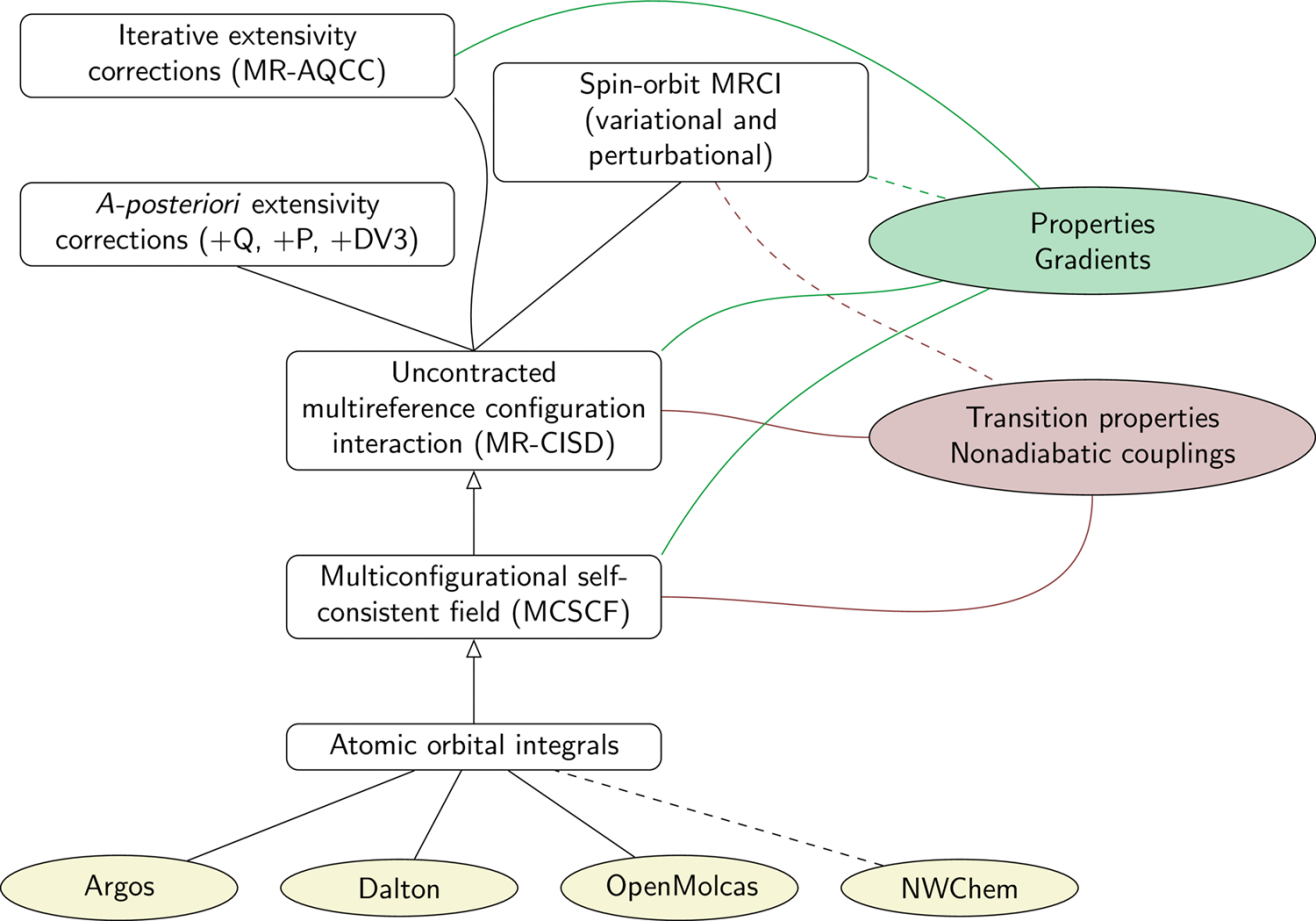
Summary of the functionality provided within COLUMBUS. Features in the release version are marked with solid lines, while features under development are marked with dashed lines.

A significant advantage of uncontracted MRCI is that, due to its variational nature, it is relatively straightforward to compute electronic (transition) properties, gradients, and nonadiabatic couplings (shown to the right in Figure 1), all of which are implemented for MCSCF and MRCI. The availability of analytic gradients allows for structure optimizations (illustrated in various sections below), and the combination with nonadiabatic couplings provides the basis for photodynamics simulations (Section 6). Along with the wave function properties, COLUMBUS naturally produces natural orbitals, densities, transition densities, and so on for the correlated wave functions. Recently, we have also demonstrated how the originally spin-free GUGA formalism can nonetheless be utilized to compute spin-densities (Section 3.1).

COLUMBUS features functionality to treat relativistic spin–orbit coupling (SOC) effects within its SO–CI module, also based on the GUGA formalism.^13^ SO–CI can be performed in a variational fashion, meaning that SOC, static, and dynamic correlation are treated in one combined step, providing a high-level description even of challenging electronic structures. Using SO–CI becomes particularly important for heavy elements with unpaired electrons, and its applications are illustrated below in the case of bonding between two uranium atoms (Section 4.6) and the CUO triatomic molecule (Section 5.3). The computation of gradients and nonadiabatic couplings from SO–CI is available in a development version but has not been officially released at the time of writing. Alternatively, a perturbative version of the SO–CI module is available,^14^ where SOC is computed between standard spin-free MRCI wave functions. This implementation is particularly well-suited to dynamics simulations, as presented in Section 6.2.

CI methods are advantageous due to their variational nature, and this is reflected in numerically stable results as well as the relatively straightforward implementation of gradients, nonadiabatic couplings, and other wave function properties. Conversely, a difficulty when applying CI is its lack of extensivity, meaning that electron correlation in larger molecules is not as well described as in smaller molecules. Practically, one finds that MRCI is generally reliable only if an extensivity correction is used and COLUMBUS provides various possible options (Figure 1, upper left). As one conceptually simple and computationally cheap option, *a posteriori* corrections can be used.^15,16^ These are often generically denoted as (+Q). Within COLUMBUS, different variations are implemented, and we generally recommend^17^ using the Pople (+P)^18^ or Davidson-Silver (+DV3)^19^ corrections. *A posteriori* corrections tend to provide reliable energies but it is not possible to use them to compute energy gradients. For this reason, COLUMBUS also provides iterative extensivity corrections, that is, methods where already the CI procedure is modified. The most prominent one of these is the MR-averaged quadratic coupled cluster (MR-AQCC) method.^20^ MR-AQCC provides reliable energies of the ground and excited states along with their gradients. The MR-AQCC method and its linear response theory (LRT) variant are discussed in Section 3.3. Furthermore, the practical influence of extensivity corrections in producing accurate excitation energies is discussed in detail in Sections 3.2 and 5.1 within a valence-bond framework focusing on the ionic and covalent character of the state. Finally, noting that MR-AQCC gives access to extensivity-corrected density matrices, we use it in Section 4.1 to provide an accurate reference for calibrating an approximate method for describing unpaired electrons.

One crucial structural feature of COLUMBUS is the modular concept, which organizes different computational steps, including atomic orbital (AO) integrals, GUGA and wave function setup, integral transformation, CI steps, and analytic energy gradient and nonadiabatic coupling calculations. This approach enables the combination of COLUMBUS with other quantum chemical program packages to share atomic orbital (AO) integrals. In addition to the proprietary Argos program, well-established interfaces to DALTON^21^ and OpenMolcas^22^ are available, along with a more experimental interface to NWChem^23^ (see Figure 1, bottom). Furthermore, the SCF, MCSCF, and geometry optimization modules of OpenMolcas can be used in combination with the MRCI program module of COLUMBUS, providing enhanced flexibility for setting up various jobs.^24^ The modular setup also provides an ideal basis for external interfaces. Below, we illustrate this in the case of complex absorbing potentials used for electronic resonances (Section 3.5) and hybrid antisymmetrized coupled channels for attosecond spectroscopy (Section 3.6).

COLUMBUS is developed as an open-source project via a public repository^25^ and is released under the GNU Lesser General Public License. Releases are distributed from the same web page^25^ in the form of source code and precompiled executables. COLUMBUS is primarily developed for Linux systems. It has been installed and used on a variety of high-performance cluster architectures.

## 3 Method Developments

### 3.1 Spin Densities at the MCSCF Level

Recently, the calculation of spin densities was implemented in COLUMBUS for wave functions obtained via MCSCF calculations.^26^ Given a wave function |ψ;*S*,*M*⟩, which is an eigenfunction of the operator *Ŝ* ^2^, that is

1 $${Ŝ}^{2}|\psi ;S,M\rangle =S\left(S+1\right)|\psi ;S,M\rangle$$

with eigenvalues *S* ≥ 0 and of the operator *Ŝ*_*z*_

2 $${Ŝ}_{z}\left|\psi ;S,M\right\rangle =M\left|\psi ;S,M\right\rangle$$

determining the projection along the *z*-axis with quantum numbers *M* = −*S*, −*S* + 1,···,*S* – 1,*S*, the calculation of spin-density matrix elements is given by

3 $$D_{\mathrm{qp}}^{\left(1,0,M\right)}=\left\langle \psi ;S,M\left|a_{p\alpha }^{†}a_{q\alpha }-a_{p\beta }^{†}a_{q\beta }\right|\psi ;S,M\right\rangle$$

In COLUMBUS, this calculation is implemented using the Graphical Unitary Group Approach (GUGA),^27^ as this procedure avoids transforming the wave function from a configuration state function (CSFs) basis to a determinantal basis to carry out the difference of the α and β blocks. Note that this last procedure would be highly inefficient or unfeasible for large CSF expansions since the determinantal expansion grows faster than the CSF one, especially for larger numbers of open-shell electrons.

The current implementation calculates the spin-density matrix elements for *M* = *S* using^26,28^

4 $$D_{\mathrm{qp}}^{\left(1,0,S\right)}=\frac{\left(2-\frac{1}{2}N\right)}{\left(S+1\right)}D_{\mathrm{qp}}^{\left(0\right)}-\frac{1}{\left(S+1\right)}\sum_{k}d_{\mathrm{qkkp}}$$

in which *N* is the total number of electrons, *D*_*qp*_^(0)^ is an element from the one-particle reduced density matrix (1-RDM) and *d*_*qkkp*_ = ⟨ψ|*ê*_*pkkq*_|ψ⟩ are the unsymmetrized two-particle reduced density matrix (2-RDM) elements. Both the 1-RDM and 2-RDM are available within the GUGA formalism. Note that the calculation of the last term on the right-hand side of eq 4 would require a sum over elements of a four-dimensional array. However, a more efficient approach was implemented to compute the spin-density matrix alongside the 1-RDM and 2-RDM to reduce the additional computational effort to obtain the elements *D*_*qp*_^(1,0,*S*)^.

The analysis of the spin density is useful, for example, for the study of organic radicals. In particular, diradicals have applications in various areas of optoelectronics and spintronics.^29–33^ In this work, we employed the implementation to calculate the spin density for the diradical *para*-quinodimethane. Investigations into the electronic structure of this system can be traced to the works by Szwarc^34^ and Coulson et al.^35^ Recent work by Chagas et al. has also explored the electronic effects of the CH_2_ groups on the benzenoid ring.^36^ A detailed study of the electronic structure of this molecule was recently carried out by Matasović et al.^37^ to investigate the conversion from a closed-shell character attained with the CH_2_ groups coplanar to the ring structure to an open-shell character with the CH_2_ groups orthogonal. Here, we utilized the planar geometry from that work, optimized at the PBE/ANO-S-VDZP level, and two additional structures with different values for the θ angle between the ring plane and the CH_2_ group (Figure 2a): 0, 50, and 90°.

**Figure 2:**
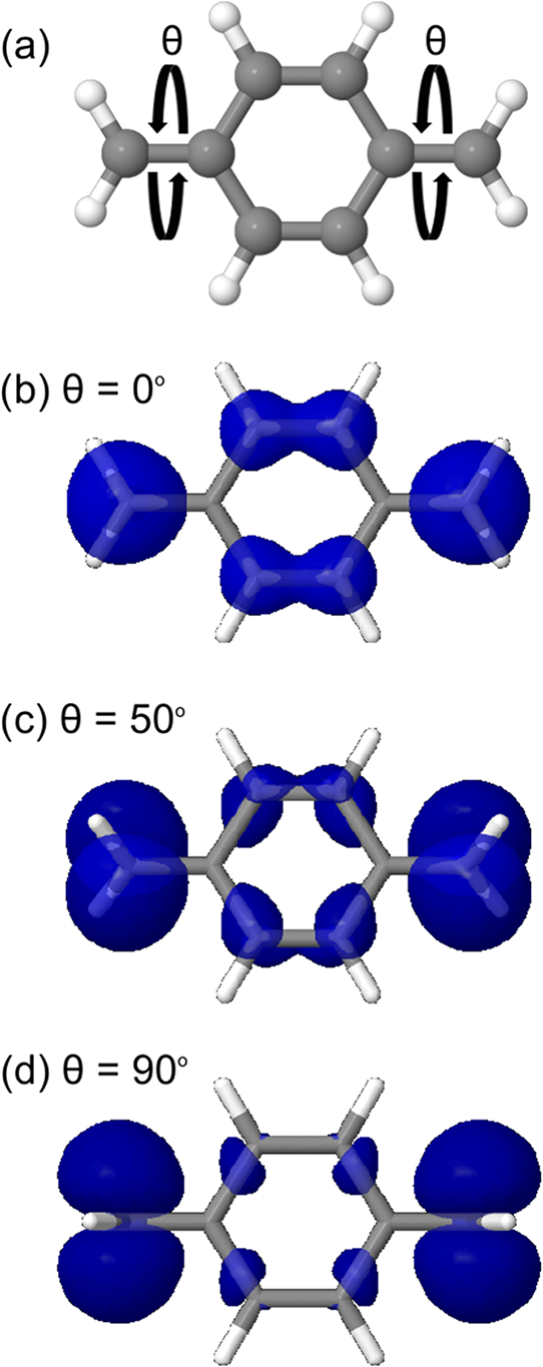
*para*-Quinodimethane molecule with panel (a) highlighting the angle (θ) between the ring plane and the CH_2_ group. Panels (b–d) display the spin densities for the T_1_ state of the studied structures with different θ angles. The employed isovalue is equal to 0.003 eÅ^–3^.

Next, we carried out calculations using the SA-CASSCF method, considering 8 electrons in 8 orbitals (CAS­(8,8)) and the cc-pVDZ basis set for the lowest triplet state (T_1_). The spin densities for the three structures are presented in Figure 2.

From the inspection of Figure 2, the decrease in the spin density of the ring structure is noticeable with the increase in the angle θ. This highlights the loss in delocalization upon twisting and illustrates that the spin density is increasingly localized on the radical centers.

### 3.2 Diagnostics and Scaling Procedures for Ionic States

Whereas MCSCF presents itself as a compelling method for providing qualitatively correct starting points in various situations, including open shells and highly excited states, one critical area that poses notorious challenges is the description of ionic ππ* states.^3–5^ These states can be best understood in a valence-bond framework.^38,39^ To summarize, within the standard MO picture, the singlet and triplet highest-occupied molecular orbital/lowest-unoccupied molecular orbital (HOMO/LUMO) excited states are given as

5 $$\Psi_{s}=|h\mathrm{l̅}\rangle +|l\mathrm{h̅}\rangle$$

6 $$\Psi_{T}=|h\mathrm{l̅}\rangle -|l\mathrm{h̅}\rangle$$

where *h* and *l* refer to the HOMO and LUMO, and the bar signifies β-spin. Using a valence-bond-type description and expressing these equations in terms of localized orbitals, denoted *a* and *b*, yields^17,39^

7 $$\Psi_{s}=|a\mathrm{a̅}\rangle -|b\mathrm{b̅}\rangle$$

8 $$\Psi_{T}=|b\mathrm{a̅}\rangle -|a\mathrm{b̅}\rangle$$

Crucially, the singlet consists of configurations in which both electrons occupy either orbital *a* or orbital *b*, and the state is therefore classified as ionic. The opposite is true for the triplet, which is classified as a diradical. Practically speaking, the ionic nature of the singlet requires enhanced electron correlation in its description, often posing severe challenges to the MCSCF method. There are two interrelated problems: how do we diagnose issues related to ionic states, and how do we fix these problems? Both have been addressed recently, as discussed below.

A new diagnostic for ionic states, denoted *Q*_*a*_^*t*^, has been developed in ref 17. *Q*_*a*_^*t*^ is based on a population analysis of the transition density, carried out with TheoDORE,^40^ to postprocess the COLUMBUS computation. Before proceeding, we comment on the range of values accessible by *Q*_*a*_^*t*^. It is per construction always greater than zero. However, there is no obvious upper limit. Whereas *Q*_*a*_^*t*^ values of singlet states are usually found to be below one, one finds values close to two for some triplet states.^17^

Figure 3 presents an analysis of computed errors in excitation energies (using the theoretical best estimates from ref 41 as reference) for a test set of 11 π-conjugated molecules plotted against the *Q*_*a*_^*t*^ diagnostic. Starting with the SA-CASSCF level (Figure 3a) and considering the ππ* states (red circles), we find that the diagnostic performs precisely as intended. For the covalent ππ* states seen at low *Q*_*a*_^*t*^ values (below 0.2), we find good accuracy, and all errors are below 0.5 eV. Conversely, for the ionic ππ* states seen at higher *Q*_*a*_^*t*^ values (above 0.3), significant errors of 1–2 eV are observed. Finally, the nπ* states (green squares) yield intermediate errors. Figure 3a thus highlights the power of the *Q*_*a*_^*t*^ diagnostic for ππ* states, providing a clear differentiation between cases where the SA-CASSCF is expected to be reliable and where it is not.

**Figure 3:**
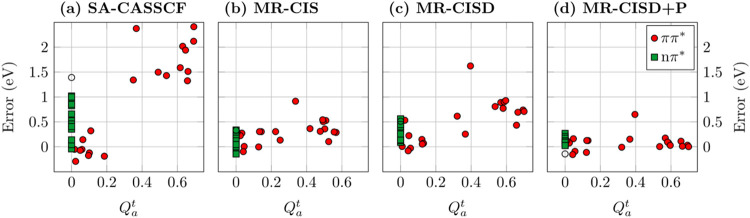
Errors of computed vertical singlet excitation energies for a test set of 11 molecules plotted against the *Q*_*a*_^*t*^ diagnostic measuring ionic character using (a) SA-CASSCF, (b) MR-CIS, (c) MR-CISD, and (d) MR-CISD+P, all using the aug-cc-pVDZ basis set. States are grouped according to type: ππ* (circles) and *n*π* (squares) states; the out-of-plane ππ* state of cyanoformaldehyde is shown as an empty circle. Adapted from ref 17. Copyright 2023 American Chemical Society.

Progressing along the hierarchy of MRCI methods, as implemented in COLUMBUS, generally improves the results from CASSCF, and this is shown in Figure 3b–d. However, an important point to note is that MR-CIS sometimes yields improved results compared to MR-CISD, and this discrepancy can be attributed to the lack of size extensivity. But, using finally an extensivity-corrected version (MR-CISD+P) yields excellent results for all types of states.

While the above work demonstrates that ionic states can be effectively detected, correcting their energies at the CASSCF stage would be beneficial. Work in this direction is currently in progress. The formalism starts by realizing that the energies of the singlet and triplet states in eqs 5 and 6 differ by twice the exchange integral *g*_*hl,hl*_ where the triplet is stabilized and the singlet is raised in energy.^42,43^ Furthermore, it is worth noting that σ-correlation can be understood to act by screening this exchange interaction.^17,43,44^ The idea behind the new procedure is to scale the involved terms to mimic screening via the σ-electrons. Practically, we can define the diagonal shift Hamiltonian

9 $${Ĥ}_{\mathrm{shift}}=\left(1-\mu \right)\sum_{p<q}{\mathrm{P̂}}_{\mathrm{pq}}g_{\mathrm{pq},\mathrm{pq}}$$

where *P̂*_*pq*_ is the projector onto all CSFs where orbitals *p* and *q* are singly occupied and singlet spin-coupled, and all other orbitals are either unoccupied or doubly occupied. The value of μ is left as an adjustable parameter. A scaled MCSCF version can now be defined by subtracting this shift Hamiltonian from the usual MCSCF Hamiltonian. Preliminary results suggest that this procedure specifically targets singlet ππ* states, leaving the ground state and other excited states largely unaffected.

The proposed procedure directly targets the underlying two-electron integrals and is expected to allow for a much more fine-grained tuning than global energy scaling procedures.^45,46^ Crucially, as opposed to multiconfigurational pair density functional theory,^47^ the shift is included self-consistently in the MCSCF procedure, meaning that it also affects orbital optimization and ultimately enables the computation of energy gradients and nonadiabatic couplings. Work on this is currently in progress, and the developed method will be presented in a separate communication.

### 3.3 AQCC-LRT as a Viable Alternative to MR-CISD Calculations of Excited States

Multireference Averaged Coupled Pair Functional (MR-ACPF)^48^ and MR-AQCC^20,49^ methods are powerful tools for correcting the size-extensivity error of MR-CISD. They have been utilized in numerous applications (for a review, see refs 50,51 ). Their main advantage over the alternative, Davidson-type corrections, is that a density is associated with the corrected energy,^48^ and therefore, gradient and other properties can be calculated.^52,53^

In the MR-AQCC method,^20,49^ a functional of the correlation energy (Δ*E*_α_) of state α

10 $$F_{\alpha }\left({\mathrm{c̲}}^{\alpha }\right)=\frac{\left\langle \sum_{i}c_{i}^{\alpha }\Phi_{i}\left|Ĥ-E_{0}^{\alpha }\right|\sum_{i}c_{i}^{\alpha }\Phi_{i}\right\rangle }{\sum_{i\in \mathrm{int}}{\left(c_{i}^{\alpha }\right)}^{2}+G\sum_{i\notin \mathrm{int}}{\left(c_{i}^{\alpha }\right)}^{2}}$$

is optimized to get the coefficients (*c*_*i*_^α^) corresponding to the expansion functions Φ_*i*_. Here *E*_0_^α^ is the reference energy of the state considered, and the two summations in the denominator run for the internal and all other excitations, respectively. By choosing the factor *G* appropriately, various methods can be defined. Trivially, *G* = 1 gives the MR-CISD functional. For MR-AQCC, we have $G=1-\frac{\left(n_{e}-3\right)\left(n_{e}-2\right)}{n_{e}\left(n_{e}-1\right)}$ , while for MR-ACPF $G=\frac{2}{n_{e}}$ with *n*_*e*_ being the number of (correlated) electrons.

The stationary equations of this functional can be cast into a matrix eigenvalue problem^54,55^

$$\left\langle \Phi_{j}\left|\left(Ĥ-E_{0}^{\alpha }+{\mathrm{\Delta ̂}}_{\alpha }\right)\right|\underset{i}{\sum }c_{i}^{\alpha }\Phi_{i}\right\rangle =\Delta E_{\alpha }c_{j}^{\alpha }$$ 11

with the diagonal shift being

12 $$\hat{\Delta_{\alpha }}=\sum_{k\notin \mathrm{int}}\left(1-G\right)\Delta E_{\alpha }\left|\Phi_{k}\right\rangle \left\langle \Phi_{k}\right|$$

Note that for CI, the shift is zero. Thus, the formalism reproduces the usual CI eigenvalue equations. On the other hand, for *G* ≠ 1, the shift is state-dependent since it includes the correlation energy of the given state. In this case, the eigenvalues (Δ*E*_α_) and the coefficient vectors (*c* ^α^) belonging to different states are eigenpairs of different Hamiltonians. Whereas the resulting nonorthogonality of the eigenvectors is not a problem for the calculation of single-state properties,^48^ it does not allow for the computation of transition properties.

The MR-AQCC method has been successfully applied to numerous ground-state applications, such as describing the biradical character and singlet–triplet splitting in polycyclic aromatic hydrocarbons (PAHs)^11,56,57^ as well as the study of bonding in the chromium dimer.^10^ It can also be applied to excited states^58^ without symmetry or spin restriction. This is achieved in COLUMBUS by a root-following technique, where the subspace root with the maximal overlap with a selected reference root is updated during the Davidson subspace approach.^55^ This procedure works well when the desired roots are well-separated and possess distinct characteristics. However, in practice, it lacks the stability of MR-CISD calculations, and intruders often hinder convergence.

We adopted a modified version of the AQCC method, which had been previously developed,^54^ and removes the disadvantage of AQCC associated with the state-dependent diagonal shift. However, it still has the advantage of including size-extensivity corrections intrinsically. As shown in ref 54, the state dependence of the diagonal shift can be avoided by an alternative derivation of the excited state equations using linear response theory (LRT).^59^ Starting from the AQCC ground state solution, for the excited states, the eigenvalue equations of the response matrix^59^ should be considered, i.e., the following equations need to be solved

13 $$\left\langle \Phi_{j}\left|\left(Ĥ-E_{0}^{\alpha }+\hat{\Delta_{0}}\right)\right|\sum_{i}c_{i}^{\alpha }\Phi_{i}\right\rangle =\Delta E_{\alpha }c_{j}^{\alpha }$$

for more details on the derivation of the method called AQCC-LRT, see ref 54 here, we draw attention only to the fact that for all excited states, the shift depends on the correlation energy of the ground state (Δ*E*_0_), i.e., energies and coefficients of all excited states are obtained as the eigenpairs of the same matrix. One can, therefore, calculate excited states just like from CI and obtain several excited states at the same time without the necessity of using root following.

AQCC and AQCC-LRT would give the same results if the reference energies for the ground and excited states were of the same quality, resulting in (nearly) identical correlation energy. Although this is often not the case, ref 54 shows that the excitation energies are usually very similar to the two AQCC procedures.

The other advantage of AQCC-LRT is that using the LRT technology,^59^ it is possible to define the transition density between state 0 and α as^54^

14 Γt0α=a0Γt0αCI+(1−a0)Γt0αref

with

15 $$a_{0}=\frac{\sum_{i}{\left(c_{i}^{0}\right)}^{2}}{\sum_{i\in \mathrm{int}}{\left(c_{i}^{0}\right)}^{2}+G\sum_{i\notin \mathrm{int}}{\left(c_{i}^{0}\right)}^{2}}$$

where ^CI^Γ_*t*_^0α^ and ^ref^Γ_*t*_^0α^ are the CISD and reference transition densities, respectively.

Compared with standard MR-AQCC calculations, below, we demonstrate the usefulness of MR-AQCC-LRT by studying all-*trans* hexatriene where the different character of the lowest excited states poses a particular challenge. The condition for the MR-AQCC and MR-AQCC-LRT to yield similar results is not generally fulfilled, as the description of the ionic state in the reference space is significantly worse; thus, the correlation energy is expected to be larger in magnitude than for the ground state. In addition, we demonstrate that the transition properties can also be calculated with the MR-AQCC-LRT method, allowing a clear characterization of the wave function.

The ground and the three lowest excited states (2^1^A_g_, 1^1^B_u_, and 2^1^B_u_) of the all-*trans* hexatriene are considered below. Reported results are from MR-CISD, MR-CISD with Pople’s approximate extensivity correction (MR-CISD+P),^18^ MR-AQCC, and MR-AQCC-LRT. The geometries and orbitals are taken from the calculations reported below (see Section 5.1). The reference space was always a full π-valence CAS­(6,6), and only the core orbitals were frozen. All the calculations used the cc-pVDZ basis set.^60^ For the analysis of the transition properties, the TheoDORE 3.2 program^40^ was used. We present the transition densities and the *Q*_*a*_^*t*^ diagnostic,^17^ as also discussed in the previous section. In this context it is worth noting that *Q*_*a*_^*t*^ values and transition dipole moments can only be computed using MR-CISD and MR-AQCC-LRT while this is not possible with MR-CISD+P or standard MR-AQCC.

Technically, after evaluating the ground state correlation energy (a single root AQCC calculation), the MR-AQCC-LRT procedure runs like a CI one: one obtains the eigenpair for all three excited states in one calculation, and there is no need for root following. No intruders appeared at least while obtaining three roots in both irreps.

In Table 1, the *Q*_*a*_^*t*^ values indicate that 2^1^A_g_ and 2^1^B_u_ states have covalent character, while the 1^1^B_u_ state is ionic. The excitation energies obtained with AQCC and MR-AQCC-LRT for the covalent 2^1^A_g_ and 2^1^B_u_ states agree within 0.01 eV since the correlation energies for the ground state and these excited states agree within 0.1 eV. In contrast, the discrepancy is larger for the ionic state, with the AQCC-LRT excitation energy exceeding AQCC and MR-CISD+P by 0.4 eV. The underlying reason is that this state has a significantly higher correlation energy—nearly 2 eV—due to the absence of σ-correlation in the reference space. As a result, the AQCC-LRT correction is smaller, leading to a higher predicted excitation energy. Note that a significant improvement over standard MR-CISD is still observed. Moreover, for the 2^1^B_u_ state the *Q*_*a*_^*t*^ value is reduced almost to half with MR-AQCC-LRT compared to MR-CISD, indicating reduced ionic character for this state. These results demonstrate that to avoid the unphysical mixing of covalent and ionic characters, a size-extensivity correction is essential in this case.

**Table 1:** Relative Energy (eV), Transition Dipole Moments (μ^*t*^, a.u.) and *Q*_*a*_^*t*^ Diagnostic for the Three Low-Lying Excited States of All-*trans* Hexatriene Obtained by the Different Methods

	MR-CISD			MR-CISD+P	MR-AQCC	MR-AQCC-LRT		
state	Δ*E*	μ*^t^*	*Q*_*a*_^*t*^	Δ*E*	Δ*E*	Δ*E*	μ^*t*^	*Q*_*a*_^*t*^
2^1^A_g_	5.816	0.000	0.088	5.755	5.743	5.755	0.000	0.077
1^1^B_u_	6.520	2.826	0.828	5.802	5.764	6.197	2.745	0.789
2^1^B_u_	7.005	0.129	0.131	6.915	6.880	6.902	0.145	0.071

The transition densities of the relevant states are presented in Figure 4. As discussed previously,^17,39^ the transition densities are located around the bonds for covalent states, whereas they are on the atoms for the ionic 1^1^B_u_ state. More specifically, we observed the signature of σ-correlation,^17,44^ as shown by additional transition density contributions near the atoms, which gives the individual atomic contributions the shape of d_*z* ^2^_ orbitals. Such σ-correlation contributions can be visualized only by methods such as MR-CISD and MR-AQCC-LRT, both of which explicitly incorporate σ-correlation in the wave function. The situation is different for second-order perturbation theory methods such as CASPT2. Such methods do provide an appropriate energy correction, but they usually do not give access to the perturbed transition densities that would be needed to visualize these σ-correlation contributions (and to provide appropriate perturbative corrections to computed transition properties).

**Figure 4:**
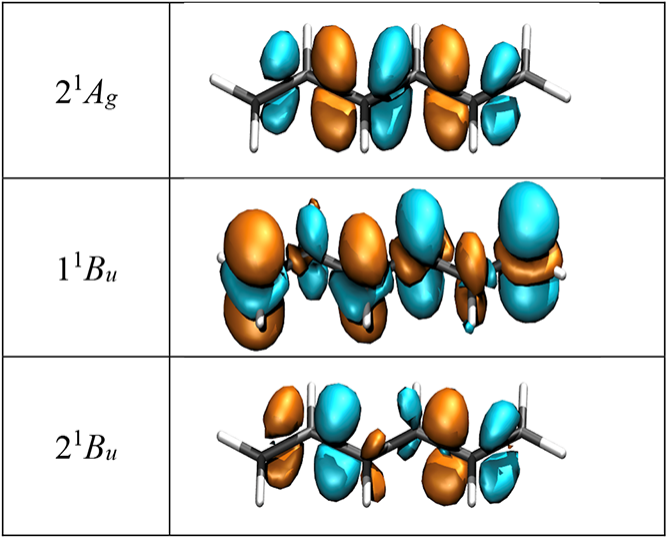
Transition densities (isovalue: 0.002 au) between the ground and first three excited states of hexatriene, computed with the MR-AQCC-LRT method.

The presented results on the intricate case of the three lowest excited states of all-*trans* hexatriene demonstrate that MR-AQCC-LRT is a viable tool for calculating transition properties, as the inclusion of the size-extensivity correction substantially improves the results compared to CI. Since the diagonalization of the same matrix yields the excited states, calculations are more straightforward than in the case of the original MR-AQCC. Additionally, analysis of the excited-state characters via transition densities is also possible. In the critical case of the ionic state, where the conditions for the LRT approach are not exactly met, it does not reproduce AQCC excitation energy precisely, but is still superior to MR-CISD.

### 3.4 A First Step into GPU Application in COLUMBUS

Graphical Processing Units (GPUs) have significantly transformed the use of quantum chemical calculations.^61^ GPUs originated from applications in consumer video gaming and transitioned into systematic scientific programming with the advent of the Compute Unified Device Architecture (CUDA) framework.^62^ GPUs are specialized for highly parallel operations based on a Single-Instruction, Multiple-Thread (SIMT) architecture, in contrast to central processing units (CPUs), which process a sequence of threads with only a few tens of threads in parallel. The development of molecular quantum chemical GPU codes began with concentrating on the integral codes of GAMES-UK,^63^ TeraChem,^61^ Gaussian 16,^64^ PySCF,^65^ to mention only a few programs for use in DFT. Taking TeraChem as an example, extensions to time-dependent density functional theory (TDDFT), CASSCF, and CASPT2 followed. Writing GPU-optimized programs can be a tedious task since two goals have to be achieved: (i) The algorithms must take advantage of the highly parallel GPU architecture, and (ii) the data transfer between CPU and GPU should be minimized. In TeraChem, the entire program is practically entirely based on GPU programming, where extensive sections of code had to be newly developed. Other codes followed a more conservative approach in transforming only selected segments of code to GPU usage.

The situation concerning different programming steps is more complex concerning the MR-CISD and the related MR-AQCC methods in COLUMBUS. The sequence of individual steps is illustrated in a block diagram in Figure 5. The wave function optimization step (determining the lowest eigenvalues and corresponding eigenvectors of the Hamiltonian matrix) is typically by far the most time-consuming. It is this step we are concentrating on in this work with the GPU implementation.

**Figure 5:**
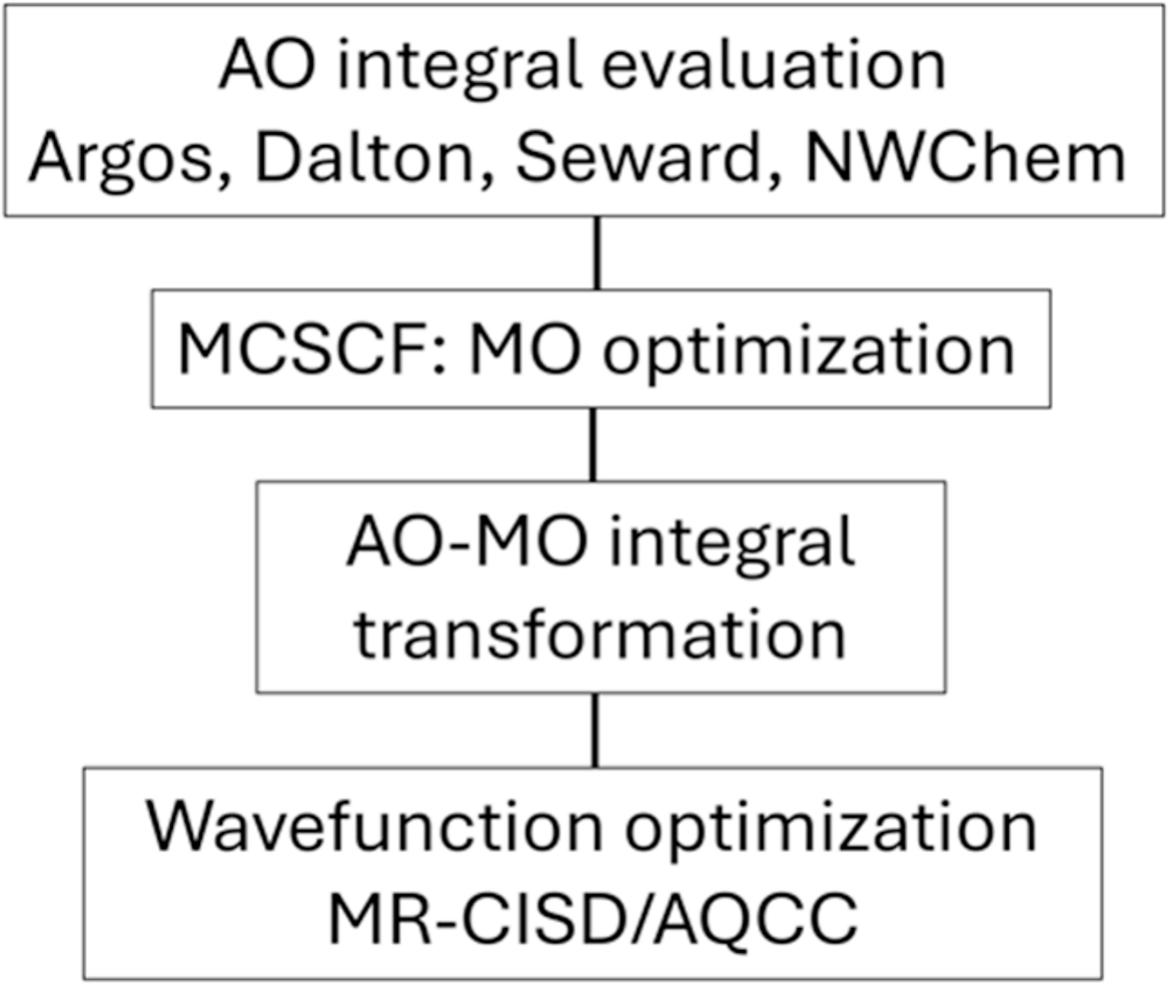
List of individual steps for a single-point MR-CISD/AQCC calculation.

In short, the wave function optimization step in COLUMBUS is based on an uncontracted MR-CISD^50^ calculation using GUGA^7^ with a direct CI technique.^66^ All CSFs are represented in a distinct row table (DRT) shown graphically as a Shavitt graph^7,67^ in Figure 6. This graph is divided into two parts, with the external orbitals at the bottom and the internal orbitals at the top. The external orbitals are limited to a cumulative occupation of up to two electrons, corresponding to the MR-CISD wave function expansion. The external part of the Shavitt graph is much simpler and regular, whereas the internal part can have any desired complexity. Once an internal walk is included in the expansion space, it is always combined with all possible external walks. This results in efficient computational kernels consisting of dense matrix–matrix products, matrix-vector products, and vector dot products with respect to the external orbitals,^8,68,69^ which may be offloaded onto accelerators and GPU hardware. It is noted that with typical modern basis sets, the number of external orbitals is typically much larger than the number of internal orbitals. The walks through the DRT (the CSFs) can be classified according to the passage through one of the four nodes, Z, Y, X, and W, at the interface between internal and external orbitals. Z-walks contain the reference CSFs and other configurations with zero excitation in the external space, Y-walks characterize single excitations, X-walks double excitations where the two external electrons are triplet coupled, and in W-walks the two external electrons are singlet coupled.

**Figure 6:**
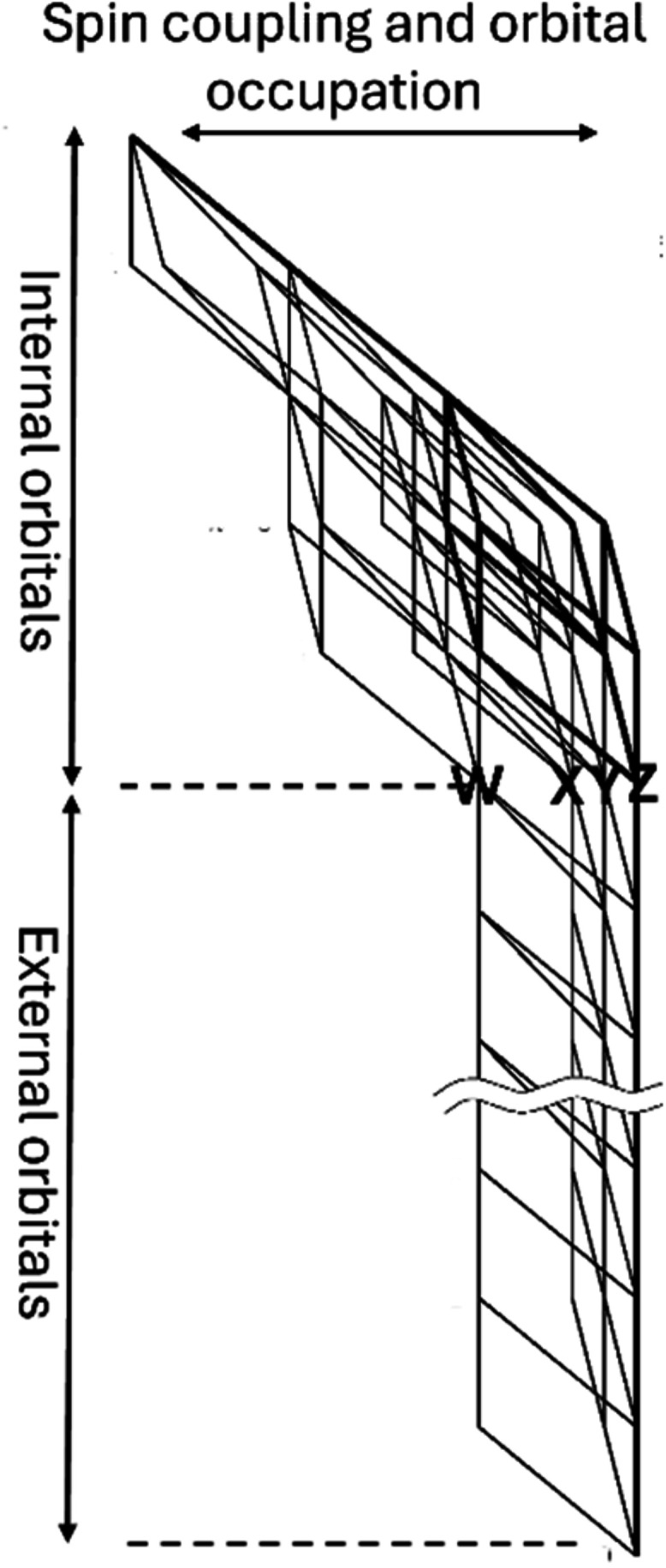
Graphical representation of a typical DRT (Shavitt graph) representing the CSF expansion space for a given set of electrons, spin, and number of orbitals. The lines in bold designate reference configurations. Each distinct, strictly ascending path from the origin to the top describes one of the CSFs of the expansion in terms of cumulative spin-coupling of the orbitals.

The dominant operation within the Davidson subspace method is the calculation of the matrix-vector product

16 w=Hx

of the Hamiltonian matrix **H** with an expansion vector **x** of the Davidson procedure.^70,71^ The matrix-vector products in eq 16 are computed from the underlying one- and two-electron Hamiltonian integrals (*h*_*pq*_, *g*_*pqrs*_) and the coupling coefficients (⟨*m̃′*|*E*_*pq*_|*m̃*⟩, ⟨*m̃*′|*e*_*pqrs*_|*m̃*⟩). The *m̃* denote the different walks (CSFs) through the DRT (Figure 6), and the *E*_*pq*_ and *e*_*pqrs*_ are one- and two-electron excitation operators. The general equation for this operation (eq 16) is

17 $$w_{m\prime ,k}=\sum_{m,\mathrm{pq}}h_{\mathrm{pq}}\left\langle {\mathrm{m̃}}^{\prime }\right|E_{\mathrm{pq}}\left|\mathrm{m̃}\right\rangle x_{m}+\frac{1}{2}\sum_{m,\mathrm{pqrs}}g_{\mathrm{pqrs}}\left\langle {\mathrm{m̃}}^{\prime }\right|e_{\mathrm{pqrs}}\left|\mathrm{m̃}\right\rangle x_{m}$$

The coupling coefficients are computed on the fly as necessary. These coupling coefficients are very sparse, so the procedure adopted in the MRCI code involves the efficient computation of only the nonzero coupling coefficients. The coupling coefficients are computed in blocks based on the partitioning of orbitals and the types of CSFs, W, X, Y, or Z, associated with the respective Hamiltonian block type. The whole computation of eq 17 is driven by the internal indices and the coupling coefficients belonging to a given internal index set. Only the contribution of internal orbitals is computed in this step. The contribution of the external orbitals is calculated on the fly, leading to the matrix multiplication operations shown in Figure 7 (see also refs 8,68 ). For a demonstration of the GPU implementation, we focus on this equation in the most critical section, which is given by walks through the interface nodes X or W leading to the XX, WW, and WX pairs.

**Figure 7:**
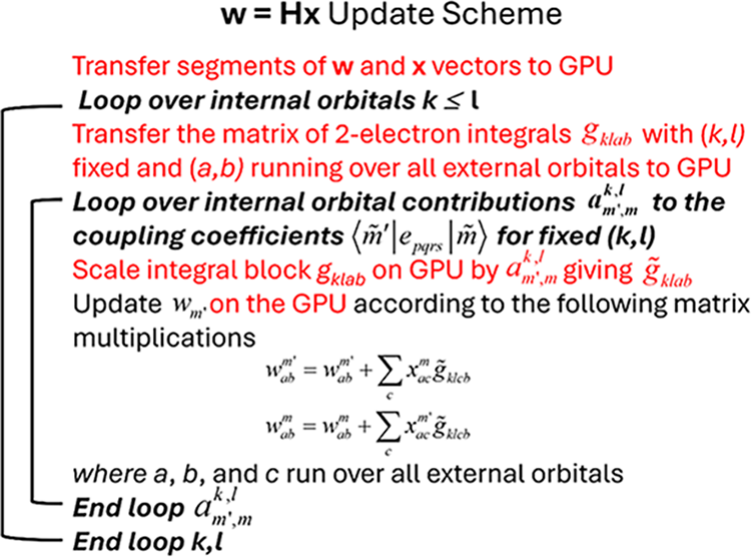
Update scheme for the vector **w** (eq 16) for the special case of CSFs with two external indices and a representative choice of two-electron integral contributions as an example. Operations involving the GPU are in red.

The modular treatment of matrix multiplication kernels via standard subroutine calls to the Basic Linear Algebra Subprograms (BLAS) library was implemented in COLUMBUS some time ago^8^ to facilitate the adaptation of the COLUMBUS code to the evolution of hardware and software components. This strategy is also beneficial for GPU programming. As will be described below, only a few changes in the source code need to be made, such as exchanging the CPU-based BLAS library with a corresponding GPU-optimized CUDA library (cuBLAS) and inserting commands taking care of the (limited) data transfer to and from the GPU.

Figure 7 illustrates this strategy for one representative example. It is shown that with one upload of **w** and **x** vector segments and the transfer of one block of integrals belonging to a fixed internal index pair *kl*, a large number of matrix multiplication updates of the vector **w** (eq 16) can be made coupling all CSFs according to the GUGA formalism. These operations can be performed independently and asynchronously without any further data transfer between CPU and GPU. Thus, only a relatively small effort is required for data transfer of two-electron integrals and CI vector segments, as a large number of matrix multiplications can be performed on the GPU using these data. The number of these multiplications will increase strongly with the increase of the active space since an increasing number of coupling elements will be produced for a set of integral indices.

The GPU implementation is currently based on CUDA-Fortran as provided in NVIDIA’s Compiler suite (NVIDIA HPC SDK).^62^ Variables (matrices and vectors) residing on the GPU are allocated directly using Fortran declarations and variable-to-variable copying, avoiding the need for explicit CUDA function calls. In the current implementation, only device initialization (creating the cuBLAS handle) and GPU call synchronization are performed using dedicated CUDA function calls. These calls can be easily replaced in the future with corresponding functions from other implementations, allowing for straightforward porting of the code to other hardware accelerators that use different programming models. The matrix multiplication itself is performed by calling the corresponding vendor-provided Linear Algebra PACKage (LAPACK) DGEMM routine through a wrapper function. The overhead caused by calling the corresponding BLAS routines on the GPU will be compensated by (a) the speedup of the DGEMM routine for large matrices on the GPU compared to the CPU implementation and (b) the fact that GPU calls can be run asynchronously, meaning the CPU can continue processing the loop over all internal contributions while the multiplication on the GPU is still in progress.

An implementation for handling the two external integrals, as shown in Figure 7, has been recently performed, and benchmarking tests are in progress. Treatment of the other integral types is straightforward and will follow analogous procedures. The current implementation is tightly integrated with COLUMBUS’ approach to parallelization, utilizing the Global Arrays package in conjunction with an MPI-based interprocess communication. This enables the use of multiple GPUs in parallel, thereby mitigating the issue of a fixed, vendor-dependent memory limit standard to contemporary GPUs. The implementation was performed on the Sophia supercomputer at the Argonne Leadership Computing Facility. Preliminary versions of the implementation can be found in a dedicated branch of COLUMBUS’ GitLab repository.

### 3.5 Complex Absorbing Potentials for Electronic Resonances

The ability to describe autoionizing states accurately remains a challenging problem in the electronic structure community. Electronic resonances, a type of autoionizing state, are formed through the resonant attachment of electrons in low-energy electron (LEE)-induced reactions. LEE-induced reactions are prevalent in many areas, including astrochemistry, radiation chemistry, solid-state processes, and others.^72–76^

Electronic resonances are categorized into either single-particle (1p) or two-particle one-hole (2p-1h) resonances. A single-particle shape resonance is formed when a centrifugal potential holds the excess electron bound to the neutral system. These resonances decay through a one-electron process and are characterized by shorter lifetimes.^77^ In 2p-1h resonances, the excess electron is bound to an excited state of the neutral target and, based on the decay mechanism, can be further categorized into “core-excited shape” and Feshbach.^77^ Most studies on electronic resonances in the literature have been limited to shape resonances. Fewer studies focus on Feshbach resonances despite their presence in a wide variety of electron-driven reactions.^78–80^ Additionally, resonant-channel coupling between shape and Feshbach channels facilitates electron-driven processes in nucleobases and similar aromatic systems.^80–82^ A multireference treatment of Feshbach resonances and their mixing with shape channels is pertinent for their accurate characterization.^81,83^ To address this deficiency, an approach based on complex absorbing potentials was recently implemented by creating an interface between COLUMBUS and the OpenCAP software.^84^ COLUMBUS offers several advantages, and its flexibility in describing the multireference space is crucial for describing resonances involving multireference character. Furthermore, the availability of analytic gradients and nonadiabatic couplings makes it an attractive package for future developments involving methods for resonances. The basics of this development are summarized here.

While there are many approaches to describe resonances, we have focused on non-Hermitian quantum mechanics^85^ techniques, where resonances can be obtained as complex solutions to the non-Hermitian eigenvalue problem, with complex (or Gamow–Siegert) eigenvalues of the form

18 $$E_{\mathrm{res}}=E_{R}-i\Gamma /2$$

where *E*_R_ is the energy or position of the resonance state, and Γ is its associated width. Inverse width provides information on the lifetime of the resonance state. In CAP, a non-Hermitian Hamiltonian is obtained by adding a complex local potential to the physical Hamiltonian for a given system^86^

19 Ĥ(η)=Ĥ−iηŴ

where *Ŵ* is a one-particle potential, and η is the CAP strength. A commonly employed one-particle potential in CAP calculations is a quadratic potential (also otherwise known as box-potential) of the form^86^

20 $$\hat{W_{k}}=\{\begin{array}{l} 0,,r_{k}<r_{k}^{0}\\ {\left(\left|r_{k}\right|-r_{k}^{0}\right)}^{2},,r_{k}>r_{k}^{0} \end{array}$$

where *k* = *x*,*y*,*z* and *Ŵ* = ∑_*k*_ *Ŵ*_*k*_. The CAP onset makes sure that the interaction region of the resonance wave function remains unperturbed from the presence of the one-particle potential.

A critical aspect of CAP is the strong parametric dependence of the complex energies on the CAP strength (η). The real and complex part of the eigenvalues as a function of η (η-trajectory) tend to spread out in the complex plane, and the stabilization point is revealed by minimizing the first-order term in the Taylor series expansion of the η-dependent energies. The complex energies corresponding to the stabilized point identify the optimum value.^86^

21 $$\left|E\left(\eta \right)-E\left(0\right)\right|=\left|\eta \frac{dE}{d\eta }\right|+O\left(\eta^{2}\right)$$

The sensitivity of this parametric dependence on η can be further minimized. Many ways have been prescribed for the secondary correction,^86,87^ and one of the straightforward ways is to minimize the second-order correction in the Taylor expansion as proposed by Riss and Meyer^86^

22 $$\left|E\left(\eta \right)-E\left(0\right)-\eta \frac{dE}{d\eta }\right|=\left|\eta^{2}\frac{d^{2}E}{d\eta^{2}}\right|+O\left(\eta^{3}\right)$$

CAP can be introduced into the electronic structure calculation at any stage. The conventional route involves adding the CAP at either the Hartree–Fock (HF) step or the correlated step, depending on the given CAP strength. These calculations are computationally expensive and require modifications to the traditional framework to accommodate changes resulting from the complex algebra involved. Additionally, determining the optimum resonance parameters requires complex-valued energies for multiple values of the CAP strength parameter. Therefore, multiple electronic structure calculations must be carried out to analyze the η-trajectories. An alternative to conventional CAP is the projected CAP, where CAP is projected onto a correlated subspace.^88,89^

We have implemented projected CAP with the MRCI code in COLUMBUS, as outlined in Figure 8. The OpenCAP program by Gayvert and Bravaya was used for CAP calculations.^84^ Details on the projected CAP-MRCI implementation can be found in refs 90,91 . This implementation was tested on several systems and successfully produced complex-valued potential energy surfaces, as well as demonstrated mixing between shape and Feshbach resonances. This approach demonstrated the profound effect the mixing has on the lifetimes of the resonances, with potential implications in the behavior of nucleobases under LEE damage and other relevant applications.^90,91^

**Figure 8:**
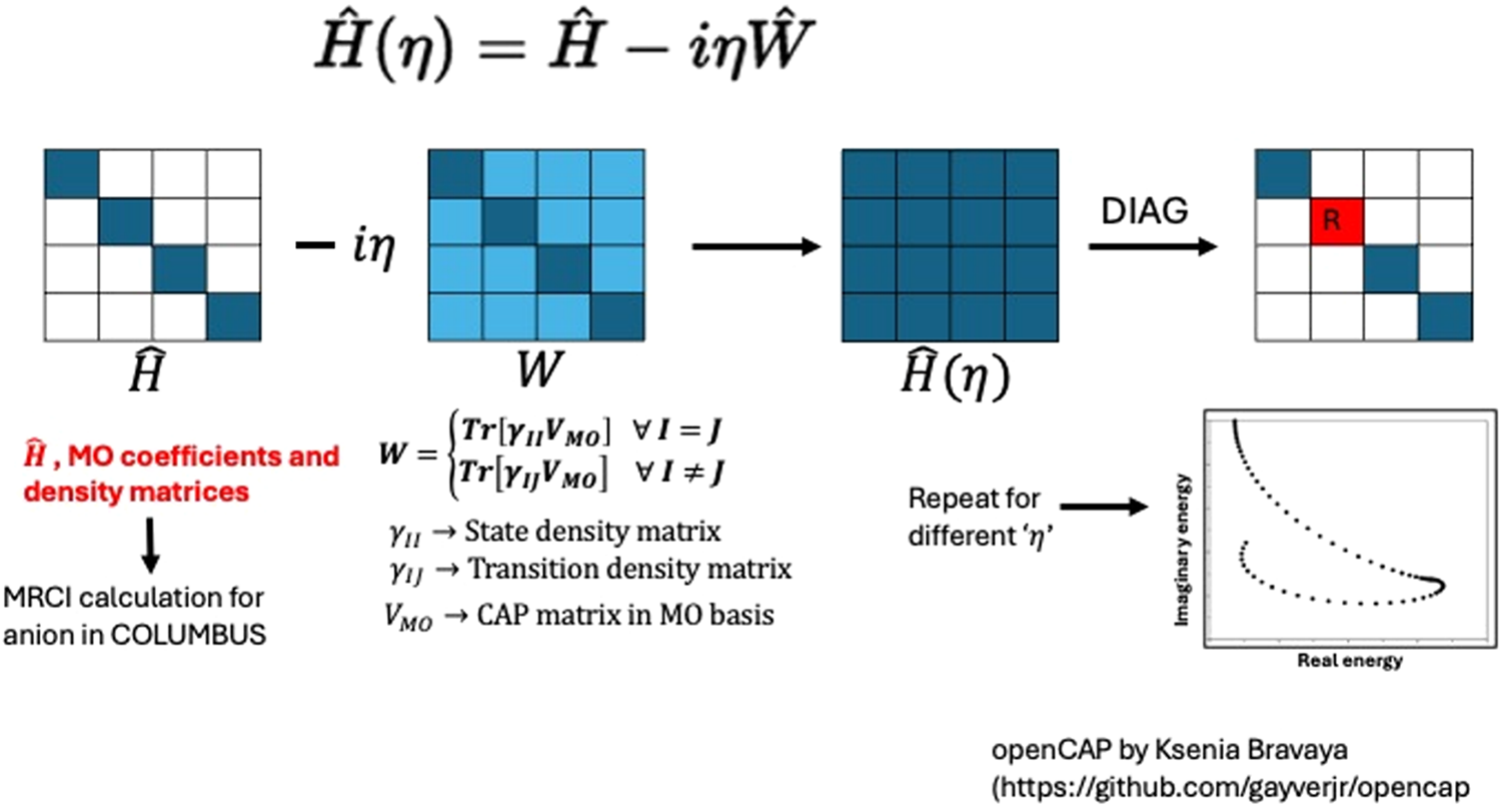
Diagram showing the implementation of projected openCAP in COLUMBUS. Initially, a CAP-free MRCI calculation is performed to obtain a limited number of eigenstates. The one-electron densities, γ_*II*_, and transition densities, γ_*IJ*_ are used to calculate the CAP (*Ŵ*) matrix elements. The complex Hamiltonian *Ĥ*(η) is diagonalized for several values of η and the η-trajectory is used to obtain the Siegert energies.

### 3.6 Hybrid Antisymmetrized Coupled Channels

A customized code version of COLUMBUS delivers the CI functions for applications in attosecond photoemission from molecules in strong laser fields.^92,93^ That process is highly nonperturbative and imposes special requirements on the representation of the system. Emission rates and spectra are susceptible to thresholds and initial and final state correlations.^94^ Therefore, correlated ground and excited states must be accurately represented. In addition, photoelectrons are emitted into a vast spectrum of continuum states, from threshold up to hundreds of eV. For single-electron emission, the CI space is augmented by a complete single-electron basis represented on a numerical grid. An ansatz is made for the time-dependent wave function as a linear combination of one or more neutral states and “channel” functions of ionic states that are augmented by the numerical basis

$$|\Psi ,t\rangle =\underset{N}{\sum }\left|N\right\rangle c_{N}\left(t\right)+\underset{A}{\sum }\left\{\underset{i}{\sum }a_{i}^{†}\left|A\right\rangle c_{i}^{A}\left(t\right)+\underset{\alpha }{\sum }a_{\alpha }^{†}\left|A\right\rangle c_{\alpha }^{A}\left(t\right)\right\}$$ 23

with complex time-dependent coefficients $c_{N}\left(t\right)$ , *c*_*i*_^*A*^(*t*) and *c*_α_^*A*^(*t*). In this “hybrid” representation, the neutral states |N⟩ and the ionic states |*A*⟩ are generated by COLUMBUS in MCSCF-MRCI, based on the molecular orbitals |i⟩ of the *neutral* state at its equilibrium geometry. Using the orbitals at the neutral positions also for the ions is justified in the approximation that relaxation of the nuclear positions occurs on a longer time scale than the electronic emission. The creation operators *a*_*i*_^†^ generate all molecular orbitals. The *a*_α_^†^ generate numerical basis functions {|α_⊥_⟩} that are orthogonalized to the orbital space {|i⟩} as

24 $$|\alpha_{⊥}\rangle =\left(1-\sum_{i}|i\rangle \langle i|\right)|\alpha \rangle$$

When calculating the effect of two-particle operators *T̂* = *T*_*kl,mn*_ *a*_*k*_^†^ *a*_*l*_^†^ *a*_*m*_ *a*_*n*_ in this hybrid basis, one needs up to three-particle density matrices of the ions w.r.t. the molecular basis

25 $$\left\langle a_{i}^{†}A\right|a_{k}^{†}a_{l}^{†}a_{m}a_{n}\left|a_{j}^{†}B\right\rangle =\left\langle A\right|a_{i}a_{k}^{†}a_{l}^{†}a_{m}a_{n}a_{j}^{†}\left|B\right\rangle$$

and similarly Dyson-orbital-like objects with up to 5 indices for matrix elements between the |*a*_*i*_^†^ *A*⟩ and |N⟩ . At present, these are generated by using an (unpublished) version of COLUMBUS that outputs the |N⟩ and |*A*⟩ as lists of determinants and coefficients. The matrix elements of single- and two-particle operators involving the Gaussian-based orbitals |i⟩ and the numerical basis |α⟩ are computed by numerical quadrature. Linear dependence of the basis is controlled by a low-rank representation of the corresponding pseudoinverse using a Woodbury-type formula.

The time-dependent Schrödinger equation is solved with the ansatz ([Disp-formula eq23]) with absorbing boundary conditions. Photoemission spectra are extracted by analyzing outgoing flux using the t-Surff method,^95^ which accounts for the fact that emission spectra are modified by the time-dependent field outside the simulation volume.

Figure 9 shows strong-field photoemission spectra of CO_2_ in an elliptically polarized field at a wavelength of 800 nm (first published in ref 96). Neutral and up to 6 ionic states were used in this calculation. The strong field dominates, and the spectrum does not exhibit any multipolar characteristics. The process is in the intermediate region between tunneling in the strong field and extreme multiphoton emission. Tunneling produces energetically broad distributions modulated by ripples due to multiphoton processes. The effects of ellipticity are most visible in the cuts through the polarization plane in Figure 9c. Symmetries of the HOMO are reflected in corresponding nodes of the photoemission spectra.

**Figure 9:**
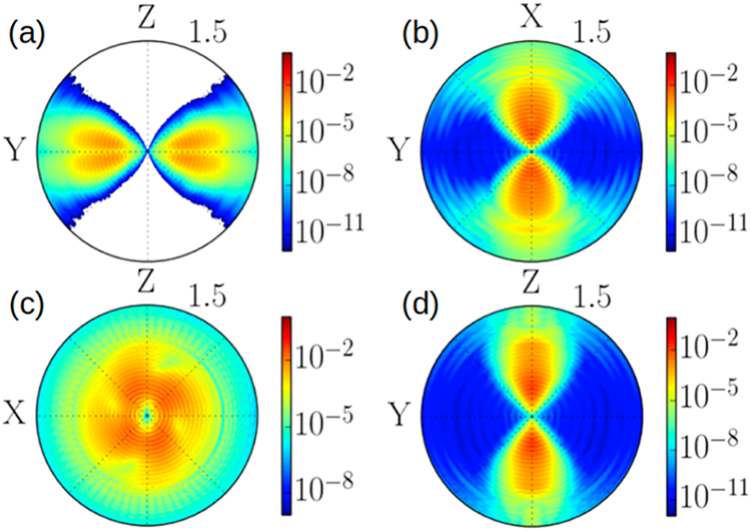
Strong field photoemission from a CO_2_ molecule by a laser pulse with wavelength 800 nm and elliptic polarization. The molecule is aligned along the *Z*-axis, ellipticity is ε = 0.87. The polarization plane is XY in (a) and XZ in (b–d). Energies extend to 1.5 au, and the cut plane is indicated in the subfigures.

## 4 Applications: Open-Shell Systems

### 4.1 Calibration of FOD Parameters Using MR-AQCC Unpaired Densities

Graphene and graphene-based nanomaterials form the basis for numerous interesting applications, including energy storage devices, optoelectronics, flexible conductive films for displays and electrodes, and anticorrosion technology.^97,98^ When broken down into nanoscale fragments, PAHs^99^ are formed, exhibiting notable and tunable diradical and polyradical characteristics.^11,100–103^ These properties make PAHs attractive for spintronics, optoelectronics, conductivity, and magnetism,^104,105^ though their complex electronic structure poses significant characterization challenges. The characterization of these molecules, particularly in terms of their biradical character, provides valuable information for developing new PAH-based materials. The number of effectively unpaired electrons (*N*_U_),^106–108^ accessible through multireference (MR) methods,^20,50^ provides valuable insights into polyradical systems. An alternative descriptor based on DFT calculations is the fractional occupation number-weighted electron density (FOD)^6,109^ obtained from finite-temperature DFT (FT-DFT) without fictitious temperature-dependent energy functionals. This method effectively simulates static correlation straightforwardly based on the electronic temperature (*T*_el_), which acts as a parameter. Although multireference methods can provide reliable information about the open-shell character of PAHs, they are relatively expensive, and DFT-based methods are preferred for large-scale, routine applications. However, in the case of FT-DFT applications, the electronic temperature parameter *T*_el_ needs to be adjusted. In the present report, this adjustment is summarized for a series of functionals through fitting MR-AQCC *N*_U_ values.^110,111^

For optimizing the *T*_el_ parameter, 22 PAHs ranging from acenes, indenoacenes, zethrenes, and other biradicaloid PAHs^110^ were selected and benchmarked using MR-AQCC calculations. The *N*_U_ values and the corresponding unpaired electron densities^106,112^ were obtained using Head-Gordon’s nonlinear formula^107^

26 $$N_{U}=\sum_{i=1}^{N}n_{i}^{2}{\left(2-n_{i}\right)}^{2}$$

Here, *N* is the number of natural orbitals (NOs), and *n_i_* is the occupation of the *i*th NO. This formula emphasizes orbitals with occupations close to one while suppressing contributions near 0 and 2. The geometries of all 22 structures were computed using the TPSS^113^ functional and the def2-TZVP basis set^114^ and are available in the Supporting Information of ref 110.

In the FOD analysis,^6^ the fractional orbital density is defined as

27 $$\rho^{\mathrm{FOD}}=\sum_{i=1}^{N}(\delta_{1}-\delta_{2}f_{i}){\left|\phi_{i}\left(r\right)\right|}^{2}$$

where δ_1_ and δ_2_ are constants. These are both set to unity if the orbital energy is below the Fermi energy (*E*_F_). δ_1_ is set to 0 and δ_2_ to −1, respectively, if the orbital energy is above *E*_F_. The ϕ_*i*_ terms represent molecular spin orbitals, and the summation extends over all orbitals *N*. The *f_i_* values are derived from the Fermi–Dirac distribution, providing fractional orbital occupation numbers as

28 $$f_{i}=\frac{1}{e^{\left(\varepsilon_{i}-E_{F}\right)/kT_{\mathrm{el}}}+1}$$

where ε_*i*_ represents the orbital energy. The number of “hot” electrons, *N*_FOD_, is defined as

29 $$N_{\mathrm{FOD}}=\sum_{i=1}^{N}\left(\delta_{1}-\delta_{2}f_{i}\right)$$

In the original work,^6^ the electronic temperature (*T*_el_) was determined as a function of the percentage of nonlocal Fock exchange admixture (*a*_*x*_) in the functional

30 $$T_{el}=20000\;K\times a_{x}+5000\;K$$

The plot in Figure 10a shows that for the M05–2X functional (*a*_*x*_ = 0.56), there is a good linear correlation between *N*_FOD_ values and *N*_U_ values, using the original *T*_el_ parametrization of eq 30. However, the original *T*_el_ value is much too large leading to a strong overestimation of the *N*_FOD_ values in comparison to respective *N*_U_ data. Optimization of *T*_el_ in steps of 1000 K and refinement in steps of 200 K, the slope is improved, and at 12,200 K, a slope close to one is achieved (Figure 10b).

**Figure 10:**
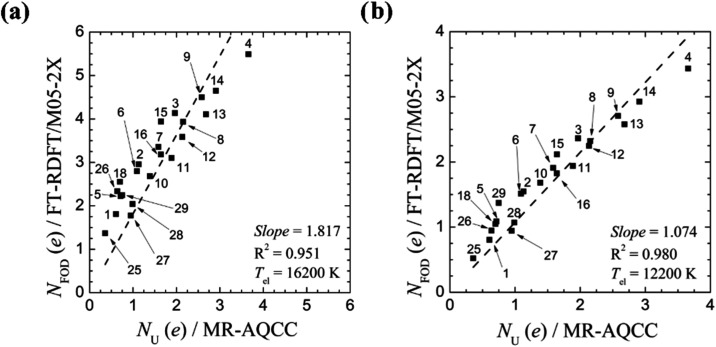
Comparison between MR-AQCC *N*_U_ values and FT-DFT *N*_FOD_ numbers with M05–2X density functional for structures with singlet ground electronic state (structures **1**–**16**, **18**, **25**–**29** of ref 110) using (a) the original *T*_el_ of 16,200 K and (b) the improved *T*_el_ of eq 31 of 12,200 K. Reproduced with permission from ref 110. Copyright 2023 the Royal Society of Chemistry.

Similar investigations with the TPSS, M06–2X, and B3LYP functionals revealed that better agreement could be achieved using the improved equation for these classes of functionals as well^110,111^

31 $$T_{el}=10762\;K\times a_{x}+6140\;K$$

Further tests indicated that this improvement extends to the double-hybrid functional B2PLYP as well.^111^

Range-separated hybrid functionals have also been investigated.^111^ Application of eq 31 developed for GGA functionals to the range-separated ωB97XD functional (*a*_*x*_ = 0.222) leads to a *T*_el_ of 8530 K. Although the correlation between the corresponding *N*_FOD_ and *N*_U_ values shows good linearity (Figure 11a), the slope is much too small. By increasing *T*_el_ to 14,000 K, an excellent overall correlation is achieved (Figure 11b).

**Figure 11:**
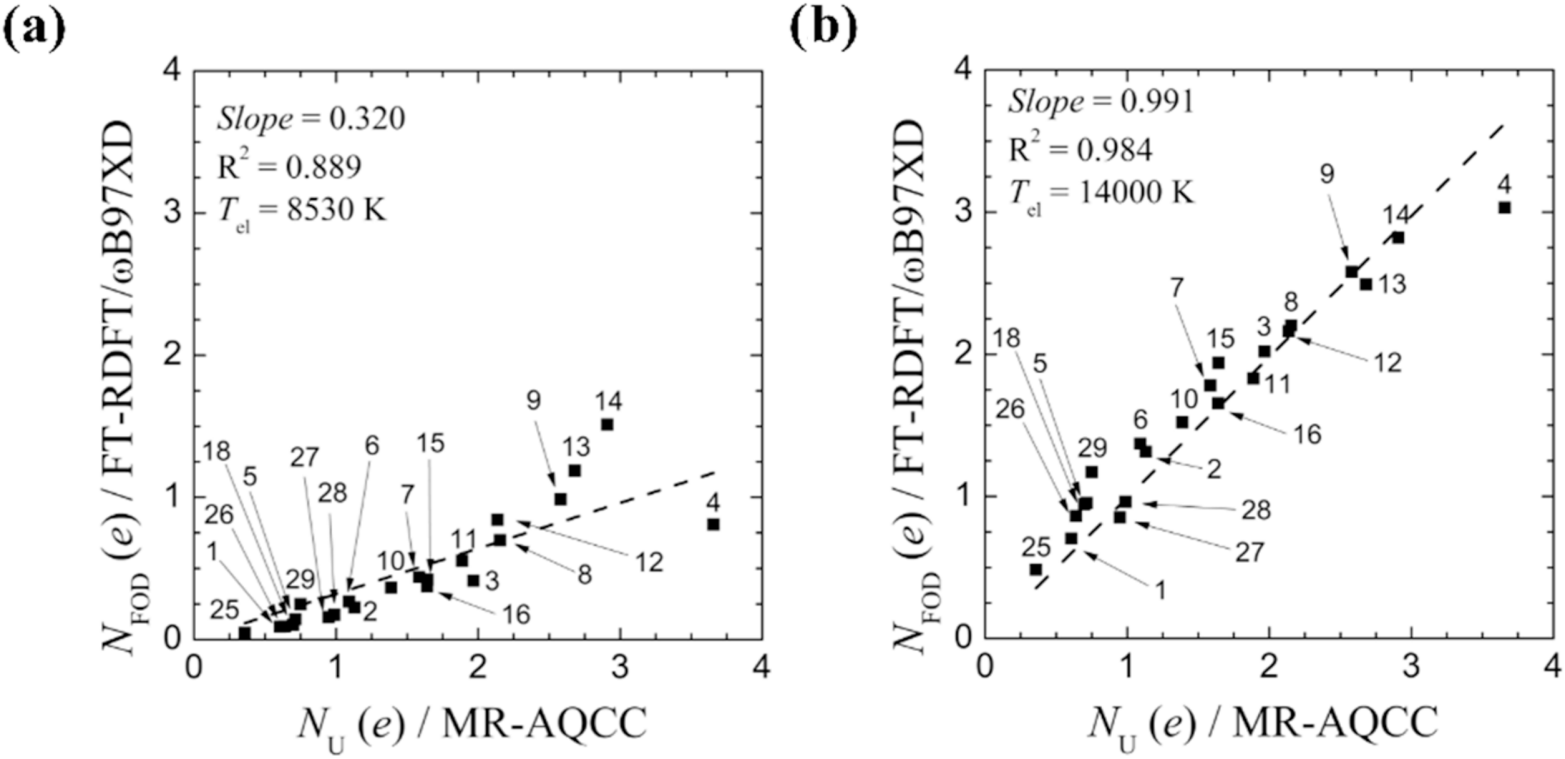
Comparison between MR-AQCC *N*_U_ values and FT-DFT *N*_FOD_ numbers for the ωB97XD density functional for structures with singlet ground electronic state (structures **1**–**16**, **18**, **25**–**29** numbered as in ref 111) using (a) *T*_el_ of eq 31 of 8530 K and (b) the improved *T*_el_ of eq 32 of 14,000 K. Reproduced with permission from ref 111 under a Creative Commons Attribution 4.0 International License. Copyright 2024 Carvalho et al.^111^

The same adjustments of *T*_el_ were performed for the range-separated functionals ωB97M-V, CAM-B3LYP, LC-ωPBE, and MN12-SX. It was found that the resulting *T*_el_ values could be fitted against the β parameter, which is the difference between the HF exchange in the long-range limit (α + β) and the corresponding percentage in the short-range limit^115^ (α) leading to the expression.^111^

32 $$T_{\mathrm{el}}=6650\;K\times \beta +9010\;K$$

In addition to the parametrization of *T*_el_ for various types of DFT functionals, an optimal Fermi temperature has also been determined for the semiempirical GFN2-xTB method,^116,117^ allowing reliable *N*_FOD_ data at even much faster computational speeds.^111^ In summary, a set of Fermi temperature parameters *T*_el_ has been derived for a range of DFT functionals based on a larger selection of open shell PAH compounds, leading to *N*_FOD_ values that reproduce the MR-AQCC *N*_U_ values very well. This opens up excellent opportunities for computationally efficient predictions of the biradical character, especially for larger molecules. So far, the investigations have focused on PAHs with applications also to B,N doped periacenes (see also Section 5.2) and CC dissociation processes in ethane, ethylene and acetylene.^111^ In all these cases good agreement with the number of unpaired electrons *N*_U_ were found. Thus, we expect that carbon biradicals and similar systems should be well covered by the present *T*_el_ parametrization. To demonstrate its applicability beyond this set of compounds, more extended classes of molecular structures still need to be investigated.

### 4.2 Electronic States in Single-Vacancy (SV) Graphene Defects

Graphene is renowned for its exceptional electronic, thermal, and mechanical properties.^97^ Doping of graphene sheets is frequently used as an efficient tool to modulate their electronic properties. One important class of graphene defects is carbon vacancies, which can occur either naturally due to growth defects or by collisions with high-energy species.^118^ The removal of a carbon atom from the regular graphene network creates dangling bond carbons, leading to low-lying electronic states.^119,120^ The detailed structural characterization of these states and their interplay with geometrical changes comprise interesting but challenging questions. Reliable approaches require multireference methods (MR) that describe both the ground and excited states equally well. MR-CISD,^12,50,51^ as available in COLUMBUS, provides a good basis for such investigations. This contribution summarizes our previous work on a single vacancy defect in pristine pyrene.^119–122^ By removing one carbon atom from the center of pyrene (Figure 12a) leading to pyrene-1C, three dangling bonds are formed upon rigid C removal (Figure 12b). On planar geometry relaxation, two interact to form a covalent bond, leaving only one dangling carbon bond (Figure 12c). Planar pyrene-1C has a quasi-degenerate ground state with three low-lying excited states. This finding prompted us to investigate further whether out-of-plane distortions could result in additional stabilization and how excited states could be affected.^122^

**Figure 12:**
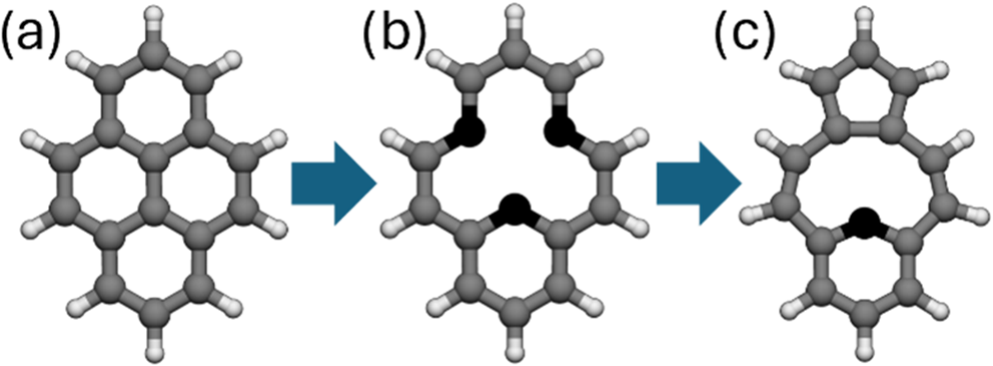
(a) Pristine pyrene, (b) unrelaxed pyrene-1C, carbon dangling-bond centers are shown in black, (c) pyrene-1C after geometry relaxation.

The geometry of pyrene-1C was fully optimized in *C*_2*v*_ symmetry using a CASSCF­(16,16) approach that included all π-orbitals and the carbon dangling bond for each of the four lowest states (^1^A_2_, ^3^A_2_, ^1^B_1_, and ^3^B_1_). The CAS was then systematically reduced to a CAS­(6,6) and finally to a compact CAS­(4,3) that was able to reproduce the CAS­(16,16) excitation energies and geometries well. Final calculations were performed at SA4-CASSCF­(4,3) (averaged over the four lowest states) and MR-CISD+Q­(4,3) (+Q indicates Davidson correction^15,51^ ) levels using the 6–31G* basis set.^123^ All stationary structures described in this work were characterized by harmonic frequencies calculated at the SA2-CASSCF­(4,3)/6–31G* level.

The *C*_2*v*_ structures optimized for the ^1^A_2_, ^3^A_2_, ^1^B_1_, and ^3^B_1_ states were all found to be transition states, with ^3^B_1_ being more stable than the others by less than 0.2 eV. Following the imaginary modes, four nonplanar optimized structures with *C*_*s*_ symmetry were obtained, of which states 1A′ and 3A″ were transition state structures, and ^1^A″ and ^3^A′ were local minima. Further distortion of ^1^A′ from *C_s_* to *C*_1_ symmetry resulted in a ^1^A structure, whereas the distortion of ^3^A″ led to a ^3^A′ structure. An energy diagram of these structures is presented in Figure 13. Among these structures, the L-shaped conformation of the ^1^A′ state, obtained from the distortion of the ^1^B_1_ state, was considerably more stable than the other distorted *C*_2*v*_ structures. Its stabilization amounted to 1.13 eV in comparison to the ^3^B_1_ structure. The higher stability of this conformation is derived from a 3c-2e bond formed between the carbon of the dangling bond and a C–C bond of the cyclopentadienyl fragment. Despite the stability of this structure, it was still not a local minimum on the potential energy surface. Further reduction of the symmetry to C_1_ allowed the localization of the 3c-2e bond into a σ-bond between the dangling of the carbon bond and a carbon of the cyclopentadienyl ring. This structural rearrangement led to a highly distorted structure, which, nevertheless, is about 3.77 eV lower in energy compared to the reference ^3^B_1_ structure because of the formation of a CC σ–bond. Calculating a triplet state using the ^1^A geometry as a starting point resulted in a similar geometry but 1.55 eV higher in energy.

**Figure 13:**
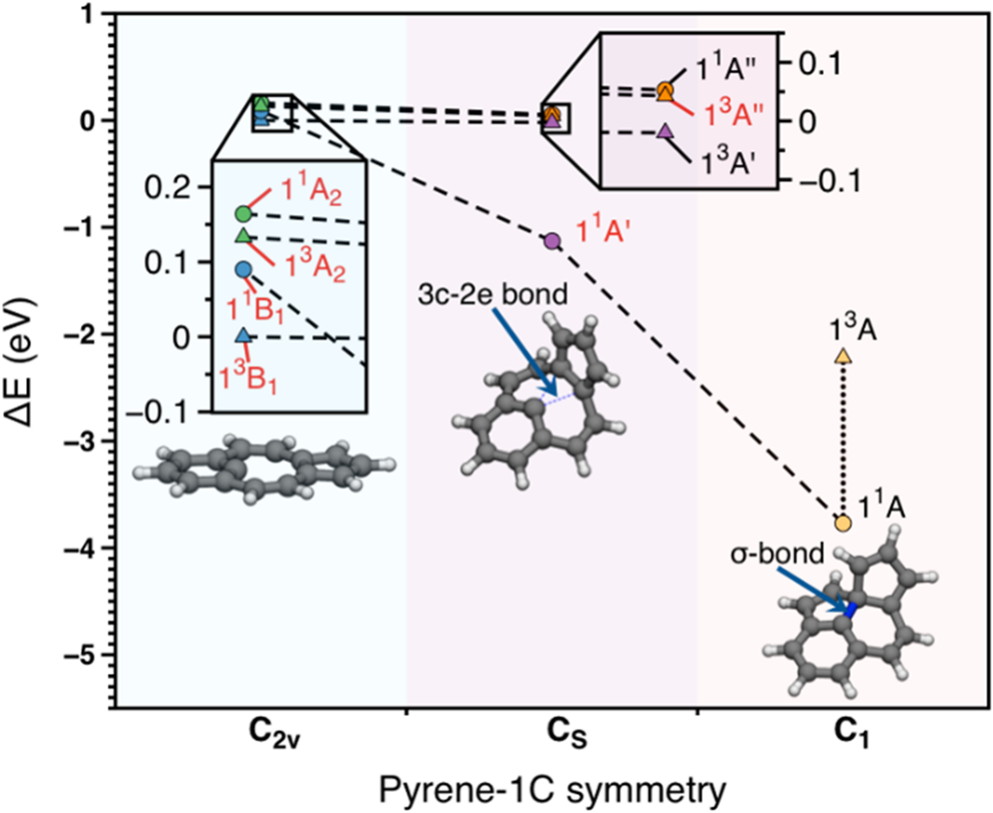
Energies Δ*E* (eV) are relative to the ^3^B_1_ state using the MR-CISD+Q­(4,3) method for the optimized geometries of the ^1^A_2_, ^3^A_2_, ^1^B_1_, and ^3^B_1_ states in *C*_2*v*_ symmetry, the optimized geometries of the ^1^A′, ^3^A′, ^1^A″, and ^3^A″ states in *C*_*s*_ symmetry and for the ^1^A and ^3^A states in *C*_1_ symmetry. Transition states are labeled in red.

Vertically excited states for the symmetries B_1_ and A_2_ of the lowest singlet states are shown in Figure 14a, computed at the *C*_2*v*_ geometry of the ^3^B_1_ structure. There are three excited states below 1 eV, while the remaining are all above 4 eV relative to the ground state. The most intense excitation, calculated from the 1^1^B_1_ state, is to the 4^1^A_2_ state, with an excitation energy of about 7.8 eV. A similar pattern is observed for the other *C*_2*v*_ and *C_s_* optimized structures. The only exception is the ^1^A′ optimized structure, which has a closed-shell configuration due to the formation of a 3c-2e bond, resulting in a wide gap between the ground state and the first three excited states, which are about 3 eV higher in energy (Figure 14b). The remaining excited states are all above 5 eV. Two intense excitations are observed from the 1 ^1^A′ state: one to 3^1^A″ and the other to 4^1^A″.

**Figure 14:**
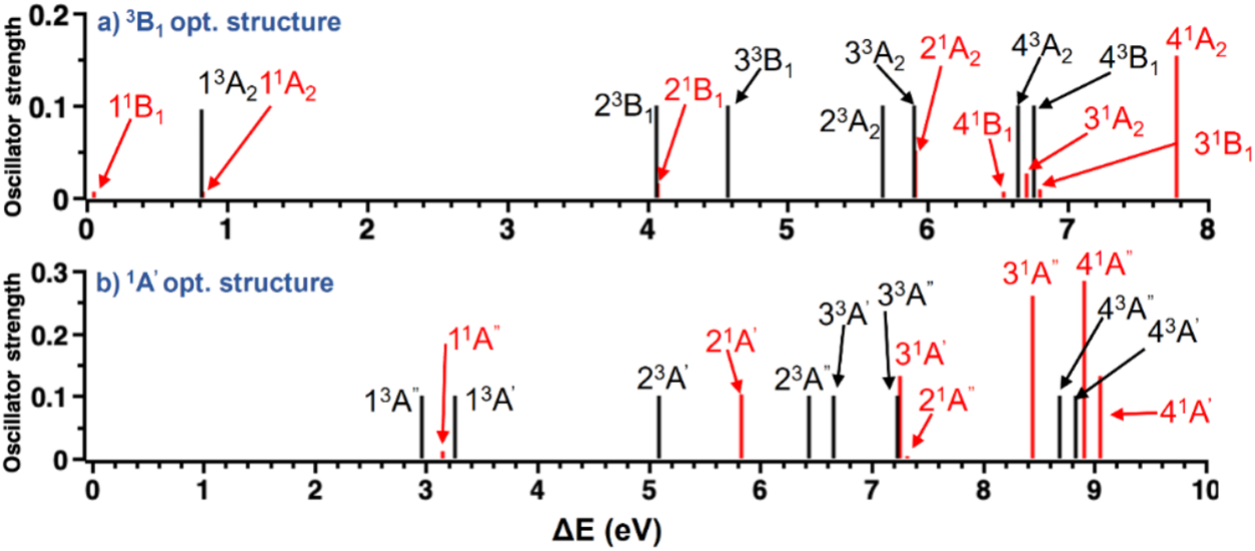
Vertical excitation energies Δ*E* (eV) relative to (a) the optimized ^3^B_1_ structure and (b) to the ^1^A′ structure computed at the MR-CISD+Q­(6,6) level. Oscillator strengths refer to the singlet states (represented by the red vertical lines). For triplet states, black vertical lines only indicate the energetic position of the electronic state. Adapted with permission from ref 122. Copyright 2023 American Chemical Society.

A systematic distortion of the planar pyrene-1C structure resulted in several stationary points from which four structures were minima. A highly distorted closed-shell ^1^A structure with C_1_ symmetry was the most stable one, with 3.77 eV more stable than the planar ^3^B_1_ reference structure, followed by an open-shell ^3^A structure, which has a similar geometry to ^1^A but is 1.55 eV higher in energy. The other minima (^1^A″ and ^3^A′), which are less distorted compared to the planar structure but have a dangling bond, are at most 0.02 eV more stable than the original ^3^B_1_ reference structure. In larger sheets, greater restraining forces will exist, certainly disfavoring the *C*_1_ symmetry structure. The others should be more adaptable in their current structures. Investigations into larger graphene sheets will be necessary to resolve this question conclusively.

### 4.3 Construction of Polyradical Character with Phenalenyl Oligomers

Carbon-based radicals have recently been the subject of intense study due to their unique electronic and magnetic properties, as well as their potential applications in materials chemistry and biosciences.^124^ The high chemical reactivity of radicals presents a considerable challenge for their synthesis and characterization, often necessitating a combination of solution and on-surface chemistry.^125^ Among these, the structures composed of benzenoid rings arranged in triangular patterns are regarded as promising materials in spintronics applications,^126–128^ and for the design of thermally active delayed emitters.^129^

A small singlet–triplet (S_0_/T_1_) energy gap (Δ*E*) is the main prerequisite for a desirable magnetic switching in these materials.^130^ Despite their reactivity originating from open-shell electronic structures, triangle-shaped systems, and their two-dimensional extensions have been synthesized and characterized using advanced on-surface approaches.^128,131–134^ Studies of triangulene dimers, directly coupled or linked via bridging groups such as acetylenes and phenyl, demonstrate that the nature of these connections can tune their magnetic properties and ground-state spin states well.^127,134–139^ The triangle-shaped radical compounds and their extensions form alternant π-conjugated systems and belong to the class of non-Kekulé structures. In these structures, the spin quantum number (*S*) of the ground state is predicted according to Ovchinnikov’s rule^140^ and Lieb’s theorem^141^ as

33 $$S=\frac{N^{*}-N}{2}$$

where N* and *N* are the number of starred and unstarred sites in the alternant π-conjugated systems, respectively.

In this contribution, we report on an initial study on covalently linked dimers of phenalenyl, the smallest member of triangle-shaped molecules with highly delocalized radical structure (Figure 15a), high electrical conductivity, and distinctive magnetism.^142–146^ The α positions label those atoms where the spin density in the singly occupied molecular orbital (SOMO) is located, whereas β atoms do not carry spin density. Due to their size, phenalenyl dimers are ideal for studying how the different linking types between phenalenyl units influence their electronic properties. Depending on the coupling sites, the following structural isomers can be identified: (i) isomers coupled with the spin positions (α,α-dimer **1**, Kekulé structure), nonspin positions (β,β-dimer **2**, non-Kekulé structure), and spin-nonspin positions (α,β-dimer **3**, non-Kekulé structure), illustrated in Figure 15b. All three structures are alternant π-conjugated systems. Applying Ovchinnikov’s rule to these systems suggests a singlet ground state for α,α- and β,β-dimers, while the ground state of the α,β-dimer should be a triplet spin state. This contribution discusses the electronic structure of the three isomers linked via an acetylene bridge. For this purpose, extended multireference calculations were performed to evaluate the singlet–triplet splitting energy in comparison with the prediction by Ovchinnikov’s rule and to characterize the open shell character of the dimers employing numbers of unpaired electrons (*N*_U_).

**Figure 15:**
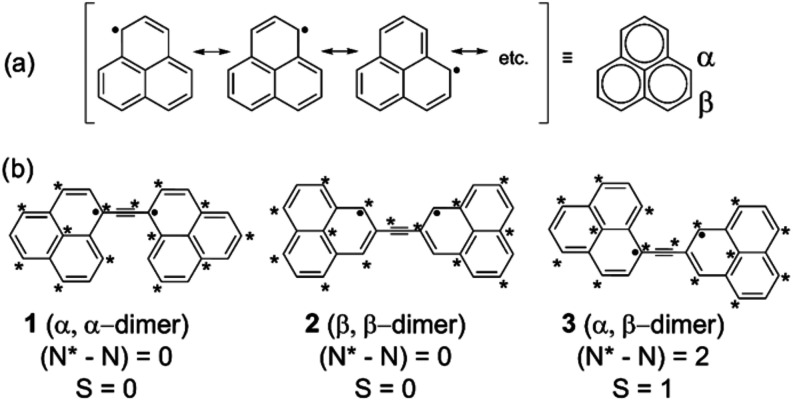
(a) Phenalenyl: α and symmetry equivalent C atoms denote positions with SOMO contributions, whereas β positions do not contribute to the SOMO, (b) phenalenyl dimers **1**–**3** linked according to α/β positions; spin multiplicity given according to Ovchinnikov’s rule.

The calculations were performed using the MR-AQCC^20^ method in combination with the orbitals obtained from a state-averaged (SA)-complete active space self-consistent field theory consisting of eight electrons in eight orbitals (SA-CASSCF­(8,8)). The same CAS was used as reference space in the MR-AQCC calculation. The reference space has been chosen based on the NO occupations by including the main open-shell orbitals within an occupation range of ∼1.90 e to ∼0.1 e. The σ space orbitals were kept frozen after the SCF level, while all π orbitals were included, as has been successfully used for a series of PAHs in previous investigations.^11,100,147^ All calculations were performed with the 6–31G­(d) basis set.^148^ The singlet structures were optimized at the unrestricted density functional theory (UDFT) level using the B97-D functional^149^ and the TZVP basis set.^114^ S-T splittings were calculated as Δ*E*_ST_ = *E*_T_ – *E*_S_, computed as the vertical energy difference between the lowest singlet (*E*_S_) and triplet (*E*_T_) states, where the positive values indicate that the singlet is the lower energy state. A measure for the total number of unpaired electrons (*N*_U_) was calculated using eq 26.

In agreement with the results on directly coupled dimers^138^ and according to Ovchinnikov’s rule (eq 33) and Figure 15b, the number of star and nonstar sites assigns a singlet spin state to dimers **1** and **2** and a triplet spin state to dimer **3**, assuming that the acetylene bridge is involved in conjugation. S-T splitting energies are collected in Table 2. A large positive S-T splitting value is found for **1** (Δ*E*_ST_ = 0.92 eV). It is related to a strong interaction due to the involvement of α atoms as connectors between the two phenalenyl rings. This case aligns well with Ovchinnikov’s rule, which predicts that **1** should have a singlet ground state. A slightly negative value is observed for **3** (Δ*E*_ST_ = −0.004 eV), predicting a quasi-degeneracy of the singlet and triplet states. Formally, the prediction by the Ovchinnikov rule is correct but misses the quasi-degeneracy. A tiny negative splitting is found for **2** (Δ*E*_ST_ = −0.003 eV), which implies a quasi-degeneracy as well as slightly favoring the triplet state again. This contradicts the prediction based on the Ovchinnikov rule. The *N*_U,S_ values (Table 2) are significantly larger for both **2** and **3** than for **1**. They are about twice the *N*_U_ value of phenalenyl (1.36 e) and indicate weak interactions between the phenalenyl units in these two cases. *N*_U,T_ values are almost identical to the singlet ones for structures **2** and **3**. The stronger interaction between the two phenalenyls in **1** reduces the *N*_U,T_ value somewhat.

**Table 2:** Singlet-Triplet Splitting Energies and *N*_U,S_ (Singlet State) and *N*_U,T_ (Triplet State) Values for 1-3 Computed at MR-AQCC Level

	**1**	**2**	**3**
connection	α,α	β,β	α,β
Δ*E*_ST_ (*e*V)	0.924	–0.003	–0.004
*N*_U,S_ (e)	1.66	2.70	2.61
*N*_U,T_ (e)	2.58	2.70	2.63

Figure 16 displays the calculated unpaired densities for the three isomers, showing significant differences in the distribution of unpaired electrons in the acetylene bridge. The unpaired density for the singlet state is intensely concentrated in the (α,α)-type bridge in **1** (Figure 16a), reducing the radical character of the phenalenyl rings. On the contrary, the other two connection types (β,β) and (α,β) (Figure 16b,c) do not lead to any (in **2**) or minimal (in **3**) unpaired densities in the acetylene bridge. The unpaired density for the triplet state of **1** (Figure 16d) shows a significant open-shell character localized in the phenalenyl rings, in contrast to the respective singlet case (Figure 16a). There is also substantial unpaired density localized in the acetylene bridge. The unpaired densities for **2** and **3** (Figure 16e,f) are practically identical to the open-shell singlet counterparts.

**Figure 16:**
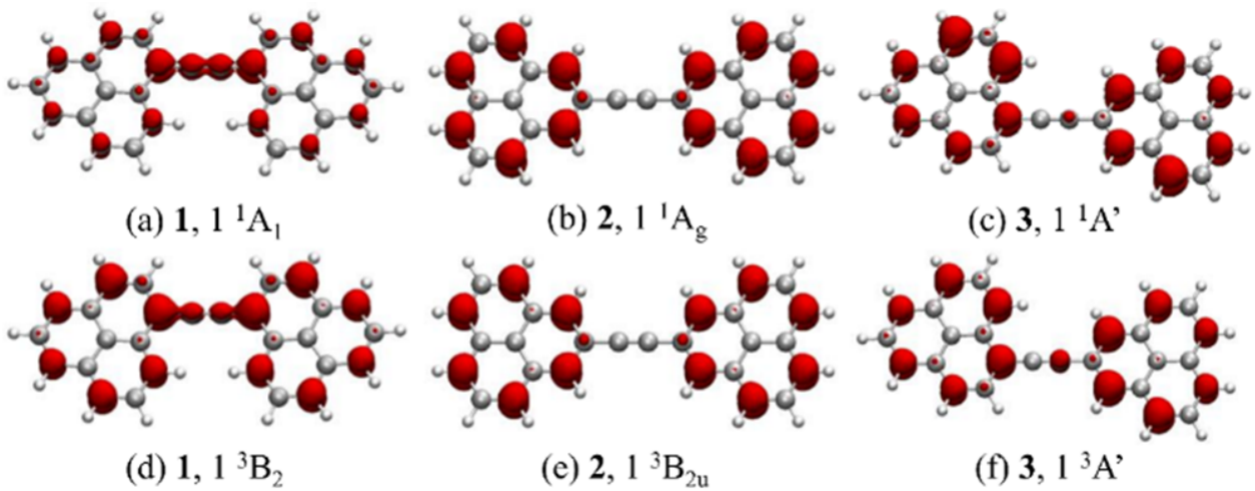
Unpaired electron densities (isovalue 0.004 e/Å^3^) for the singlet and triplet states of **1**–**3**.

The results for different isomers of phenalenyl dimers connected by an acetylene bridge reveal the variability in their electronic structure and, consequently, their magnetic properties, depending on the type of connection. The connecting bridge plays a crucial role in these variations. Furthermore, it is suggested that the different roles of the bridge, particularly its interference with the radical character, may cause deviations from the spin predictions based on the Ovchinnikov rule in cases where the interactions between the two radical subsystems are weak due to the involvement of β-type connectors. Ovchinnikov’s rule will always favor one multiplicity. In contrast, our results show that in some cases, singlet and triplet states can be quasi-degenerate, and both should be considered on equal footing. This situation highlights the importance of high-level computational studies performed at the multireference level.

### 4.4 Aromatic and Heteroaromatic Polyradicals

Polyradicals emanating from aromatic and heteroaromatic rings are an interesting class of molecules; highly reactive due to the presence of dangling, unpaired electrons yet thermodynamically stabilized by the aromaticity present in the ring.^150^ Such polyradicals can be formed via the electrocyclization of acyclic precursors^151^ or found in high-temperature combustion of cigarette smoke or alternative fuels.^152^ They are also vital intermediates in the fabrication of polymeric materials.^153^ The reactivity and fleeting existence of these species complicate their experimental characterization, while the complexity of their electronic structure necessitates careful theoretical analysis. For instance, using polyradicals containing two unpaired electrons as an example, two electrons in two orbitals give rise to six electron configurations. These electron configurations combine to form four electronic spin states: a triplet and three singlet states.^37,154^ Each of the singlet states requires more than one electron configuration (as represented by a Slater determinant) to fully capture the physical nature of each singlet state.

The proper characterization of these multiconfigurational singlet states requires multireference (MR) methods involving initial guess wave functions that contain more than one determinant. The multireference and complete active space (CAS) methods available in COLUMBUS are well-suited to describing multiconfigurational systems. Additionally, the energy manifolds of polyradicals contain a high density of closely lying states. In these instances, the state-averaging available in COLUMBUS enables the development of a state-averaged, multireference wave function that is optimized in an averaged manner for all states. That reference wave function is ideal for subsequent single-state analysis using the highly correlated levels of theory available in COLUMBUS, such as MR-CISD and MR-AQCC. The parallel implementation and analytic gradients available in COLUMBUS allow for the characterization of relatively large molecules.

We have used COLUMBUS to characterize the excited states of *para*-benzyne,^155^ and compared the geometries, energies, and electronic structures of the didehydro isomers of benzene,^156^ pyrazine,^157^ thiophene,^158^ and furan. We have also utilized COLUMBUS to investigate aromatic tetraradicals formed through a double Bergman cyclization. The ability to perform geometry optimizations on molecules of this size has enabled us to report both adiabatic and vertical excitations, as well as analyze singlet–triplet gaps and through-bond and through-space couplings of the radical electrons. Additional features available in COLUMBUS utilized in these analyses included the determination of natural orbitals, unpaired electron densities, and spin densities.

We employed the state-averaging approach in COLUMBUS to demonstrate that *para*-benzyne exhibits a very dense manifold of excited valence and Rydberg states within a 10 eV window of the singlet 1Ag ground state.^155^ In addition, spin-specific state averaging was instrumental in characterizing the 36 different spin states (1 quintet, 15 triplets, and 20 singlets) arising from 70 distinct electron configurations of a 1,4,5,8-tetra-dehydro-naphthalene. These results suggested a large (4 eV) gap between states 6 and 7 (Figure 17b), which then allowed us to focus our state-specific characterization efforts on the six lowest states.

**Figure 17:**
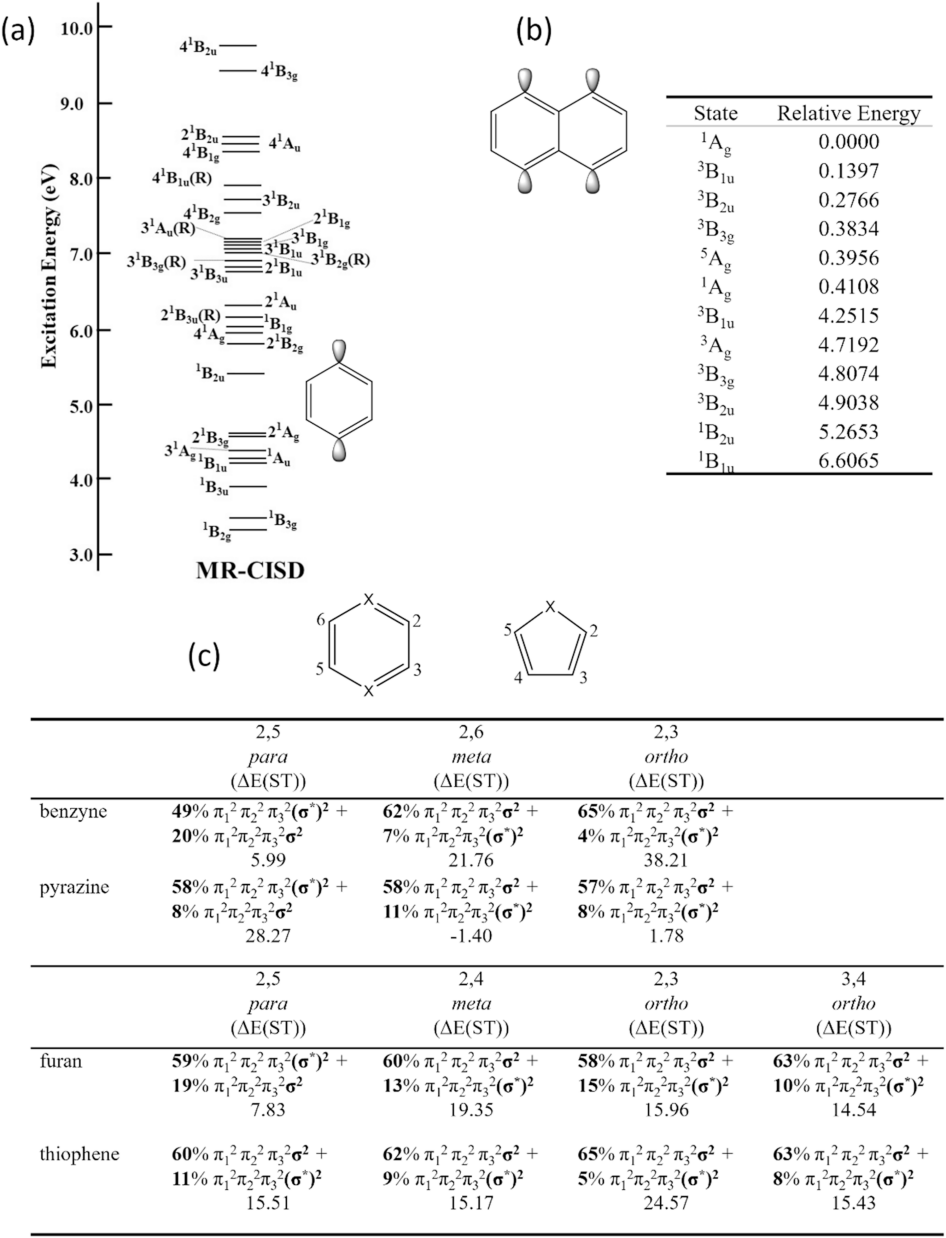
Examples of aromatic and heteroaromatic polyradicals characterized using COLUMBUS: (a) *para*-Benzyne contains a high density of valence and Rydberg excited states within 10 eV of the 1Ag ground state. Manifold determined using CAS (8,8) MRCI/TZ with 32-state averaging; (b) distribution of excited states for 1,4,5,8-tetradehydronapthalene computing using state-specific, state-averaged CAS (6,6)/TZ geometry optimization; (c) dominant wave function configurations and singlet–triplet splittings (kcal/mol) for aromatic and heteroaromatic diradicals computed using singlet state MR-AQCC/TZ calculations and active spaces comprised of π and σ orbitals (CAS (8,8) for benzyne; CAS (8,7) for furan and thiophene, and CAS (12,10) for pyrazine including out-of-plane lone pair electrons on nitrogen atoms). Including the out-of-plane lone pair electrons on sulfur atoms in thiophene did not produce a significant difference, likely due to the mismatch in orbital energies.

We also utilized COLUMBUS to understand the multiconfigurational character and singlet–triplet splitting in 9,10-didehydro-anthracene as well as in all didehydro isomers of pyrazine, benzyne, and thiophene (Figure 17c). We found that all these aromatic diradicals are two-configurational with wave functions comprising double occupation of the π orbitals and either the in-phase (σ) or out-of-phase (σ*) linear combination of the radical lobes. For the *ortho* and *meta* isomers, the dominant contribution to the wave function occupies the σ orbital. In contrast, for the *para* isomers, the σ* component has the larger percentage for all systems except thiophene. The *para* isomers display the most significant through-bond coupling, while the *ortho* orientation typically results in the strongest through-space interaction. *meta* Isomers display the most distorted molecular geometries, likely to optimize through-space interactions in systems where through-bond coupling is minimal.

### 4.5 Ionic Bonds in Authentic Hydrocarbons

Since 1985, salts consisting solely of C and H have been isolated and characterized.^159–162^ These results represent a breakthrough in the study of chemical bonds and indicate that steric crowding between the ions, along with an extended charge delocalization over at least one of the ions, are the two main factors that effectively suppress the formation of any covalent bond between the ions in the solid. In the limit of very extended charge delocalization over the two ions along with extreme steric congestion between the ions, one also has the suppression of any covalent bond in the solution.^161,162^

The study of ion pairs is crucial for understanding ionic compounds. In this context, some of the authors of this review recently studied a reduced model system,^164^ which represents a good approximation to the steric repulsion between the tricyclopropylcyclopropenylium cation and Kuhn’s anion studied in ref 160 It has been suggested through CASSCF and DFT calculations that a kinetically stable hydrocarbon ion pair can be formed from the corresponding radicals.^164^ A kinetic model to study the time dependence of its concentration has also been developed.^163^ DFT calculations indicate that the ion pair formed between the ions studied in ref 162 is significantly more stable than its covalent counterpart, a remarkable outcome for a hydrocarbon.^165^

Reactions between salts of the tropylium cation and the cyclopentadienyl anion usually form the covalent compound shown on the left of Figure 18a.^166^ In this case, the parent ions are not sterically hindered enough and do not have extensive charge delocalization, which is consistent with the formation of the covalent compound. However, the potential energy curve for 3^1^A′, shown in Figure 18a, suggests the formation of a hydrocarbon ion pair (containing two aromatic ions) in one of the excited states, as explained below.

**Figure 18:**
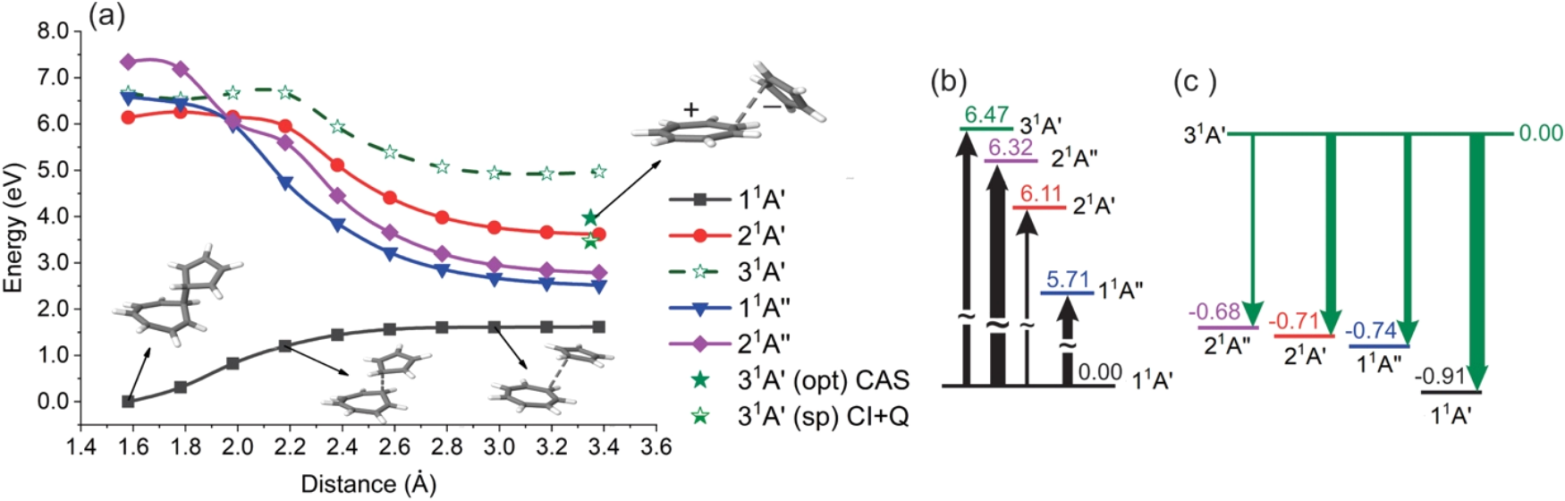
CASSCF/MR-CISD results relevant for the formation of the ion pair between cyclopentadienyl anion and tropyl cation from the covalent compound: (a) Potential energy curves generated from single-point calculations (at the CASSCF level) on the geometries obtained from a relaxed scan along the C–C distance (shown in the structures) between the two fragments. The structure on the far right corresponds to the optimized geometry for the 3A′ state. In both (b, c), the *f* and Δ*E*_v_ results are given. In (b, c), they have been computed at the optimized geometries of the ground state and ion-pair, respectively. Both sets of Δ*E*_v_ values (in eV) were calculated at the MR-CISD+Q level. The widths of the arrows represent relative oscillator strengths computed at the MR-CISD level.

The state-averaged CASSCF calculations performed with COLUMBUS provided a balanced description of several low-lying states important for the photodissociation of the C–C bond, as shown in Figure 18a. The potential energy curves shown in this plot were obtained from single-point calculations performed on the ground-state geometries generated from a relaxed scan (along the mentioned C–C bond) at the state-averaged CAS­(12,11) level with the 6–31+G* basis set. *C*_*s*_ symmetry was imposed, and five singlet states (3^1^A′ + 2^1^A″) were averaged with equal weights. At the optimized ground state geometry, the 11 active orbitals include a σ_CC_/σ_CC_* pair, five π and four π* orbitals, respectively. For larger distances, the σ_CC_/σ_CC_* pair turns into a π/π* pair, yielding six π and five π* orbitals, respectively.

Until ∼2.5 Å, some states show large multiconfigurational characters involving complex electronic configurations. Further details will be published elsewhere. For larger distances, the configurations of four states (1^1^A′, 2^1^A′, 1^1^A″, and 2^1^A″), due to the degeneracy of the SOMO orbitals of tropyl and cyclopentadienyl radicals, are consistent with dissociation yielding the mentioned ground state radicals. The fifth state (3^1^A′) yields the tropylium cation and the cyclopentadienyl anion, both aromatic ions. The optimization (at the CASSCF level) of this state leads to the ion pair structure shown on the right side of Figure 18a. It is clear from the potential energy curves that, starting from the Franck–Condon (FC) region, this structure can be generated through nuclear relaxation either directly from the 3^1^A′ state or via nonadiabatic transitions from other states.

Using the occupation numbers (n_occ_) of the active orbitals at the CASSCF level, it was possible to set up an MR-CISD calculation yielding ∼1.1 billion CSFs. The 6–31+G* basis set was adopted again. This calculation is based on a RAS + CAS + AUX scheme, where only single RAS → CAS and CAS → AUX excitations are allowed. The RAS and AUX orbitals were extracted from the initial set of 11 active orbitals, picking up the three with the largest and smallest values of *n*_occ_, respectively. The remaining five orbitals were kept in the active space. This MR-CISD calculation was performed at the optimized geometries of the ground state (Figure 18b) and ion-pair (Figure 18c). Figure 18b shows the vertical excitation energies (Δ*E*_v_) at the MR-CISD+Q level, along with arrows whose widths represent the relative oscillator strengths (*f*).

The largest and the smallest *f* values are 0.200 and 0.004, corresponding to the 1^1^A′ → 2^1^A″ and 1^1^A′ → 2^1^A′ transitions, respectively (see Figure 18b). Thus, the former transition can be crucial in yielding the mentioned ion pair, depending on the potential energy curves at the MR-CISD/MR-CISD+Q level, which will be presented in a forthcoming publication. At this same level, the ion-pair energy is 3.47 eV larger than that of the ground state, as shown on the right side of Figure 18a. Assuming that the 1^1^A′→2^1^A″ transition can lead to the ion pair, we note that its Δ*E*_v_ (6.32 eV, see Figure 18b) is slightly smaller than the approximate threshold energy of 6.49 eV required to generate the separated ions from the ground state structure. This value has been estimated by extrapolating the ground state potential energy curve to ∼2.0 eV (see Figure 18a) along with the adiabatic ionization energy of tropyl (6.28 eV)^167^ and electron affinity of cyclopentadienyl (1.79 eV)^168^ radicals. Similarly, the highest energy transition (1^1^A′→3^1^A′, see Figure 18b) is only 0.02 eV smaller than the mentioned threshold. Therefore, from an energetic point of view, all studied states in the FC region are capable of yielding the aromatic ion pair. However, as shown in Figure 18a, the profiles of the potential energy curves should also be taken into consideration. This point will be further discussed in a forthcoming publication.

Through comparison between Figure 18a and 18b it is clear that one has reordering of some states when dynamic electron correlation and extensivity correction are included. While at the CASSCF level the ordering is 2^1^A′ < 1^1^A″ < 3^1^A′ < 2^1^A″, at the MR-CISD+Q level it changes to 1^1^A″ < 2^1^A′ < 2^1^A″ < 3^1^A′. It is also clear that the main effect is on the states of A″ symmetry.

Assuming that the studied ion-pair is a minimum, a significant characteristic is its potential fluorescence. As shown in Figure 18c, it is a potentially fluorescent species in the near-infrared. The ion pair is also characterized by a significantly larger dipole moment of 15.47 D, compared to the dipole moment of 0.40 D in the ground state, both at the MR-CISD level. Its dipole moment is close to that of another hydrocarbon ion pair studied recently^164^ and considerably larger than those of other ion pairs also studied at the MR-CISD level using COLUMBUS.^169–173^

### 4.6 Uranium–Uranium Bond Analysis at the MRCI Level

The COLUMBUS program package provides the basis for performing chemical bond analysis at the MRCI level of theory by utilizing the MRCI one-electron density matrix and the natural orbitals obtained from it. Transition metal and f-element dimers are known to be challenging systems for electronic structure methods, often requiring correlated multireference calculations. We exemplify the bond analysis here with the example of the uranium dimer, U_2_, which has previously attracted much attention and some controversy as to whether the bond order (BO) is 4 or 5.^174–176^

The calculations for U_2_ were performed using the cc-pVTZ-DK3 ^177^ basis set. CASSCF, restricted active-space SCF (RASSCF), and integral calculations were performed with OpenMolcas,^22,178^ using the second-order Douglas–Kroll–Hess Hamiltonian for treating scalar relativistic effects. Spin–orbit effects were not included. Bond analyses were performed with natural bond order (NBO) algorithms^179^ using the NBO7 program^180^ with input data generated with COLUMBUS. To render the NBO input generation convenient, a utility program, molcasto47, was recently developed by some of us.^181^

The interatomic distance of 2.56 Å was taken from the literature, based on the relativistic calculations for U_2_ by Knecht et al.^176^ A 6-electron 20-orbitals (6,20) active space was constructed following the work of Gagliardi and Roos.^174^ The calculated occupations of the natural orbitals of the Ω = 8_g_ state (where Ω is the projection of the total angular momentum along the molecular axis) closely match the values reported in the literature,^174,175^ despite differences in the basis sets and bond parameters. Single-state CASSCF calculations identified the state with Ω = 8_g_ as the ground state, which was previously reported as one of the possible ground states.^174,175,182^ In subsequent RASSCF­(12,26) calculations, additional σ and π bonding and antibonding orbitals were included with double excitations. Note that the ground state is studied here without consideration of the SO interaction and corresponds to the state ^7^O_8_ with Λ= 11.

The MRCI calculations were based on the CASSCF-optimized orbitals. Here, 62 orbitals (including U shells up to 4s) were frozen, and a RAS­(6,15) reference space was generated with just single excitations allowed to orbitals with CASSCF occupations smaller than 0.484. The active-space orbitals and allowed excitations were selected based on the occupations of CASSCF natural orbitals (Figure 19) of the Ω = 8_g_ (B_3g_) state. The MRCI wave function included 22 configurations in the reference wave function following the same state symmetry and allowed active space excitations. The selected reference state construction was based on an analysis of the CASSCF occupation numbers and set up with the intent to minimize the number of reference states and maximize the effects of dynamic correlation. All external double excitations conserving the state symmetry were allowed in the CI step for each reference state configuration; the total number of configurations in the MRCI calculation was approximately 100 million.

**Figure 19:**
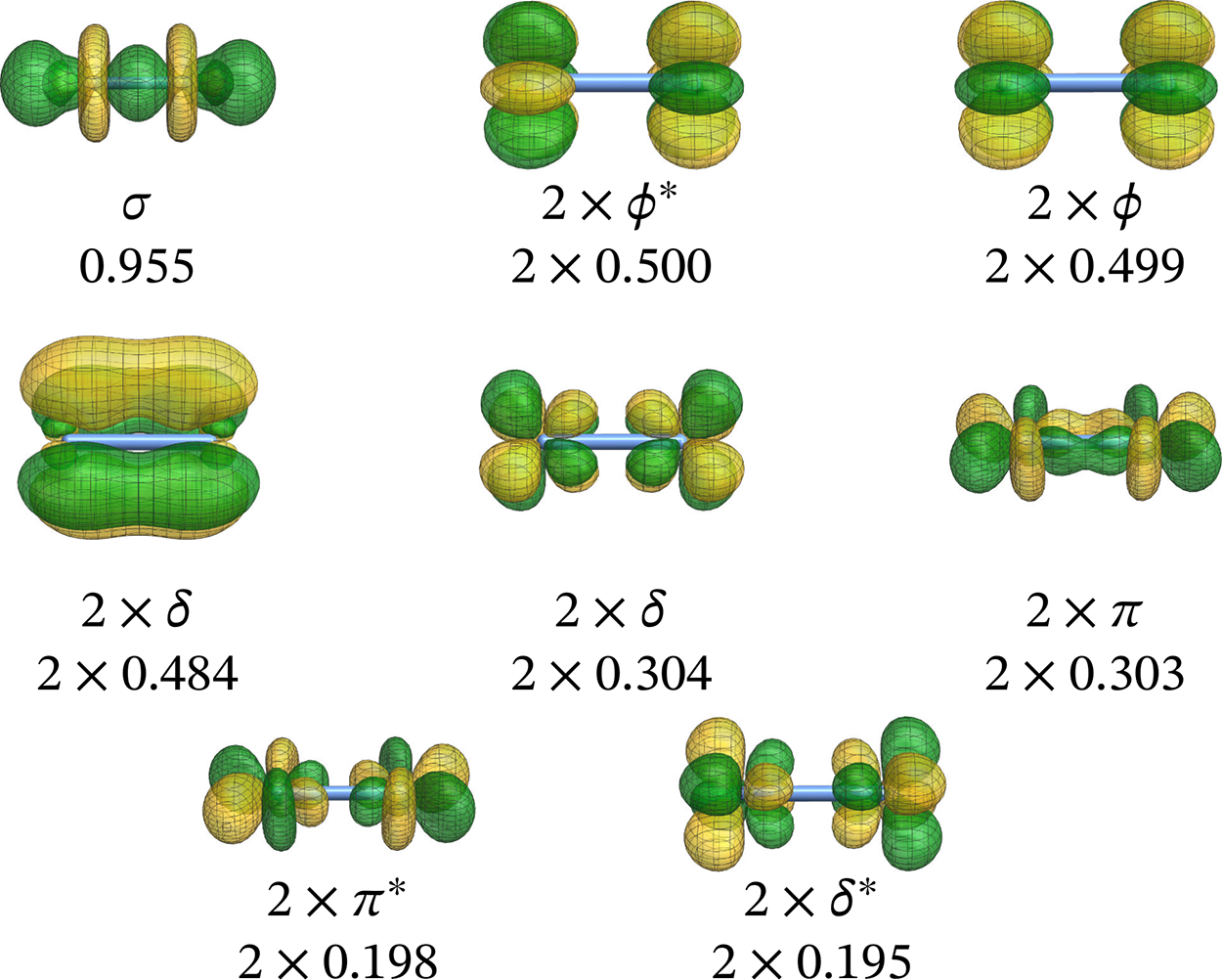
Selected active space orbitals (±0.04 isosurfaces) and occupations from CASSCF­(6,20) included in the construction of the reference states of U_2_ for the MRCI calculation of the Ω = 8_g_ state.

The effective bond order (EBO) definition used by Roos and Gagliardi^174^ to assign U_2_ a quintuple bond utilizes the typically noninteger occupation numbers obtained from multiconfigurational calculations. However, it does not explicitly consider the overlap of the participating atomic orbitals (AOs). The bonding and antibonding character of an orbital is qualitatively assessed to obtain the EBO, which is then defined as half of the sum of occupations of bonding valence MOs minus half of the sum of occupations of antibonding MOs. Based on the CASSCF occupations, the EBO is 4.14, similar to the value of 4.2 reported by Roos and Gagliardi. This result is assigned a quintuple bond character, considering that the bond order is above 4. Other numerical bond indices obtained in the present work from the 1-electron reduced density matrix and the AO overlap are collected in Table 3.

**Table 3:** U_2_ Bond Orders Calculated with Different Wave Functions and Bond Order Criteria

method	CASSCF(6,20)	RASSCF(12,26)	MRCI
MBO	3.44	3.05	3.42
WBI	3.34	2.97	3.30
NLMO	2.01	4.01	4.01

The Wiberg bond index (WBI)^183^ and Mayer bond order (MBO)^184^ vary according to the level at which the dynamic correlation is treated and as a function of the active space. The WBI is defined for orthonormal orbitals, such as a set of Löwdin-orthonormalized atomic orbitals (AOs) or the natural atomic orbitals (NAOs) generated in NBO runs. Therefore, a measure of the AO overlap is implicitly included in the WBI. The MBO explicitly considers the AO overlap. We also report bond orders based on the natural localized molecular orbital (NLMO) population analysis generated in NBO runs; however, the results obtained for U_2_ vary strongly and may therefore be unreliable. Note that bond indices that can be generated from the overlap and density matrices do not require a subjective assessment of whether a given MO is bonding or antibonding or whether it should be excluded from the BO estimation. They are, thus, arguably a more objective measure for bond order than EBO. As seen in Table 3, the WBI and MBO are similar for U_2_ and considerably smaller than the EBO, indicating a BO of at most 4 for U_2_. These findings agree with the BO assignment of U_2_ by Knecht et al.^176^

A critical factor in the assignment of the U_2_ BO is the bonding furnished by the 5f AOs of ϕ symmetry. As shown in Figure 20, even with the relatively small isosurface values used for the plots, the resulting ϕ-symmetric linear combinations are identified as barely bonding and only weakly antibonding, respectively. The MRCI orbitals appear similar to the ϕ orbitals of the active space. Their contributions to the covalent bonding in the dimer are, therefore, comparatively weak. Based on the occupations obtained from the different calculations (Table 4), the contributions to the EBO are also small due to the nearly equal occupations of the bonding orbitals and their antibonding counterparts. However, the overall ϕ orbital populations are enhanced with MRCI, indicating that correlation outside the reference space influences the wave functions.

**Figure 20:**
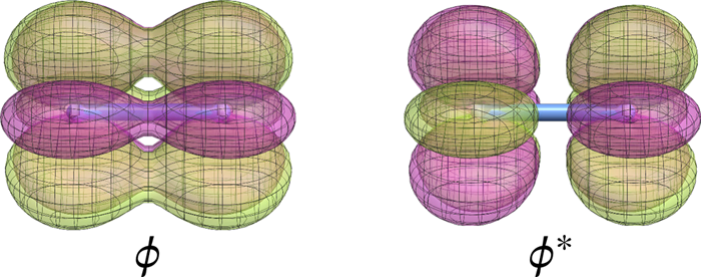
Bonding and antibonding ϕ-symmetry natural localized molecular orbitals (from MRCI, ±0.01 isosurfaces).

**Table 4:** U_2_ Occupations of ϕ and ϕ* Symmetry Natural Localized Molecular Orbitals

method	ϕ	ϕ*
CASSCF(6,20)	0.499	0.499
RASSCF(12,26)	0.496	0.494
MRCI	0.766	0.729

Overall, the present MRCI results indicate that the inclusion of dynamic correlation does not dramatically impact the resulting bond order of U_2_ compared to what is obtained from multiconfigurational wave function calculations with carefully selected active spaces. However, the correlated MRCI results can be regarded as less active-space dependent and, therefore, somewhat more reliable because they include variationally calculated orbital populations across all orbitals beyond the frozen core–shells. More generally, therefore, MRCI is useful in this type of study, as it provides energies and wave functions at a consistently high level of theory, which is not possible for perturbative correlation methods. A more detailed analysis of the reference space structure and static correlations as they influence the numerical bond orders as well as the role of higher excitations is left for subsequent studies.

## 5 Applications: Excitation Energies and Potential Energy Surfaces

### 5.1 Consistent Treatment of Covalent and Ionic States of Hexatriene at MR-CISD Level

The study of all-*trans* polyene chains has a long-standing tradition due to their distinctive spectral properties, which arise from the structure of their low-lying excited-state manifold.^51^ In small- and medium-sized polyenes, the two low-lying excited states are the covalent ^1^A_g_^–^ and the ionic ^1^B_u_^+^ state. The wave functions of the covalent states are intricate, with significant contributions from doubly excited configurations, whereas those of the ionic states are predominantly composed of singly excited configurations.^185^ This imposes significant challenges in accurately and uniformly describing both states.^185^ MR-CISD and MR-AQCC calculations have previously been performed for ethylene^186^ and butadiene,^187^ the first members of this series. Here, we report an accurate and balanced treatment of both static and dynamic electron correlation effects in *trans* hexatriene using the variational MR-CISD and MR-AQCC methods, which are available in COLUMBUS. Our findings led us to extend our investigation to the electronic spectra of larger members of the all-*trans* polyene series.^188^

A set of CASSCF molecular orbitals derived from a standard π-valence complete active space (CAS) with six electrons in six orbitals, CAS­(6,6), was employed for the MR-CISD and MR-AQCC calculations. For these computations, the reference active space was constructed using the same π-valence CAS, and only the core orbitals were frozen. Vertical excitation energies from the ground state to the excited states ^1^B_u_^+^ and ^1^A_g_^–^ were calculated using the cc-pVnZ (n = D, T, Q) basis sets.^60^ Extrapolation to the complete basis set (CBS) limit was performed using a two-point fit approach.^189^ For the MR-CISD calculations, the Pople (+P) nonvariational size-extensivity correction was applied.^18^

Three main factors significantly influence the description of the two lowest excited states. These factors vary in magnitude depending on whether the state has covalent or ionic character. The first factor is the inclusion of dynamic σ-π electron correlation. At the CASSCF level, which primarily captures static correlation effects, the covalent state is well described, with no significant difference observed between the CASSCF and MR-CISD or MR-CISD+P results (Figure 21). In contrast, the vertical excitation energy to the ionic state is strongly overestimated by 1.09 eV compared to the MR-CISD results. This overestimation increases to 1.81 eV when compared to the size-extensivity corrected MR-CISD+P result (Figure 21).

**Figure 21:**
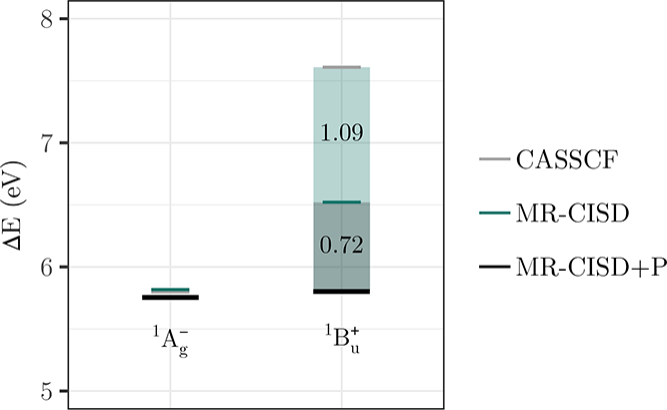
CASSCF, MR-CISD, and MR-CISD+P vertical excitation energies (in eV) for the ^1^A_g_^–^ and ^1^B_u_^+^ states of hexatriene. The results were obtained using the cc-pVDZ basis set. The numerical difference between CASSCF and MR-CISD, as well as between MR-CISD and MR-CISD+P, are indicated for the ^1^B_u_^+^ state.

This discrepancy between the MR-CISD and MR-CISD+P results highlights the second factor influencing the description of the excited states: the size-extensivity error. While this error has a negligible impact on the covalent state, applying the size-extensivity correction stabilizes the ionic state by 0.72 eV (Figure 21). Notably, the MR-CISD+P results, corrected for size-extensivity, show good agreement with those obtained at the MR-AQCC level (Table 5).

**Table 5:** MR-CISD+P and MR-AQCC Vertical Singlet Excitation Energies (in eV) of Hexatriene^a^

	MR-CISD+P			MR-AQCC				
state	Δ*E*_D_	Δ*E*_DT_^∞^	Δ*E*_TQ_^∞^	Δ*E*_D_	Δ*E*_DT_^∞^	Δ*E*_TQ_^∞^	TBE^b^	CASPT2
^1^A_g_^–^	5.755	5.690	5.710	5.744	5.659	5.658	5.62	5.57^c^
^1^B_u_^+^	5.803	5.613	5.568	5.764	5.495	5.343	5.37	5.31^d^

a Values computed using the cc-pVDZ basis set (Δ*E*_D_), as well as values extrapolated from double-zeta to triple-zeta (Δ*E*_DT_^∞^), and from triple-zeta to quadruple-zeta (Δ*E*_TQ_^∞^), are presented. Theoretical reference values are provided for comparison.

b Theoretical best estimates (TBE) from ref 41.

c Reference 190.

d Reference 191.

The third factor is the basis set. For the covalent state, when comparing nonextrapolated excitation energies obtained using the cc-pVDZ basis set (Δ*E*_D_) with values extrapolated from double-ζ to triple-ζ (Δ*E*_DT_^∞^), a stabilization of 0.06 eV is observed at the MR-CISD+P level (Table 5), increasing to 0.08 eV at the MR-AQCC level. For the ionic state, the stabilization amounts to 0.19 eV at the MR-CISD+P level and 0.27 eV at the MR-AQCC level (Table 5). Comparing Δ*E*_DT_^∞^ with excitation energies extrapolated from triple-ζ to quadruple-ζ (Δ*E*_TQ_^∞^), no significant difference is observed at the MR-CISD+P level for either state. However, at the MR-AQCC level, the ionic state undergoes further stabilization by 0.15 eV (Table 5). Notably, the extrapolated MR-AQCC results demonstrate slightly better numerical agreement with theoretical reference values.

Examination of the transition density (Figure 22) reveals that for the covalent state, the transition charges are centered along the bonds. In contrast, for the ionic state, they are localized on the atoms.^17,39^ Moreover, the transition density of the ionic state at the MR-CISD level shows distinct σ-contributions as in-plane rings around the out-of-plane lobes that are absent in the CASSCF transition density. These σ-contributions are associated with the reduction of transition density self-repulsion, which is generally significant when there is considerable spatial overlap between the involved orbitals.^44^ The studies by Kimber and Plasser^43,44^ on the role of transition density self-repulsion demonstrate that while the self-repulsion term raises the excitation energy, it can be compensated by σσ* excitations. Consequently, the inclusion of σ-contributions in the wave function reduces the energetic penalty of the pure HOMO–LUMO transition, thereby lowering the excitation energy and resulting in reduced oscillator strengths.^4,192^

**Figure 22:**
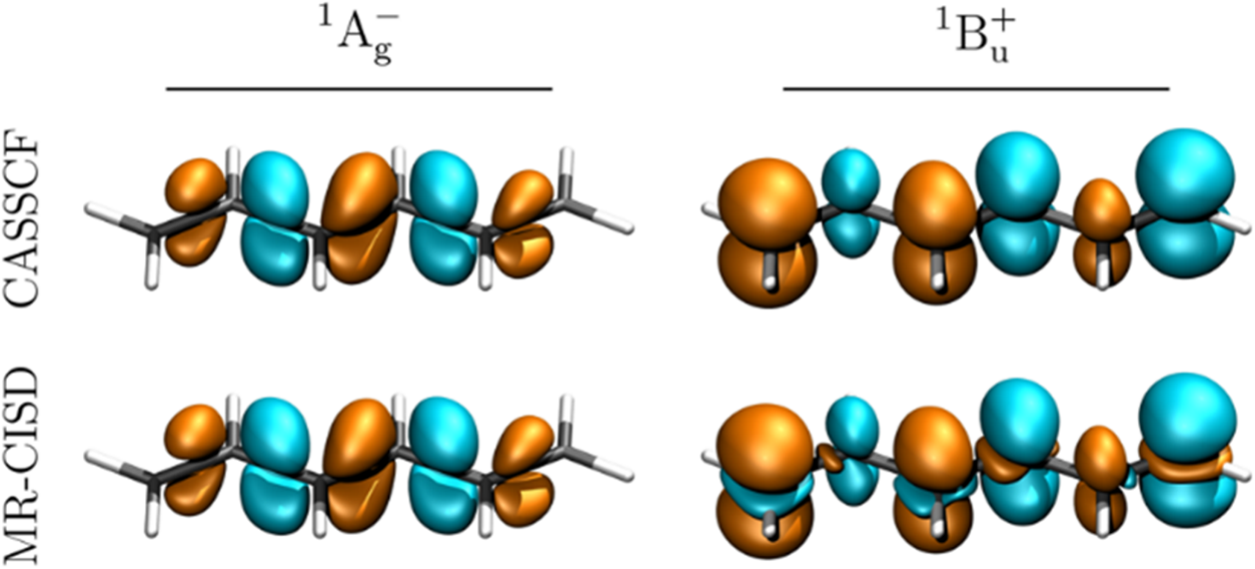
Transition densities (isovalue: 0.002 au) between the ground state and the excited states ^1^A_g_^–^ and ^1^B_u_^+^ of hexatriene, computed at the CASSCF and MR-CISD levels of theory using the cc-pVDZ basis set.

In summary, while covalent states are well described by MCSCF theory, an accurate description of the ionic state requires extensive treatment of σ-π electron correlation, the inclusion of size-extensivity corrections, and the use of a sufficiently large basis set. Accounting for these factors, the MR-CISD and MR-AQCC results consistently indicate that the ionic ^1^B_u_^+^ state is the first vertically excited state in *trans*-hexatriene. Based on the Δ*E*_TQ_^∞^ extrapolated results, this state lies 0.14 eV below the covalent state according to the MR-CISD+P calculations and 0.32 eV below according to the MR-AQCC calculations.

### 5.2 Charge-Transfer States in Doped Periacenes

The generation of charge transfer (CT) states at donor/acceptor (D/A) interfaces is a crucial process that initiates hole/electron separation in photovoltaic and photocatalytic processes.^193,194^ PAHs have gained significant interest as basic semiconductor materials due to their unique electronic band gap properties, which enable convenient tuning through doping. Doping with heteroatoms appears to be a promising way to provide flexible tuning possibilities. It has been shown previously^195–199^ that especially N or B,N doping is efficient in controlling the biradicaloid character of PAHs. In this work, borazine rings inserted into a (5a,5z) periacene sheet were used to function as a D/A interface. From several insertion possibilities investigated, the B,N-center, and B,N-vertical cases, as displayed in Figure 23, will be discussed in this contribution, as they exemplify well the creation of the CT states. Calculations on PAHs are challenging since they acquire open-shell character with an increasing number of benzene rings.^11,200,201^ B,N doping is supposed to stabilize the PAH sheets, but the doped systems will possess a polyradical character as well.^202^ Therefore, the use of multireference (MR) calculations is crucial to obtain reliable results for ground and excited state properties.

**Figure 23:**
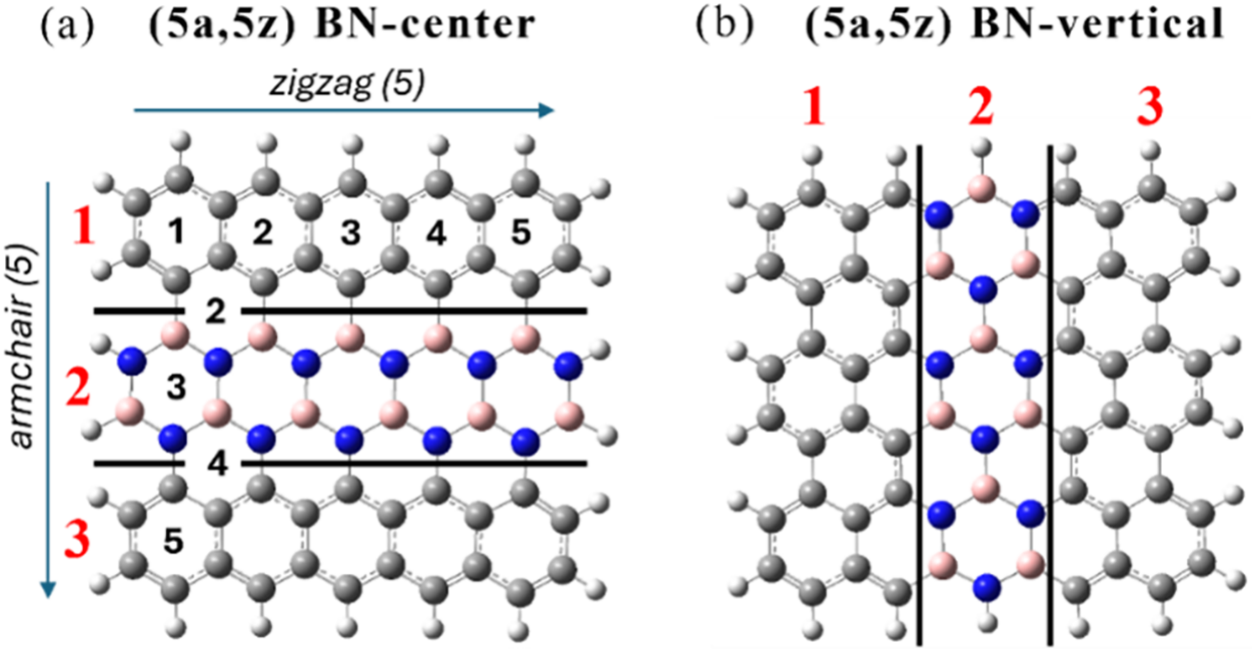
(a) B,N-center and (b) B,N-vertical inserted borazine chains in a (5a,5z)-periacene. The boundaries of the borazine row are indicated by the black lines, which also define the segment numbers, marked by the red numbers.

The present contribution employs MR-CISD.^50,51^ Energies include the *a posteriori* Pople size extensivity correction,^18^ which is denoted as MR-CISD+P. Molecular orbitals used in the MR-CISD calculation are derived from a state-averaged (SA)-MCSCF calculation. The reference space of the MR-CISD and the active space of the MCSCF calculation use the complete active space (CAS) of eight electrons in eight orbitals CAS­(8,8). The eight π orbitals, corresponding to the b_1_ and a_2_ irreducible representations, were chosen according to natural orbital (NO) occupations. In each of the two irreducible representations, the two highest occupied (HONOs) and the two lowest unoccupied (weakly occupied) NOs (LUNOs) were chosen. The choice of this reference space has been made based on the weight of the reference configurations in the wave function expansion (nonreference configurations should have a weight of less than approximately 2%), on NO occupations, and experience obtained with previous calculations on the typical biradical compound heptazethrene.^147^ For reasons of computational efficiency, all σ orbitals were frozen. It has also been previously shown that for the calculation of energy differences between singlet/triplet or different spin multiplicities for phenalenyl, freezing all σ orbitals influences the results by ∼0.1 eV only.^100,147^ Moreover, the 6–31G basis set^148^ was used following the work reported in ref 147 where differences of <0.1 eV were found for excited states of phenalenyl in comparison to larger, polarized basis sets. The wave function analysis was carried out through TheoDORE using the one-particle transition density matrices.^40,203^ More details can be found in ref 204.

Figure 24 presents the energy diagram of the four lowest excited states for the BN-center and BN-vertical doped periacene structures, classifying them as locally excited (LE) or charge-transfer (CT) states. The excitations are decomposed among the three molecular fragments defined in Figure 23. In the case of the BN-center structure, the bright state is *S*_2_, which is dominated by a “3–3” type state, characterized by a locally excited configuration on the bottom pentacene. *S*_1_ exhibits a significant “3–1” type CT character, meaning an excitation from the bottom pentacene to the top pentacene unit. *S*_3_ is a state completely dominated by double excitations (as defined in ref 205). It is, therefore, not accessible by single-excitation methods such as the popular TDDFT method or the second-order algebraic construction method (ADC(2)). It is located above the bright state but is quite close in energy, so one might want to consider it as well. The BN-vertical structure describes an even more critical situation concerning the doubly excited state: it is *S*_1_ lying well below 1 eV and, therefore, all other states. The bright state is *S*_3_. Reviewing Figure 24, we note that aside from the S_3_ and S_1_ excited states discussed above, the different states also have a significant admixture of doubly excited character (indicated by light yellow bars representing the Ω-diagnostic^205^), highlighting the challenges of accessing any of these states without a multireference framework.

**Figure 24:**
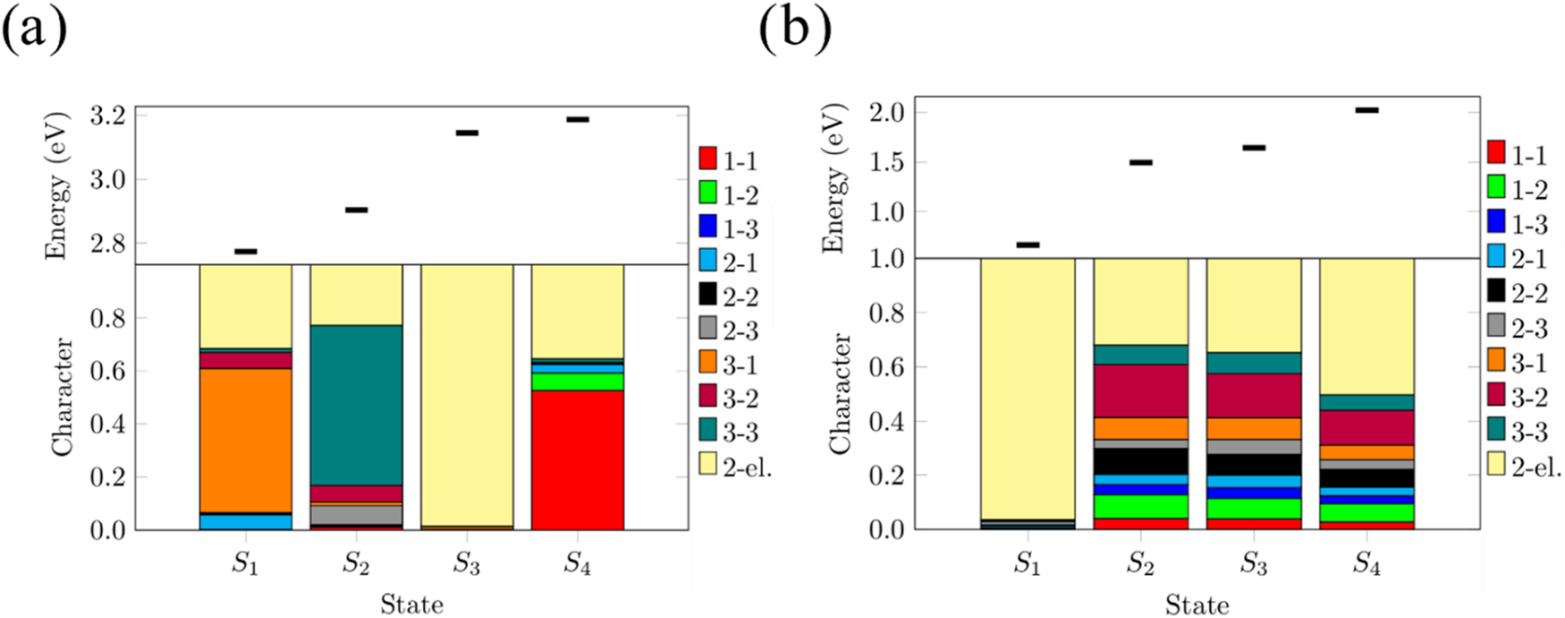
Excitation energies (eV) and analysis of the character of the electronic transitions for (a) the BN-center and (b) BN-vertical structures. The state composition bar plot is numbered in terms of the three adopted segments (Figure 23), where the first number designates the hole and the second one the electron.

Natural transition orbitals (NTOs) for the states dominated by single excitations and electron density differences for the doubly excited states are shown in Figure 25 for selected transitions. The *S*_1_ state of the BN-center structure represents a conventional CT state, which is separated by the borazine interface (as discussed above). It should be well-suited for subsequent charge separation dynamics. Interestingly, the two mentioned doubly excited states exhibit a similar CT character, where the borazine chain also serves as a boundary.

**Figure 25:**
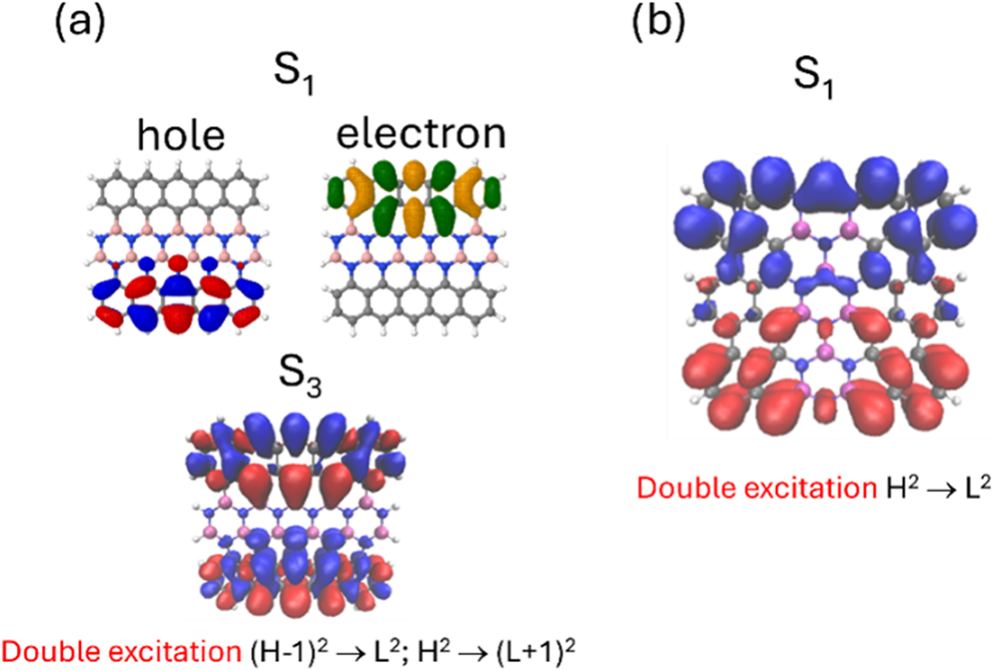
NTOs showing hole and electron orbitals and density difference plots between the excited and ground states for (a) the BN-center and (b) the BN-vertical structure. For the density difference plots, the red color indicates a decrease in electron density, and blue indicates an increase in density. H stands for highest occupied MO (HOMO) and L for lowest unoccupied MO (LUMO).

Both BN-center and BN-vertical structures present interesting CT cases and potentially could be used as photocatalysts in oxidation/reduction processes, given the separation of the electron/hole pair. We note that, especially for the BN-vertical doped structure, consideration of double excitations is essential. Therefore, this work demonstrates the need for high-level computational methods to accurately understand the electronic states of these doped periacene structures.

### 5.3 Spin–Orbit CI on the Electronic Structure of the CUO Molecule

The Spin–Orbit Configuration Interaction (SO–CI) formulated with GUGA^13^ represents a robust computational methodology in quantum chemistry, particularly for systems where both electron correlation and spin–orbit coupling play a significant role. In the COLUMBUS GUGA SO–CI, the spin–orbit coupling terms are incorporated directly into the CI Hamiltonian, allowing for the simultaneous treatment of electron correlation and relativistic spin–orbit effects. The COLUMBUS programs initially implemented the relativistic effective core potentials^206^ and the associated spin–orbit operators.^207^ Subsequently, the implementation was adapted to work with all-electron relativistic Hamiltonians, using mean-field spin–orbit integrals as available in OpenMolcas.^14,24,208^ Furthermore, the spin–orbit operators and integrals implemented in the COLUMBUS programs, as well as the spin–orbit configuration interaction, can also be used in the implementation of quantum computing for Hamiltonians with spin–orbit interactions, for example, refs 209,210 .

Spin–orbit coupling is crucial for describing heavy-element systems, where these effects have a significant impact on spectroscopic and chemical properties. The GUGA CI with spin–orbit interaction provides a robust framework for understanding complex phenomena, such as fine-structure splitting, spin-forbidden transitions, and the electronic structure of systems with significant relativistic contributions. A wide range of chemically and spectroscopically relevant systems has been studied.^211–215^

In this work, we review the application of the GUGA CI with spin–orbit interactions to the CUO molecule and illustrate the approach’s power for accurate calculations, as well as its capability to resolve discrepancies in the extensive experimental and theoretical work. The spectroscopic properties of the molecule CUO have been extensively studied to establish the structural properties and low-lying electronic states of the CUO molecule. A study of the infrared spectra of CUO in rare-gas matrices revealed that the molecule is linear and that the two stretching frequencies differed by unusual amounts when the host gas was changed from neon (Ne) to argon (Ar).^216,217^ The study established the interaction of inert gases with the polar molecules containing uranium atoms. Extensive calculations have been carried out to understand the electronic structures of the ground and low-lying electronic states, as well as the weak interaction with the noble gas matrix, employing a range of methods from DFT calculations to sophisticated coupled cluster calculations while treating spin–orbit effects in various approximations (see ref 215 and references therein). However, it is only with the spin–orbit interaction GUGA CI approach that the correct order of the low-lying electronic states is established, indicating that large-scale configuration interactions with correlation and spin–orbit effects treated on the same footing are necessary for an accurate determination of the electronic structures in such cases.

Figure 26 shows the low-lying orbitals and electronic states, as well as the CU and OU stretch frequencies in the low-lying electronic states. Experimentally, in Ne matrices, the infrared spectra indicate that the closed shell state with the σ^2^ electronic configuration is the ground state. However, in the Ar matrix, the order of the low-lying electronic states changed due to the larger interaction of Ar with the open shell triplet states with electronic configuration σ^1^φ^1^. DFT, CASMP2, and CCSD­(T) calculations all place the triplet state as the ground state in the absence of spin–orbit interactions and position the triplet state further below the closed-shell state when spin–orbit interactions are considered. The effect of the Ar interaction is estimated to be 0.05 eV; thus, those calculations failed to explain the observed reversal of the Ar interaction’s effect on the order of low-lying electronic structures compared with that in the Ne matrix. Our extensive spin–orbit CI calculations show that when spin–orbit interaction and correlation effects are considered simultaneously, the ground state of CUO is calculated to be the singlet state of the closed-shell configuration, ^1^∑, Ω = 0. The lowest electronic state from the open-shell triplet configuration is calculated to be the Ω = 2 state, which is predominantly ^3^Δ of the σ^1^δ^1^ configuration. It is only ca. 0.047 eV above the calculated ground state energy. Our results are consistent with the experimental observation that the closed-shell singlet state is the ground state observed in the Ne matrix. The stronger interaction with the Ar matrix, estimated to be 0.005 eV, can lower the triplet configuration state to become the ground state in the Ar matrix.

**Figure 26:**
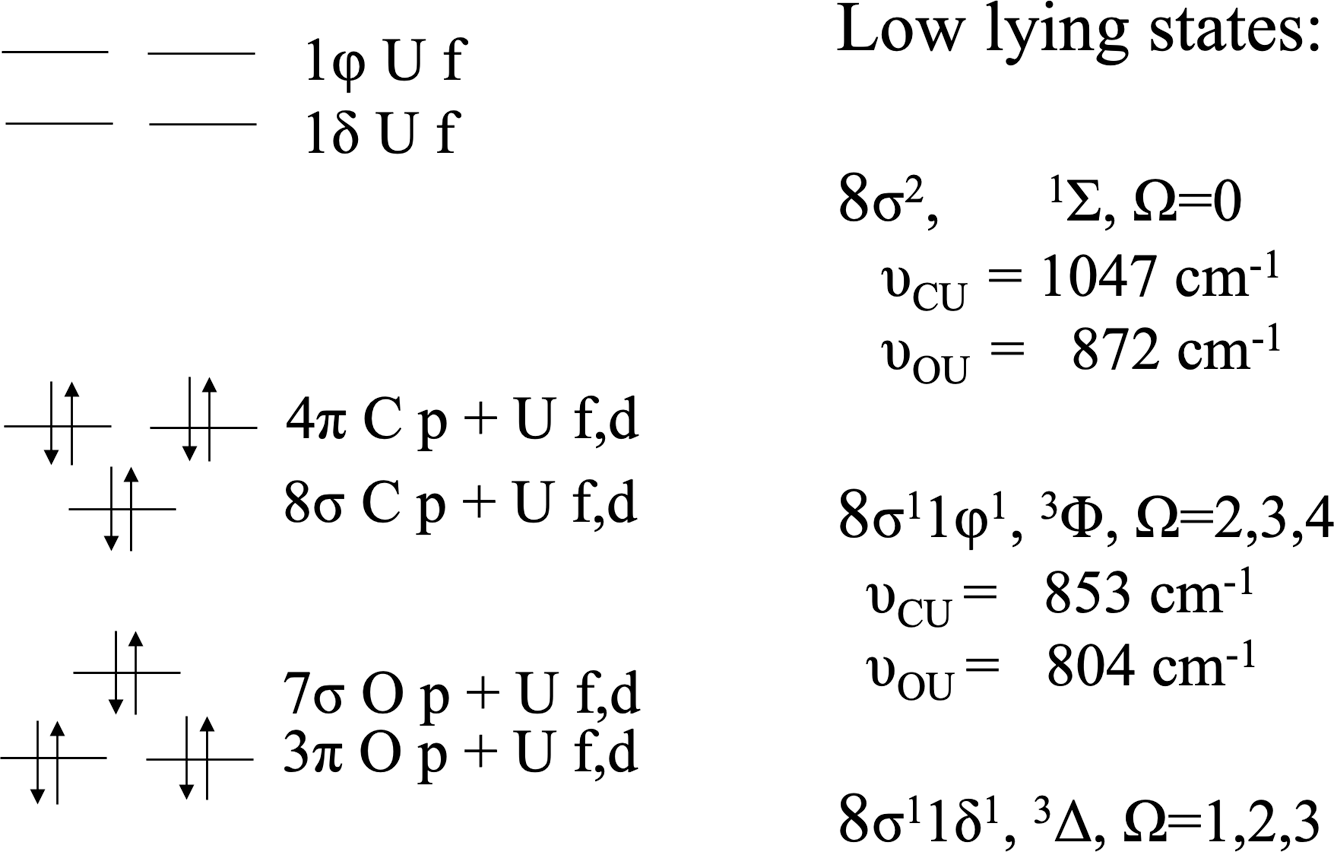
Electronic structure of CUO. The CU and OU vibrational frequencies are the experimental measurements of electronic ground states. In the Ne matrix, when the interaction with the inert gas is minimal, the closed-shell state is the ground state. In the Ar matrix, the open-shell polarized electronic state becomes the lower-lying electronic state due to the strong interaction of Ar with the polarized state. The pictures shown here depict the electronic configurations at the SCF level of calculations.

## 6 Applications: Photodynamics

### 6.1 Nonadiabatic Molecular Dynamics with the COLUMBUS/Newton-X Interface

COLUMBUS has often been used in nonadiabatic molecular dynamics simulations.^218–220^ Its analytical gradients and nonadiabatic couplings are invaluable for this type of application. Indeed, when the trajectory surface hopping platform Newton-X was developed, its first interface was with COLUMBUS.^217^ It provided information on electronic structures, such as potential energies, energy gradients, and nonadiabatic couplings for dynamics. COLUMBUS still plays a crucial role in Newton-X, which has recently extended its capabilities to run ab initio multiple spawning (AIMS) using the methods available in COLUMBUS.^218^

Trajectory surface hopping propagates a swarm of independent classical trajectories.^221^ At each integration time step, a trajectory lies on a single Born–Oppenheimer potential energy surface of one of the molecular electronic states. To emulate the nonadiabaticity of the nuclear wavepacket (i.e., the population transfer between states), surface hopping estimates transition probabilities between pairs of states and stochastically allows the classical trajectories to switch to another state. Thus, the ensemble of independent trajectories forms a nonadiabatic classical wavepacket by capturing branching events.

The propagation of independent trajectories and the need for electronic structure information exclusively at classical nuclear geometries make surface hopping suitable for on-the-fly (or direct) propagation,^222^ where the electronic structure is computed during the dynamics instead of being stored as a precomputed multidimensional potential energy surface.

Nonadiabatic dynamics with COLUMBUS can be directly run using its native nonadiabatic coupling vectors, time-derivative couplings based on wave function overlaps,^223,224^ or still curvature-based time-derivative couplings using the time-dependent Baeck-An approach.^225^ It can also be done on complex-valued potential energy surfaces.^226^

One feature distinguishing COLUMBUS from most other multiconfigurational quantum chemistry programs is its outstanding flexibility in building customized wave functions. Thus, when faced with the challenge of describing excitations and processes that would require too large active orbital spaces, such as dissociation or bond formation, COLUMBUS can provide ways to reduce this description by any creative arrangement of subspaces and electron transitions between them.

This flexibility is illustrated in the surface hopping dynamics of ethylene reported in ref 219 in that study, the ultrafast dynamics of the ππ*-excited ethylene were simulated, including Rydberg states and the possibility of dissociation. Accounting for those effects required a complete active space of 12 electrons in 16 orbitals, which is too computationally expensive for on-the-fly evaluations during surface hopping. Nevertheless, with COLUMBUS, the following combination of subspaces was built at the MCSCF level

34 $$\begin{array}{l} \mathrm{MCSCF}=\left\{\left[{\mathrm{PP}}_{\mathrm{CC}}\right]\times 4\left[{\mathrm{PP}}_{\mathrm{CH}}\right]\times \left[\mathrm{CAS}{\left(2,2\right)}_{\pi }\to 4{\mathrm{AUX}}_{\mathrm{Ryd}}\right]\right\}\\ +\left\{\left[{\mathrm{PP}}_{\mathrm{CC}}\right]\times 3\left[{\mathrm{PP}}_{\mathrm{CH}}\right]\times \left[\mathrm{CAS}{\left(2,2\right)}_{\pi }↔{\mathrm{PP}}_{\mathrm{CH}1}\right]\right\}\\ +\left\{\left[{\mathrm{PP}}_{\mathrm{CC}}\right]\times 3\left[{\mathrm{PP}}_{\mathrm{CH}}\right]\times \left[\mathrm{CAS}{\left(2,2\right)}_{\pi }↔{\mathrm{PP}}_{\mathrm{CH}2}\right]\right\}\\ +\left\{\left[{\mathrm{PP}}_{\mathrm{CC}}\right]\times 3\left[{\mathrm{PP}}_{\mathrm{CH}}\right]\times \left[\mathrm{CAS}{\left(2,2\right)}_{\pi }↔{\mathrm{PP}}_{\mathrm{CH}3}\right]\right\}\\ +\left\{\left[{\mathrm{PP}}_{\mathrm{CC}}\right]\times 3\left[{\mathrm{PP}}_{\mathrm{CH}}\right]\times \left[\mathrm{CAS}{\left(2,2\right)}_{\pi }↔{\mathrm{PP}}_{\mathrm{CH}4}\right]\right\} \end{array}$$

In this partition, PP_AB_ consists of a subspace containing two orbitals (σ_AB_ and σ_AB_*) and two electrons each, where only the two doubly occupied configurations are allowed. The wave function also allowed single excitations from the CAS­(2,2) ππ* space into four Rydberg orbitals. Finally, single and double substitutions from the CAS­(2,2) into any PP_AB_ space (and vice versa) were included.

Using this MCSCF wave function, MR-CISD was computed, including CSFs arising from single and double excitations from the following reference space

35 $${\mathrm{MRCI}}_{\mathrm{ref}}=\left[\mathrm{CAS}{\left(2,2\right)}_{\pi }\to 4{\mathrm{AUX}}_{\mathrm{Ryd}}\right]+\left[\mathrm{CAS}{\left(4,4\right)}_{\pi \sigma \left(\mathrm{CH}1\right)}\right]+\left[\mathrm{CAS}{\left(4,4\right)}_{\pi \sigma \left(\mathrm{CH}2\right)}\right]+\left[\mathrm{CAS}{\left(4,4\right)}_{\pi \sigma \left(\mathrm{CH}3\right)}\right]+\left[\mathrm{CAS}{\left(4,4\right)}_{\pi \sigma \left(\mathrm{CH}4\right)}\right]$$

More recently, COLUMBUS’ wave function flexibility was explored to describe the nonadiabatic dynamics of cyclobutanone excited into a Rydberg state, which could induce diverse types of dissociation.^220^ In this case, the MCSCF wave function was chosen as

36 $$\mathrm{MCSCF}=4\left[{\mathrm{PP}}_{\mathrm{CC}}\right]\times \left[{\mathrm{PP}}_{\mathrm{CO}}\right]\times \left[\mathrm{CAS}{\left(4,4\right)}_{n,\pi ,\pi *,3s}\right]$$

with the molecular orbitals shown in Figure 27.

**Figure 27:**
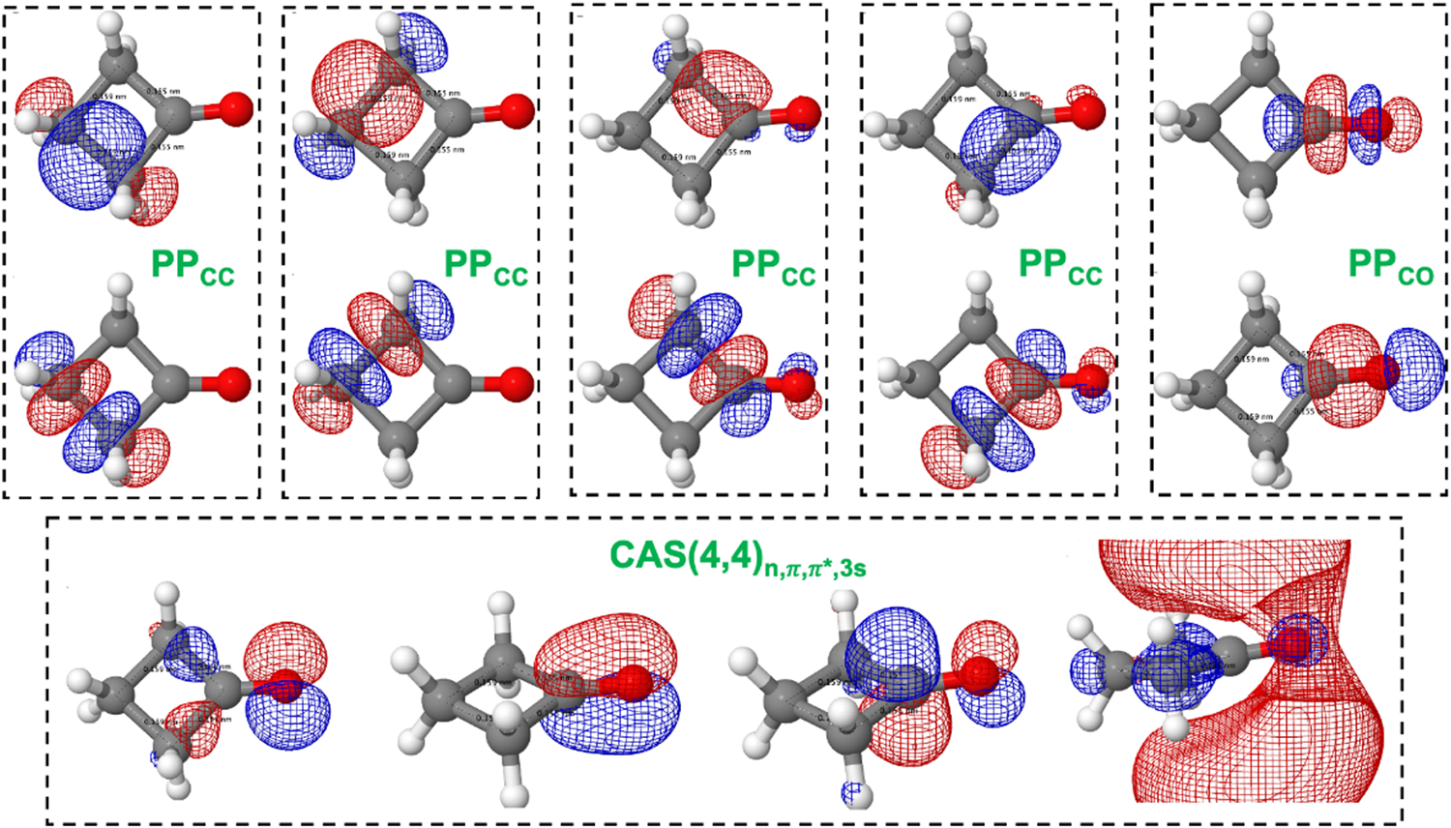
Molecular orbitals in the generalized active space used in the surface hopping of cyclobutanone. Reproduced with permission from ref 220. Copyright 2024 American Institute of Physics.

The construction of such specialized wave functions requires a high level of conceptual and technical expertise. Nevertheless, it pays off in terms of the superior quality of the results, with the flexibility to describe the photochemical process at relatively low costs, which cannot be achieved with standard active-space procedures.

Beyond the direct use of COLUMBUS in nonadiabatic dynamics, the advent of machine learning (ML) techniques for computational chemistry has opened other potential usages for the program. In this case, COLUMBUS is employed as a quantum chemistry program to build high-level data sets for training machine learning potentials. Such a novel application is illustrated in ref 227, where COLUMBUS was used during active learning of an ML potential for fulvene. The converged ML potential required about 6000 single-point evaluations of energies and energy gradients at the CASSCF level.

The advantage of such a procedure is that it significantly reduces computational costs. In the fulvene case, the computational cost due to the 6000 COLUMBUS calculations is equivalent to running only ten surface hopping trajectories. Nevertheless, after converging the ML potential, we can run thousands of new trajectories at a minimal cost.

### 6.2 Nonadiabatic Dynamics Using Machine Learning in SHARC

One of the first ML studies aimed at accelerating nonadiabatic surface hopping molecular dynamics utilized a reference data set derived from MR-CISD calculations performed with the COLUMBUS software package.^228^ The exceptional accuracy of electronically excited states offered by MR-CISD, combined with the ability to calculate nonadiabatic coupling vectors, made COLUMBUS an ideal tool for this application.

The goal here was to facilitate the interpolation of quantum chemical properties at significantly lower computational costs while maintaining the accuracy of advanced quantum chemical methods. To achieve this, an analytical function must be created that maps input variables, usually molecular geometry (X), to output properties (Y), such as potential energy surfaces, gradients, dipole moment vectors, and nonadiabatic coupling vectors. Unlike traditional physical models, which rely on explicit assumptions about the system, machine learning models utilize universal approximators that can represent any function with arbitrary accuracy, provided sufficient training data and model complexity are available. Consequently, ML proves to be highly effective when integrated into surface hopping molecular dynamics, provided there is suitable training data and mechanisms to monitor the reliability of the ML-generated potentials during simulations.^229–231^ These principles have been integrated into the SHARC program (surface hopping including arbitrary couplings),^232–234^ which pioneered the propagation of trajectories concurrently driven by nonadiabatic couplings and spin–orbit couplings.^235^

The SHARC method interfaces with COLUMBUS, as well as with many other quantum chemistry programs, which can provide energies, gradients, and ideally nonadiabatic couplings and spin–orbit couplings. In the absence of the latter, SHARC will evaluate these numerically, thereby uniquely positioning itself to handle internal conversion and intersystem crossing on an equal footing. Moreover, SHARC can be integrated with neural networks, such as SchNarc,^236^ which is based on the deep continuous filter of the convolutional neural network SchNet.^237,238^ It can also be integrated with the recently updated SPaiNN,^239^ which uses the equivariant polarizable neural network for atomic interactions PaiNN,^240^ an equivariant version of SchNet. This integrated framework, as illustrated in Figure 28, enables the simulation of molecular dynamics on a long time scale based on training data from COLUMBUS.

**Figure 28:**
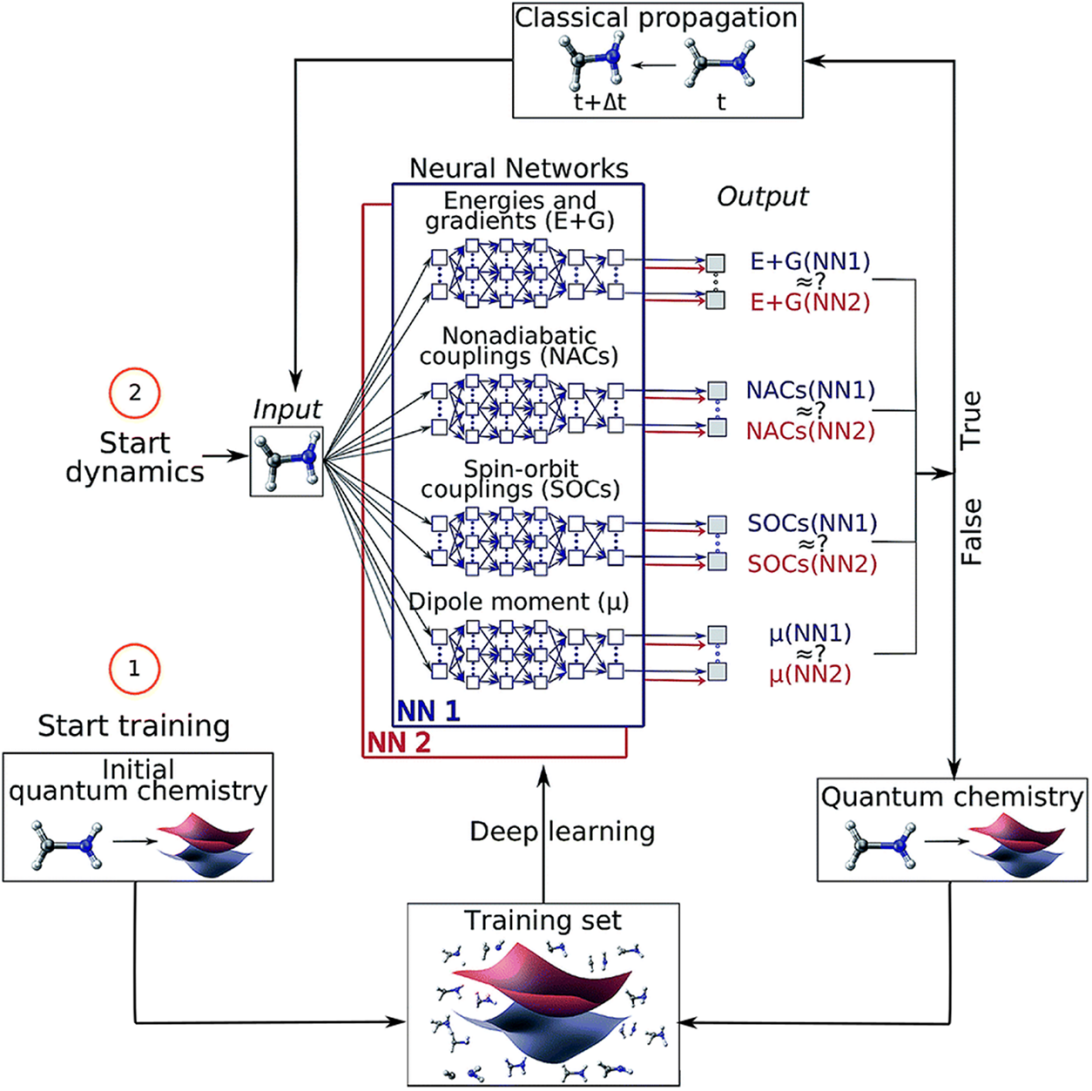
Concept of ML for surface hopping molecular dynamics, including training of ML potentials and training set generation. Adapted with permission from ref 228. Available under a CC-BY license. Copyright Westermayr et al.

ML modules can utilize the SHARC tools, such as Wigner sampling, to generate initial training sets for ML models, which are then employed to train neural network potentials for surface hopping. Additionally, a technique known as active learning or adaptive sampling is used to assess the reliability of the machine learning predictions. This approach ensures continuous refinement of the ML models in areas where their predictions deviate from quantum chemical reference calculations. This is achieved by comparing multiple ML predictions for each new molecular geometry accessed during photodynamics. If discrepancies arise between the ML models, quantum chemical reference calculations are performed, and the ML models are then retrained to restore accuracy. This iterative process enables longer simulation times as the accuracy of the ML potentials is maintained throughout the dynamics. These tools have been leveraged in SHARC to perform nanosecond-scale simulations for the methylenimmonium cation, the smallest member of protonated Schiff bases used to model the processes involved in vision.^228^ As the main chemical reaction is a *cis*–*trans* isomerization, multireference methods, such as those implemented in COLUMBUS, are essential for accuracy; however, propagating trajectories for over a nanosecond using COLUMBUS on the fly alone would not have been feasible.

Since this initial exploration of long-time scale photodynamics using ML, several additional methods and studies have been developed, including MLAtom 2 and PyRAI^2^MD.^241,242^ Recently, photodynamics simulations of the amino acid tyrosine were performed in conjunction with SHARC by integrating training data from various quantum chemical reference methods. This integration facilitated the discovery of roaming atoms—radicals that migrate around molecules and potentially lead to photodamage.^243^ While these studies have revealed intriguing aspects of natural photodynamic processes, numerous challenges remain in extending ML-driven photodynamics to more complex systems, particularly those with environmental influences.

One of the biggest challenges is the transferability of ML potentials across chemical compound space.^244,245^ Generating reference data for excited states is much more difficult.^229^ and accurate modeling of conical intersections—regions where electronic states converge and facilitate nonradiative transitions—is essential but challenging to achieve with ML models, which are typically smooth functions. Recent advances in the use of companion matrices show promise in solving this problem.^244,245^ Despite these obstacles, ML-driven dynamics, as demonstrated in studies of molecules such as the methylenimmonium cation, show that neural networks can provide both efficiency and precision for complex processes in excited states. Furthermore, high-quality data obtained with programs such as COLUMBUS are particularly beneficial for these fundamental studies.

### 6.3 Toward MD of Silaethylene with Nonadiabatic and Spin–Orbit Couplings

Molecules with photoexcited π-bonds exhibit fascinating dynamics that involve a complex interplay of torsion, pyramidalization, and hydrogen transfer en route to the conical intersection seam, facilitating a transition to the electronic ground state. Silaethylene (SiCH_4_) is a prime example of a polar π-bond and a computationally feasible model for ab initio nonadiabatic molecular dynamics (NAMD) studies. The previous NAMD study of silaethylene by Zechmann et al.^246^ focused on low-lying singlet potential energy surfaces. However, incorporating intersystem crossing (ISC) via the inclusion of the triplet manifold into the analysis is crucial, as ISC can be significant in systems with non-negligible spin–orbit coupling due to the presence of heavier atoms.

The availability of analytical gradients, nonadiabatic couplings, and spin–orbit couplings at the MRCI level within the COLUMBUS program makes this code particularly suitable for accurate NAMD studies of smaller molecules.^13,14,247,248^ In previous studies, we implemented surface hopping MD with nonadiabatic and spin–orbit couplings by interfacing the Newton-X program with different quantum chemistry codes, and presently, we extended this interface to COLUMBUS, including spin–orbit couplings from MRCI.^249–251^

However, it is crucial to address a well-known limitation of the MRCI method: the lack of size extensivity, which can lead to inaccuracies in energy calculations. To verify the reliability of the MR-CISD results, we compare them to the MR-AQCC and Mukherjee’s multireference coupled cluster theory, incorporating triples perturbatively (MR-CCSD­(T)).^252,253^ While MR-AQCC gradients have been implemented,^55^ there are no NAD and SO couplings available for either method, meaning that they cannot be used in the MD directly.

We employed the SA-CASSCF (2,2) method in the aug-cc-pVDZ basis to optimize different structures of silaethylene in their S_0_ and T_1_ states. We have investigated the planar silaethylene structure, as well as structures with a diradical center at carbon and silicon. We also investigated a 90-degree twisted structure and one structure along an MD trajectory, where the T_1_ state was below the S_0_. For these geometries, we then performed single-point calculations at the MR-CISD, MR-AQCC, and Mukhejee’s MR-CCSD­(T) levels. We computed three lowest-lying states (S_0_, S_1_, and T_1_) for MR-CISD and two states (S_0_ and T_1_) for MR-AQCC using COLUMBUS. Mukherjee’s MRCCSD­(T) calculations have been performed using Orca.^254^

The results of the aforementioned methods for the nine structures of SiCH_4_ (labeled A to I) are presented in Figure 29. Structures A and B represent the optimized S_0_ and T_1_ local minima of silaethylene, respectively. Structure A represents the global minimum, serving as the reference for the relative energies displayed. Geometries C and D were optimized with the Si–C dihedral angle constrained to 90° and are not local minima. A twisted structure with T_1_ lying below S_0_ at the MR-CISD level was obtained from a molecular dynamics trajectory and is denoted by E. Finally, we considered two diradical isomers of silaethylene: SiH_3_CH and CH_3_SiH. F and G denote the S_0_ and T_1_ optimized structures of SiH_3_CH, while H and I represent the S_0_ and T_1_ optimized structures of CH_3_SiH. Geometries F–I have been verified as local minima in the respective electronic state. For MR-CISD, we also computed the S_1_ energies.

**Figure 29:**
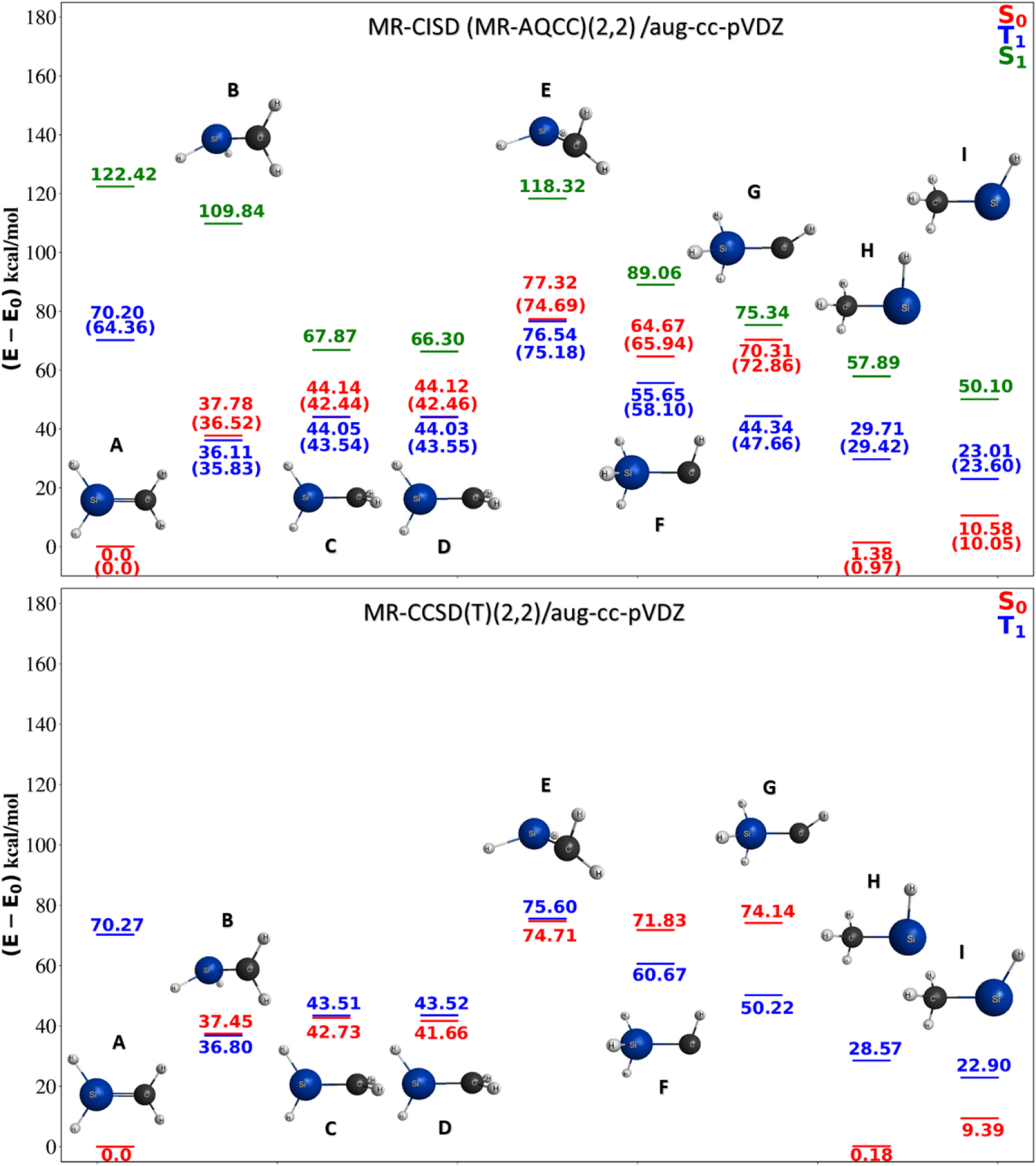
S_0_, T_1_, and S_1_ energy levels (relative to S_0_ of structure A) at MR-CISD and MR-AQCC (upper panel, MRAQCC energies in parentheses), and Mukherjee’s MR-CCSD­(T) levels (bottom panel) with CAS­(2,2) active space in the aug-cc-pVDZ basis set for several relevant structures. The geometries (except for E) have been optimized at the SA-CASSCF­(2,2)/aug-cc-pVDZ level as follows: (A) Optimized S_0_, (B) optimized T_1_, (C) optimized S_0_ with constraints, (D) optimized T_1_ with constraints, (E) geometry from MD, (F) optimized S_0_ of SiH_3_CH diradical, (G) optimized T_1_ of SiH_3_CH diradical, (H) optimized S_0_ of CH_3_SiH diradical, (I) optimized T_1_ of CH_3_SiH diradical. All structures except C–E are local minima.

The relative energies of all structures, as well as the separations between the singlet and triplet states, show an excellent agreement across all three computational methods. For structure B, we also computed the MkCCSD­(T) S-T gap in the aug-cc-pVTZ basis, yielding 1.1 kcal/mol; in contrast, the results in the aug-cc-pVDZ basis are 0.65 kcal/mol. The aug-cc-pVDZ basis, which is computationally affordable for the MD study, thus appears to be a suitable choice.

In some cases, when the energy separation between singlet and triplet states is minimal, the relative order of the two states changes depending on the method. This happens particularly for the nonequilibrium structures C, D, and E, which are easily energetically accessible in an MD started from S_1_ at the equilibrium geometry A. In our opinion, the swapping of extremely closely lying states should not have an adverse influence on the MD. The diradical equilibrium structures F–I have a relatively weak multireference character (second largest CI coefficient about 0.2) and have larger singlet–triplet gaps than the nonequilibrium structures C-E. The SiH_3_CH diradical (F, G) always has a triplet ground state and is relatively high in energy compared to the stable molecule A. However, the CH_3_SiH diradical structure (H and I) has a singlet ground state. Its singlet local minimum (H) is almost degenerate in energy to the silaethylene molecule A and, depending on the energy barrier might be a stable isomer and might be reachable as a product of a photoisomerization of the molecule A after excitation to S_1_, which might also include triplet intermediates. A photorelaxation back to A is another option, as is the generation of products in the triplet state, which is energetically accessible. A NAMD study, including the SOC effects, will be needed to elucidate these processes.

To summarize, our results confirm that MR-CISD is both computationally efficient and reliable as a basis for NAMD simulations of the photochemistry of silaethylene after excitation to the S_1_ state, which will be the subject of a forthcoming paper.

## 7 Conclusions and Outlook

The topics covered in this work illustrate the broad applicability of COLUMBUS in recent years. The need for multireference treatments is particularly evident in the section on open-shell systems, where di- and polyradicals serve as key examples. In the context of excited states, we addressed the challenge of achieving a balanced description of ionic and covalent states in hexatriene, as well as the construction of charge-transfer and doubly excited states. Spin–orbit MRCI calculations exemplify the extension of COLUMBUS into more demanding theoretical areas.

COLUMBUS has long been at the forefront of surface-hopping dynamics, owing to the availability of analytic energy gradients and nonadiabatic coupling vectors. However, the computational cost of performing MRCI-based on-the-fly dynamics has traditionally limited its application to small systems. As shown in the photodynamics section, this limitation is now being overcome by integrating machine learning techniques, which promise to significantly extend the scope of these simulations.

Recent code developments span several areas, including the computation of spin densities at the MCSCF level and the implementation of empirical scaling procedures designed to correct the imbalance between ionic and covalent states. While this issue has also been addressed by systematically expanding the wave function, scaling approaches provide a more computationally efficient alternative. The treatment of electronic resonances using complex absorbing potentials and simulations of the dynamics of highly excited states involved in attosecond photoemission spectrscopy further demonstrate COLUMBUS’s ability to tackle problems beyond the scope of conventional quantum chemistry.

In summary, the diverse topics presented here are unified by a common foundation in multireference theory. Together, they highlight both the feasibility and the necessity of applying multireference methods to an increasingly diverse range of complex chemical and spectroscopic problems.

## References

[1] Plasser F., Barbatti M., Aquino A. J. A., Lischka H. (2012). Electronically Excited
States and Photodynamics: A Continuing Challenge. Theor. Chem. Acc..

[2] González L., Escudero D., Serrano-Andrés L. (2012). Progress and
Challenges
in the Calculation of Electronic Excited States. ChemPhysChem.

[3] Boggio-Pasqua M., Bearpark M. J., Klene M., Robb M. A. (2004). A Computational
Strategy for Geometry Optimization of Ionic and Covalent Excited States,
Applied to Butadiene and Hexatriene. J. Chem.
Phys..

[4] Borden W. T., Davidson E. R. (1996). The Importance of Including Dynamic Electron Correlation
in *Ab Initio* Calculations. Acc. Chem. Res..

[5] Roos B. O., Andersson K., Fülscher M. P. (1992). Towards an Accurate Molecular Orbital
Theory for Excited States: The Benzene Molecule. Chem. Phys. Lett..

[6] Grimme S., Hansen A. (2015). A Practicable Real-Space Measure
and Visualization
of Static Electron-Correlation Effects. Angew.
Chem., Int. Ed..

[7] Shavitt, I. The Graphical Unitary Group Approach and Its Application to Direct Configuration Interaction Calculations. In The Unitary Group for the Evaluation of Electronic Energy Matrix Elements; Hinze, J. , Ed.; Lecture Notes in Chemistry; Springer: Berlin, 1981; pp 51–99.

[8] Shepard R., Shavitt I., Pitzer R. M., Comeau D. C., Pepper M., Lischka H., Szalay P. G., Ahlrichs R., Brown F. B., Zhao J.-G. (1988). A Progress Report
on the Status of the COLUMBUSMRCI
Program System. Int. J. Quantum Chem..

[9] Plasser, F. ; Lischka, H. Multi-Reference Configuration Interaction. In Quantum Chemistry and Dynamics of Excited States; Wiley, 2020; Chapter 9, pp 277–297.

[10] Müller T. (2009). Large-Scale
Parallel Uncontracted Multireference-Averaged Quadratic Coupled Cluster:
The Ground State of the Chromium Dimer Revisited. J. Phys. Chem. A.

[11] Plasser F., Pašalić H., Gerzabek M. H., Libisch F., Reiter R., Burgdörfer J., Müller T., Shepard R., Lischka H. (2013). The Multiradical Character
of One-
and Two-Dimensional Graphene Nanoribbons. Angew.
Chem., Int. Ed..

[12] Lischka H., Shepard R., Müller T., Szalay P. G., Pitzer R. M., Aquino A. J. A., do
Nascimento M. M. A., Barbatti M., Belcher L. T., Blaudeau J.-P. (2020). The Generality of the GUGA MRCI Approach in
COLUMBUS for Treating Complex Quantum Chemistry. J. Chem. Phys..

[13] Yabushita S., Zhang Z., Pitzer R. M. (1999). Spin-Orbit Configuration Interaction
Using the Graphical Unitary Group Approach and Relativistic Core Potential
and Spin-Orbit Operators. J. Phys. Chem. A.

[14] Mai S., Müller T., Plasser F., Marquetand P., Lischka H., González L. (2014). Perturbational
Treatment of Spin-Orbit
Coupling for Generally Applicable High-Level Multi-Reference Methods. J. Chem. Phys..

[15] Langhoff S. R., Davidson E. R. (1974). Configuration Interaction
Calculations on the Nitrogen
Molecule. Int. J. Quantum Chem..

[16] Szalay, P. G. Configuration Interaction: Corrections for Size-Consistency. In Encyclopedia of Computational Chemistry; Wiley, 2005.

[17] do
Monte S. A., Spada R. F. K., Alves R. L. R., Belcher L., Shepard R., Lischka H., Plasser F. (2023). Quantification of the
Ionic Character of Multiconfigurational Wave Functions: The Qat Diagnostic. J. Phys. Chem. A.

[18] Pople J. A., Seeger R., Krishnan R. (2009). Variational
Configuration Interaction
Methods and Comparison with Perturbation Theory. Int. J. Quantum Chem..

[19] Davidson E. R., Silver D. W. (1977). Size Consistency
in the Dilute Helium Gas Electronic
Structure. Chem. Phys. Lett..

[20] Szalay P. G., Bartlett R. J. (1993). Multi-Reference
Averaged Quadratic Coupled-Cluster
Method: A Size-Extensive Modification of Multi-Reference CI. Chem. Phys. Lett..

[21] Aidas K., Angeli C., Bak K. L., Bakken V., Bast R., Boman L., Christiansen O., Cimiraglia R., Coriani S., Dahle P. (2014). The Dalton
Quantum Chemistry
Program System. WIREs Comput. Mol. Sci..

[22] Li
Manni G., Fdez, Galván I., Alavi A., Aleotti F., Aquilante F., Autschbach J., Avagliano D., Baiardi A., Bao J. J., Battaglia S. (2023). The OpenMolcas *Web*: A Community-Driven Approach
to Advancing Computational Chemistry. J. Chem.
Theory Comput.

[23] Aprà E., Bylaska E. J., de Jong W. A., Govind N., Kowalski K., Straatsma T. P., Valiev M., van Dam H. J. J., Alexeev Y., Anchell J. (2020). NWChem: Past, Present, and Future. J. Chem. Phys..

[24] Aquilante F., Autschbach J., Carlson R. K., Chibotaru L. F., Delcey M. G., De Vico L., Fdez, Galván I., Ferré N., Frutos L. M., Gagliardi L. (2016). Molcas 8: New Capabilities for Multiconfigurational Quantum Chemical
Calculations across the Periodic Table. J. Comput.
Chem..

[25] COLUMBUS . an Ab Initio Electronic Structure Program. https://gitlab.com/columbus-program-system/columbus. (accessed June 09, 2025).

[26] Spada R. F. K., Franco M. P., Nieman R., Aquino A. J. A., Shepard R., Plasser F., Lischka H. (2023). Spin-Density Calculation via the
Graphical Unitary Group Approach. Mol. Phys..

[27] Shavitt I. (2009). Graph Theoretical
Concepts for the Unitary Group Approach to the Many-Electron Correlation
Problem. Int. J. Quantum Chem..

[28] Gidofalvi G., Shepard R. (2009). The Evaluation of Spin-density Matrices within the
Graphically Contracted Function Method. Int.
J. Quantum Chem..

[29] Nakano M., Champagne B. (2015). Theoretical
Design of Open-Shell Singlet Molecular
Systems for Nonlinear Optics. J. Phys. Chem.
Lett..

[30] Messelberger J., Grünwald A., Pinter P., Hansmann M. M., Munz D. (2018). Carbene Derived
Diradicaloids – Building Blocks for Singlet Fission?. Chem. Sci..

[31] Yu C. P., Chowdhury R., Fu Y., Ghosh P., Zeng W., Mustafa T. B. E., Grüne J., Walker L. E., Congrave D. G., Chua X. W., Murto P., Rao A., Sirringhaus H., Plasser F., Grey C. P., Friend R. H., Bronstein H. (2024). Near-Infrared
Luminescent Open-Shell π-Conjugated Systems with a Bright Lowest-Energy
Zwitterionic Singlet Excited State. Sci. Adv..

[32] Rudebusch G. E., Zafra J. L., Jorner K., Fukuda K., Marshall J. L., Arrechea-Marcos I., Espejo G. L., Ponce Ortiz R., Gómez-García C. J., Zakharov L. N., Nakano M., Ottosson H., Casado J., Haley M. M. (2016). Diindeno-Fusion
of an Anthracene as a Design Strategy for Stable Organic Biradicals. Nat. Chem..

[33] Nguyen T. D., Ehrenfreund E., Vardeny Z. V. (2012). Spin-Polarized Light-Emitting Diode
Based on an Organic Bipolar Spin Valve. Science.

[34] Szwarc M. (1947). Some Remarks
on the CH2CH2Molecule. Discuss. Faraday Soc..

[35] Coulson C. A., Craig D. P., Maccoll A., Pullman A. (1947). P-Quinodimethane and
Its Diradical. Discuss. Faraday Soc..

[36] Chagas J. C. V., Milanez B. D., Oliveira V. P., Pinheiro M., Ferrão L. F. A., Aquino A. J. A., Lischka H., Machado F. B. C. (2024). A Multi-descriptor
Analysis of Substituent Effects
on the Structure and Aromaticity of Benzene Derivatives: Π-Conjugation
versus Charge Effects. J. Comput. Chem..

[37] Matasović L., Bronstein H., Friend R. H., Plasser F. (2024). Classification and
Quantitative Characterisation of the Excited States of π-Conjugated
Diradicals. Faraday Discuss..

[38] Schulten K., Ohmine I., Karplus M. (1976). Correlation
Effects in the Spectra
of Polyenes. J. Chem. Phys..

[39] Kimber, P. ; Plasser, F. Classification and Analysis of Molecular Excited States. In Comprehensive Computational Chemistry; Elsevier, 2024; pp 55–83.

[40] Plasser F. (2020). TheoDORE:
A Toolbox for a Detailed and Automated Analysis of Electronic Excited
State Computations. J. Chem. Phys..

[41] Véril M., Scemama A., Caffarel M., Lipparini F., Boggio-Pasqua M., Jacquemin D., Loos P. (2021). QUESTDB: A Database
of Highly Accurate Excitation Energies for the Electronic Structure
Community. WIREs Comput. Mol. Sci..

[42] Chen X. K., Kim D., Brédas J. L. (2018). Thermally Activated Delayed Fluorescence
(TADF) Path toward Efficient Electroluminescence in Purely Organic
Materials: Molecular Level Insight. Acc. Chem.
Res..

[43] Kimber P., Plasser F. (2023). Energy Component Analysis for Electronically
Excited
States of Molecules: Why the Lowest Excited State Is Not Always the
HOMO/LUMO Transition. J. Chem. Theory Comput..

[44] Kimber P., Plasser F. (2020). Toward an Understanding of Electronic Excitation Energies
beyond the Molecular Orbital Picture. Phys.
Chem. Chem. Phys..

[45] Snyder J. W., Parrish R. M., Martínez T. J. (2017). α-CASSCF:
An Efficient, Empirical
Correction for SA-CASSCF to Closely Approximate MS-CASPT2 Potential
Energy Surfaces. J. Phys. Chem. Lett..

[46] Frutos L. M., Andruniów T., Santoro F., Ferré N., Olivucci M. (2007). Tracking the Excited-State Time Evolution of the Visual
Pigment with Multiconfigurational Quantum Chemistry. Proc. Natl. Acad. Sci. U.S.A..

[47] Carlson R. K., Truhlar D. G., Gagliardi L. (2015). Multiconfiguration
Pair-Density Functional
Theory: A Fully Translated Gradient Approximation and Its Performance
for Transition Metal Dimers and the Spectroscopy of Re2Cl82. J. Chem. Theory Comput.

[48] Gdanitz R. J., Ahlrichs R. (1988). The Averaged Coupled-Pair
Functional (ACPF): A Size-Extensive
Modification of MR CI­(SD). Chem. Phys. Lett..

[49] Szalay P. G., Bartlett R. J. (1995). Approximately Extensive Modifications of the Multireference
Configuration Interaction Method: A Theoretical and Practical Analysis. J. Chem. Phys..

[50] Szalay P. G., Müller T., Gidofalvi G., Lischka H., Shepard R. (2012). Multiconfiguration
Self-Consistent Field and Multireference Configuration Interaction
Methods and Applications. Chem. Rev..

[51] Lischka H., Nachtigallová D., Aquino A. J. A., Szalay P. G., Plasser F., Machado F. B. C., Barbatti M. (2018). Multireference Approaches for Excited
States of Molecules. Chem. Rev..

[52] Szalay, P. G. Towards State-Specific Formulation of Multireference Coupled-Cluster Theory: Coupled Electron Pair Approximations (CEPA) Leading to Multireference Configuration Interaction (MR-CI) Type Equations. In Recent Advances in Coupled-Cluster Methods; Bartlett, R. J. , Ed.; World Scientific, 1997; pp 81–123.

[53] Szalay, P. G. Configuration Interaction: Corrections for Size-Consistency. In Encyclopedia of Computational Chemistry; Wiley, 1998.

[54] Szalay P. G., Müller T., Lischka H. (2000). Excitation Energies and Transition
Moments by the Multireference Averaged Quadratic Coupled Cluster (MR-AQCC)
Method. Phys. Chem. Chem. Phys..

[55] Lischka H., Shepard R., Pitzer R. M., Shavitt I., Dallos M., Müller T., Szalay P. G., Seth M., Kedziora G. S., Yabushita S., Zhang Z. (2001). High-Level Multireference Methods
in the Quantum-Chemistry Program System COLUMBUS: Analytic MR-CISD
and MR-AQCC Gradients and MR-AQCC-LRT for Excited States, GUGA Spin–Orbit
CI and Parallel CI Density. Phys. Chem. Chem.
Phys..

[56] Horn S., Plasser F., Müller T., Libisch F., Burgdörfer J., Lischka H. (2014). A Comparison of Singlet and Triplet States for One-
and Two-Dimensional Graphene Nanoribbons Using Multireference Theory. Theor. Chem. Acc..

[57] Milanez B. D., Chagas J. C. V., Pinheiro M., Aquino A. J. A., Lischka H., Machado F. B. C. (2020). Effects
on the Aromaticity and on
the Biradicaloid Nature of Acenes by the Inclusion of a Cyclobutadiene
Linkage. Theor. Chem. Acc..

[58] Müller T., Dallos M., Lischka H., Dubrovay Z., Szalay P. G. (2001). A Systematic
Theoretical Investigation of the Valence Excited States of the Diatomic
Molecules B 2, C 2, N 2 and O 2. Theor. Chem.
Acc.: Theory, Comput., Model. (Theor. Chim. Acta).

[59] Christiansen O., Jorgensen P., Hättig C. (1998). Response Functions from Fourier Component
Variational Perturbation Theory Applied to a Time-Averaged Quasienergy. Int. J. Quantum Chem..

[60] Dunning T. H. (1989). Gaussian
Basis Sets for Use in Correlated Molecular Calculations. I. The Atoms
Boron through Neon and Hydrogen. J. Chem. Phys..

[61] Seritan S., Bannwarth C., Fales B. S., Hohenstein E. G., Isborn C. M., Kokkila-Schumacher S.
I. L., Li X., Liu F., Luehr N., Snyder J. W., Song C., Titov A. V., Ufimtsev I. S., Wang L., Martínez T. J. (2021). TeraChem:
A Graphical Processing Unit-accelerated Electronic Structure Package
for large-scale Ab Initio Molecular Dynamics. WIREs Comput. Mol. Sci..

[62] NVIDIA Corporation . NVIDIA CUDA Fortran Programming Guide 2025. https://docs.nvidia.com/hpc-sdk/compilers/cuda-fortran-prog-guide/index.htmlhttps://docs.nvidia.com/hpc-sdk/compilers/cuda-fortran-prog-guide/index.html. (accessed January 31, 2025).

[63] Wilkinson K. A., Sherwood P., Guest M. F., Naidoo K. J. (2011). Acceleration of
the GAMESS-UK Electronic Structure Package on Graphical Processing
Units. J. Comput. Chem..

[64] Frisch, M. J. ; Trucks, G. W. ; Schlegel, H. B. Gaussian 16; Gaussian, Inc.: Wallingford, 2016.

[65] Sun Q., Zhang X., Banerjee S., Bao P., Barbry M., Blunt N. S., Bogdanov N. A., Booth G. H., Chen J., Cui Z.-H., Eriksen J. J., Gao Y., Guo S., Hermann J., Hermes M. R., Koh K., Koval P., Lehtola S., Li Z., Liu J., Mardirossian N., McClain J. D., Motta M., Mussard B., Pham H. Q., Pulkin A., Purwanto W., Robinson P. J., Ronca E., Sayfutyarova E. R., Scheurer M., Schurkus H. F., Smith J. E. T., Sun C., Sun S.-N., Upadhyay S., Wagner L. K., Wang X., White A., Whitfield J. D., Williamson M. J., Wouters S., Yang J., Yu J. M., Zhu T., Berkelbach T. C., Sharma S., Sokolov A. Yu., Chan G. K.-L. (2020). Recent Developments in the PySCF Program Package. J. Chem. Phys..

[66] Roos, B. O. ; Siegbahn, P. E. M. The Direct Configuration Interaction Method from Molecular Integrals. In Methods of Electronic Structure Theory; Schaefer, H. F., III , Ed.; Springer: New York, 1977; pp 277–318.

[67] Paldus J. (1974). Group Theoretical
Approach to the Configuration Interaction and Perturbation Theory
Calculations for Atomic and Molecular Systems. J. Chem. Phys..

[68] Ahlrichs R., Böhm H., Ehrhardt C., Scharf P., Schiffer H., Lischka H., Schindler M. (1985). Implementation of an Electronic Structure
Program System on the CYBER 205. J. Comput.
Chem..

[69] Werner H.-J., Reinsch E. (1982). The Self-consistent Electron Pairs Method for Multiconfiguration
Reference State Functions. J. Chem. Phys..

[70] Davidson E.
R. (1975). The Iterative
Calculation of a Few of the Lowest Eigenvalues and Corresponding Eigenvectors
of Large Real-Symmetric Matrices. J. Comput.
Phys..

[71] Shepard R., Shavitt I., Lischka H. (2002). Reducing I/O Costs
for the Eigenvalue
Procedure in Large-scale Configuration Interaction Calculations. J. Comput. Chem..

[72] Arumainayagam C. R., Garrod R. T., Boyer M. C., Hay A. K., Bao S. T., Campbell J. S., Wang J., Nowak C. M., Arumainayagam M. R., Hodge P. J. (2019). Extraterrestrial
Prebiotic Molecules: Photochemistry *vs.* Radiation
Chemistry of Interstellar Ices. Chem. Soc. Rev..

[73] Boyer M. C., Rivas N., Tran A. A., Verish C. A., Arumainayagam C. R. (2016). The Role
of Low-Energy (≤ 20 EV) Electrons in Astrochemistry. Surf. Sci..

[74] Swiderek P. (2006). Fundamental
Processes in Radiation Damage of DNA. Angew.
Chem., Int. Ed..

[75] Alizadeh E., Orlando T. M., Sanche L. (2015). Biomolecular
Damage Induced by Ionizing
Radiation: The Direct and Indirect Effects of Low-Energy Electrons
on DNA. Annu. Rev. Phys. Chem..

[76] Arumainayagam C. R., Lee H.-L., Nelson R. B., Haines D. R., Gunawardane R. P. (2010). Low-Energy
Electron-Induced Reactions in Condensed Matter. Surf. Sci. Rep.

[77] Herbert, J. M. The Quantum Chemistry of Loosely-Bound Electrons. In Reviews in Computational Chemistry; Parrill, A. L. ; Lipkowitz, K. B. , Eds.; Wiley, 2015;Chapter 8, Vol. 28, pp 391–517.

[78] Kawarai Y., Weber Th., Azuma Y., Winstead C., McKoy V., Belkacem A., Slaughter D. S. (2014). Dynamics of the Dissociating Uracil
Anion Following Resonant Electron Attachment. J. Phys. Chem. Lett..

[79] Klaiman S., Cederbaum L. S. (2015). Barrierless
Single-Electron-Induced *cis*–*rans* Isomerization. Angew. Chem..

[80] Fennimore M. A., Karsili T. N. V., Matsika S. (2017). Mechanisms of H and CO Loss from
the Uracil Nucleobase Following Low Energy Electron Irradiation. Phys. Chem. Chem. Phys..

[81] Fennimore M. A., Matsika S. (2018). Electronic Resonances
of Nucleobases Using Stabilization
Methods. J. Phys. Chem. A.

[82] Loupas A., Gorfinkiel J. D. (2019). Shape and
Core-Excited Resonances in Electron Scattering
from Alanine. J. Chem. Phys..

[83] Thodika M., Mackouse N., Matsika S. (2020). Description
of Two-Particle One-Hole
Electronic Resonances Using Orbital Stabilization Methods. J. Phys. Chem. A.

[84] Gayvert, J. R. An Open-Source Program for Studying Resonances in Molecules 2021 https://github.com/gayverjr/opencap. (accessed June 08, 2025).

[85] Moiseyev, N. Non-Hermitian Quantum Mechanics; Cambridge University Press, 2011.

[86] Riss U. V., Meyer H.-D. (1993). Calculation of Resonance
Energies and Widths Using
the Complex Absorbing Potential Method. J. Phys.
B: At., Mol. Opt. Phys..

[87] Jagau T.-C., Zuev D., Bravaya K. B., Epifanovsky E., Krylov A. I. (2014). A Fresh Look at Resonances and Complex
Absorbing Potentials:
Density Matrix-Based Approach. J. Phys. Chem.
Lett..

[88] Sommerfeld T., Santra R. (2001). Efficient Method to Perform CAP/CI Calculations for
Temporary Anions. Int. J. Quantum Chem..

[89] Santra R., Cederbaum L. S. (2001). An Efficient
Combination of Computational Techniques
for Investigating Electronic Resonance States in Molecules. J. Chem. Phys..

[90] Thodika M., Matsika S. (2022). Projected Complex Absorbing
Potential Multireference
Configuration Interaction Approach for Shape and Feshbach Resonances. J. Chem. Theory Comput.

[91] Thodika, M. Development and Benchmarking of Hermitian and Non-Hermitian Methods for Negative Ion Resonances; Temple University, 2022.

[92] Majety V. P., Zielinski A., Scrinzi A. (2015). Photoionization of Few Electron Systems:
A Hybrid Coupled Channels Approach. New J. Phys..

[93] Chundayil H., Majety V. P., Scrinzi A. (2024). The Hybrid Anti-Symmetrized Coupled
Channels Method (HaCC) for the TRecX Code. Comput.
Phys. Commun..

[94] Majety V. P., Scrinzi A. (2015). Dynamic Exchange in the Strong Field
Ionization of
Molecules. Phys. Rev. Lett..

[95] Tao L., Scrinzi A. (2012). Photo-Electron Momentum
Spectra from Minimal Volumes:
The Time-Dependent Surface Flux Method. New
J. Phys..

[96] Majety V. P., Scrinzi A. (2017). Multielectron Effects in Strong-Field
Ionization of
CO2: Impact on Differential Photoelectron Spectra. Phys. Rev. A.

[97] Geim A. K., Novoselov K. S. (2007). The Rise of Graphene. Nat. Mater..

[98] Nguyen B. H., Nguyen V. H. (2016). Promising Applications of Graphene and Graphene-Based
Nanostructures. Adv. Nat. Sci.: Nanosci. Nanotechnol..

[99] Gu Y., Qiu Z., Müllen K. (2022). Nanographenes
and Graphene Nanoribbons
as Multitalents of Present and Future Materials Science. J. Am. Chem. Soc..

[100] Nieman R., Silva N. J., Aquino A. J. A., Haley M. M., Lischka H. (2020). Interplay of Biradicaloid Character
and Singlet/Triplet
Energy Splitting for *Cis* -/ *Trans* -Diindenoacenes and Related Benzothiophene-Capped Oligomers as Revealed
by Extended Multireference Calculations. J.
Org. Chem..

[101] Kubo T. (2015). Phenalenyl-Based Open-Shell Polycyclic Aromatic Hydrocarbons. Chem. Rec..

[102] Sánchez-Grande A., Urgel J. I., Cahlík A., Santos J., Edalatmanesh S., Rodríguez-Sánchez E., Lauwaet K., Mutombo P., Nachtigallová D., Nieman R., Lischka H., de la Torre B., Miranda R., Gröning O., Martín N., Jelínek P., Écija D. (2020). Diradical Organic One-Dimensional
Polymers Synthesized on a Metallic Surface. Angew. Chem., Int. Ed..

[103] Bettinger H. F. (2010). Electronic Structure of Higher Acenes
and Polyacene:
The Perspective Developed by Theoretical Analyses. Pure Appl. Chem..

[104] Ahmed J., Mandal S. K. (2022). Phenalenyl Radical:
Smallest Polycyclic
Odd Alternant Hydrocarbon Present in the Graphene Sheet. Chem. Rev..

[105] Kubo T. (2023). Closed-Shell and Open-Shell Dual
Nature of Singlet Diradical Compounds. Pure
Appl. Chem..

[106] Takatsuka K., Fueno T., Yamaguchi K. (1978). Distribution
of Odd Electrons in Ground-State Molecules. Theor. Chim. Acta.

[107] Head-Gordon M. (2003). Characterizing
Unpaired Electrons from the One-Particle
Density Matrix. Chem. Phys. Lett..

[108] Plasser F., Wormit M., Dreuw A. (2014). New Tools for the Systematic
Analysis and Visualization of Electronic Excitations. I. Formalism. J. Chem. Phys..

[109] Bauer C. A., Hansen A., Grimme S. (2017). The Fractional
Occupation
Number Weighted Density as a Versatile Analysis Tool for Molecules
with a Complicated Electronic Structure. Chem.
- Eur. J..

[110] Nieman R., Carvalho J. R., Jayee B., Hansen A., Aquino A. J. A., Kertesz M., Lischka H. (2023). Polyradical Character
Assessment Using Multireference Calculations and Comparison with Density-Functional
Derived Fractional Occupation Number Weighted Density Analysis. Phys. Chem. Chem. Phys..

[111] Carvalho J. R., Nieman R., Kertesz M., Aquino A. J. A., Hansen A., Lischka H. (2024). Multireference Calculations
on Bond
Dissociation and Biradical Polycyclic Aromatic Hydrocarbons as Guidance
for Fractional Occupation Number Weighted Density Analysis in DFT
Calculations. Theor. Chem. Acc..

[112] Staroverov V. N., Davidson E. R. (2000). Distribution of
Effectively Unpaired
Electrons. Chem. Phys. Lett..

[113] Tao J., Perdew J. P., Staroverov V. N., Scuseria G. E. (2003). Climbing the Density
Functional Ladder: Nonempirical Meta–Generalized Gradient Approximation
Designed for Molecules and Solids. Phys. Rev.
Lett..

[114] Weigend F., Ahlrichs R. (2005). Balanced Basis Sets of Split Valence,
Triple Zeta Valence and Quadruple Zeta Valence Quality for H to Rn:
Design and Assessment of Accuracy. Phys. Chem.
Chem. Phys..

[115] Tsuneda T., Hirao K. (2014). Long-range Correction for Density
Functional Theory. WIREs Comput. Mol. Sci..

[116] Bannwarth C., Ehlert S., Grimme S. (2019). GFN2-XTB—An
Accurate and Broadly Parametrized Self-Consistent Tight-Binding Quantum
Chemical Method with Multipole Electrostatics and Density-Dependent
Dispersion Contributions. J. Chem. Theory Comput.

[117] Bannwarth C., Caldeweyher E., Ehlert S., Hansen A., Pracht P., Seibert J., Spicher S., Grimme S. (2021). Extended Tight-binding
Quantum Chemistry Methods. WIREs Comput. Mol.
Sci..

[118] Bhatt M. D., Kim H., Kim G. (2022). Various Defects in
Graphene: A Review. RSC Adv..

[119] Machado F. B. C., Aquino A. J. A., Lischka H. (2014). The Diverse Manifold
of Electronic States Generated by a Single Carbon Defect in a Graphene
Sheet: Multireference Calculations Using a Pyrene Defect Model. ChemPhysChem.

[120] Pinheiro M., Cardoso D. V. V., Aquino A. J. A., Machado F. B. C., Lischka H. (2019). The Characterization
of Electronic Defect States of
Single and Double Carbon Vacancies in Graphene Sheets Using Molecular
Density Functional Theory. Mol. Phys..

[121] Nieman R., Das A., Aquino A. J. A., Amorim R. G., Machado F. B. C., Lischka H. (2017). Single and Double Carbon Vacancies
in Pyrene as First Models for Graphene Defects: A Survey of the Chemical
Reactivity toward Hydrogen. Chem. Phys..

[122] Nieman R., Oliveira V. P., Jayee B., Adelia A. J. A., Machado F. B. C., Lischka H. (2023). High-Level Multireference
Investigations on the Electronic States in Single-Vacancy (SV) Graphene
Defects Using a Pyrene-SV Model. J. Phys. Chem.
A.

[123] Hariharan P. C., Pople J. A. (1973). The Influence of
Polarization Functions
on Molecular Orbital Hydrogenation Energies. Theor. Chim. Acta.

[124] Shu C., Yang Z., Rajca A. (2023). From Stable Radicals to Thermally
Robust High-Spin Diradicals and Triradicals. Chem. Rev..

[125] Pozo I., Bogani L. (2024). A Perspective on Radicaloid Conjugated
Polycyclic Hydrocarbons. Trends Chem..

[126] Zeng W., Wu J. (2021). Open-Shell Graphene Fragments. Chem.

[127] Yu H., Jing Y., Heine T. (2025). Physics and Chemistry of Two-Dimensional
Triangulene-Based Lattices. Acc. Chem. Res..

[128] Mishra S., Beyer D., Eimre K., Liu J., Berger R., Gröning O., Pignedoli C. A., Müllen K., Fasel R., Feng X., Ruffieux P. (2019). Synthesis
and Characterization of π-Extended Triangulene. J. Am. Chem. Soc..

[129] Sanz-Rodrigo J., Ricci G., Olivier Y., Sancho-García J. C. (2021). Negative
Singlet–Triplet Excitation Energy Gap in Triangle-Shaped Molecular
Emitters for Efficient Triplet Harvesting. J.
Phys. Chem. A.

[130] Martínez-Carracedo G., Oroszlány L., García-Fuente A., Szunyogh L., Ferrer J. (2023). Electrically Driven
Singlet-Triplet Transition in Triangulene Spin-1 Chains. Phys. Rev. B.

[131] Su J., Telychko M., Song S., Lu J. (2020). Triangulenes: From
Precursor Design to On-Surface Synthesis and Characterization. Angew. Chem., Int. Ed..

[132] Pavliček N., Mistry A., Majzik Z., Moll N., Meyer G., Fox D. J., Gross L. (2017). Synthesis
and Characterization
of Triangulene. Nat. Nanotechnol..

[133] Su J., Fan W., Mutombo P., Peng X., Song S., Ondráček M., Golub P., Brabec J., Veis L., Telychko M., Jelínek P., Wu J., Lu J. (2021). On-Surface Synthesis
and Characterization of [7]­Triangulene
Quantum Ring. Nano Lett..

[134] Mishra S., Beyer D., Eimre K., Ortiz R., Fernández-Rossier J., Berger R., Gröning O., Pignedoli C. A., Fasel R., Feng X., Ruffieux P. (2020). Collective
All-Carbon Magnetism in Triangulene Dimers. Angew. Chem., Int. Ed..

[135] Yu H., Heine T. (2023). Magnetic Coupling Control
in Triangulene Dimers. J. Am. Chem. Soc..

[136] Yu H., Sun J., Heine T. (2023). Predicting Magnetic Coupling and
Spin-Polarization Energy in Triangulene Analogues. J. Chem. Theory Comput..

[137] Saleem Y., Steenbock T., Alhadi E. R. J., Pasek W., Bester G., Potasz P. (2024). Superexchange
Mechanism in Coupled
Triangulenes Forming Spin-1 Chains. Nano Lett..

[138] Weng T., Xu Z., Li K., Guo Y., Chen X., Li Z., Sun Z. (2024). 1,1′-Biolympicenyl:
A Stable Non-Kekulé Diradical with a Small Singlet and Triplet
Energy Gap. J. Am. Chem. Soc..

[139] Ortiz R., Catarina G., Fernández-Rossier J. (2023). Theory of
Triangulene Two-Dimensional Crystals. 2d Mater..

[140] Ovchinnikov A. A. (1978). Multiplicity
of the Ground State of Large Alternant
Organic Molecules with Conjugated Bonds - (Do Organic Ferromagnetics
Exist?). Theor Chim Acta.

[141] Lieb E. H. (1989). Two Theorems on the Hubbard Model. Phys. Rev. Lett..

[142] Itkis M. E., Chi X., Cordes A. W., Haddon R. C. (2002). Magneto-Opto-Electronic
Bistability in a Phenalenyl-Based Neutral Radical. Science.

[143] Yang Y., Blacque O., Sato S., Juríček M. (2021). Cycloparaphenylene–Phenalenyl
Radical and Its Dimeric Double Nanohoop. Angew.
Chem., Int. Ed..

[144] Pal S. K., Itkis M. E., Tham F. S., Reed R. W., Oakley R. T., Haddon R. C. (2005). Resonating Valence-Bond
Ground State
in a Phenalenyl-Based Neutral Radical Conductor. Science.

[145] Pariyar A., Vijaykumar G., Bhunia M., Dey S. Kr., Singh S. K., Kurungot S., Mandal S. K. (2015). Switching Closed-Shell
to Open-Shell Phenalenyl: Toward Designing Electroactive Materials. J. Am. Chem. Soc..

[146] Morita Y., Aoki T., Fukui K., Nakazawa S., Tamaki K., Suzuki S., Fuyuhiro A., Yamamoto K., Sato K., Shiomi D., Naito A., Takui T., Nakasuji K. (2002). A New Trend in Phenalenyl Chemistry:
A Persistent Neutral
Radical, 2,5,8-Tri-Tert-Butyl-1,3-Diazaphenalenyl, and the Excited
Triplet State of the Gablesyn-Dimer in the Crystal of Column Motif. Angew. Chem., Int. Ed..

[147] Das A., Müller T., Plasser F., Lischka H. (2016). Polyradical
Character of Triangular Non-Kekulé Structures, Zethrenes, p-Quinodimethane-Linked
Bisphenalenyl, and the Clar Goblet in Comparison: An Extended Multireference
Study. J. Phys. Chem. A.

[148] Hehre W. J., Ditchfield R., Pople J. A. (1972). Self Consistent
Molecular Orbital Methods. XII. Further Extensions of Gaussian-Type
Basis Sets for Use in Molecular Orbital Studies of Organic Molecules. J. Chem. Phys..

[149] Grimme S. (2006). Semiempirical GGA-type Density Functional
Constructed
with a Long-range Dispersion Correction. J.
Comput. Chem..

[150] Sirianni D. A., Song X., Wairegi S., Wang E. B., Mendoza-Gomez S. A., Luxon A., Zimmerley M., Nussdorf A., Filatov M., Hoffmann R., Parish C. A. (2023). Variations
on the Bergman Cyclization Theme: Electrocyclizations of Ionic Penta-,
Hepta-, and Octadiynes. J. Am. Chem. Soc..

[151] Jones R. R., Bergman R. G. (1972). P-Benzyne. Generation as an Intermediate
in a Thermal Isomerization Reaction and Trapping Evidence for the
1,4-Benzenediyl Structure. J. Am. Chem. Soc..

[152] Church D. F., Pryor W. A. (1985). Free-Radical Chemistry of Cigarette
Smoke and Its Toxicological Implications. Environ.
Health Perspect..

[153] Russell K. E., Tobolsky A. V. (1954). Diradicals in Solution: Role in Polymerization. J. Am. Chem. Soc..

[154] Slipchenko L. V., Krylov A. I. (2002). Singlet-Triplet
Gaps in Diradicals
by the Spin-Flip Approach: A Benchmark Study. J. Chem. Phys..

[155] Wang E. B., Parish C. A., Lischka H. (2008). An Extended
Multireference
Study of the Electronic States of *Para* -Benzyne. J. Chem. Phys..

[156] Vu K., Pandian J., Zhang B., Annas C., Parker A. J., Mancini J. S., Wang E. B., Saldana-Greco D., Nelson E. S., Springsted G., Lischka H., Plasser F., Parish C. A. (2024). Multireference Averaged
Quadratic Coupled Cluster (MR-AQCC)
Study of the Geometries and Energies for *Ortho* -, *Meta* - and *Para* -Benzyne. J. Phys. Chem. A.

[157] Scott T., Nieman R., Luxon A., Zhang B., Lischka H., Gagliardi L., Parish C. A. (2019). A Multireference
Ab Initio Study of the Diradical Isomers of Pyrazine. J. Phys. Chem. A.

[158] Pandian J., Vu K., Muya J. T., Parker A., Ancajas C. M. F., Saldana-Greco D., Yewer T., Parish C. (2025). A Highly Correlated,
Multireference Study of the Lowest Lying Singlet and Triplet States
of the Four Thiophene Diradicals. J. Comput.
Chem..

[159] Okamoto K., Kitagawa T., Takeuchi K., Komatsu K., Takahashi K. (1985). Isolation
of a Hydrocarbon Salt and Preparation of
a Hydrocarbon Which Heterolyses to a Carbocation and a Carbanion. J. Chem. Soc. Chem. Commun..

[160] Okamoto K., Kitagawa T., Takeuchi K., Komatsu K., Miyabo A. (1988). A Hydrocarbon Existing Uniquely in Solution: A Heterolytically
Dissociative Hydrocarbon That Produces the Corresponding Hydrocarbon
Salt by Crystallization. J. Chem. Soc. Chem.
Commun..

[161] Kitagawa T., Tanaka T., Murakita H., Nishikawa A., Takeuchi K. (2001). Reaction of Cyclopropenylium Ions with the Tert-Butyl-C60
Anion: Carbocation–Carbanion Coordination vs Salt Formation. Tetrahedron.

[162] Tanaka T., Kitagawa T., Komatsu K., Takeuchi K. (1997). Synthesis
of a Hydrocarbon Salt Having a Fullerene Framework. J. Am. Chem. Soc..

[163] Ventura E., Alves R. L. R., do
Monte S. A. (2024). The Kinetics of
Three Coupled Irreversible Elementary Reactions: Two Parallel Mixed
Second Order Reactions Followed by a First Order Reaction. J. Math. Chem..

[164] Ventura E., Rodrigues G. P., Leitão E. F. V., do Monte S. A. (2024). Theoretical Study of an Authentic
Hydrocarbon Ion Pair. ACS Omega.

[165] Alves R. L. R., Leitão E. F. V., Ventura E., do Monte S. A. (2025). A Genuine
Hydrocarbon Ion Pair More Stable Than Its Covalent Counterpart. A
Computational Study. J. Comput. Chem..

[166] Kolomnikova G. D., Parnes Z. N. (1967). Advances in the
Chemistry of the
Tropylium Ion. Russ. Chem. Rev..

[167] Koenig T., Chang J. C. (1978). Helium­(I) Photoelectron Spectrum
of Tropyl Radical. J. Am. Chem. Soc..

[168] McDonald R. N., Bianchina E. J., Tung C. C. (1991). Electron Photodetachment
of Cyclopentadienylidene Anion Radical in a Flowing Afterglow Apparatus:
EA and.DELTA.Hf.Degree. of Cyclopentadienylidene. J. Am. Chem. Soc..

[169] Silva A. J. F. W. H.
de S., Rodrigues G. P., Ventura E., do Monte S. A. (2024). Photodissociation and Formation of
an Ion-pair in CH2 FCl (HCFC-31). J. Comput.
Chem..

[170] Bezerra M. G., Leitão E. F. V., de Andrade R. B., Ventura E., do Monte S. A. (2021). Photochemistry
of Monohydrated Chloromethane:
Formation of Free and Hydrated Cl ^–^ and CH _3_
^+^ Ions from a Solvent-Shared Semi-Ion-Pair. J. Phys. Chem. A.

[171] Ventura E., do Monte S. A. (2020). Hydrogen-Bonded
Contact Ion Pair
in Gaseous Chloroethane: A Multi-Reference Configuration Interaction
with Singles and Doubles (MR-CISD) Study Including Extensivity Corrections. Theor. Chem. Acc..

[172] de Medeiros V. C., de Andrade R. B., Rodrigues G. P., Bauerfeldt G. F., Ventura E., Barbatti M., do Monte S. A. (2018). Photochemistry
of CF _3_ Cl: Quenching of Charged Fragments Is Caused by
Nonadiabatic Effects. J. Chem. Theory Comput..

[173] de Medeiros V. C., de Andrade R. B., Leitão E. F. V., Ventura E., Bauerfeldt G. F., Barbatti M., do Monte S. A. (2016). Photochemistry
of CH _3_ Cl: Dissociation and CH···Cl Hydrogen
Bond Formation. J. Am. Chem. Soc..

[174] Gagliardi L., Roos B. O. (2005). Quantum Chemical Calculations Show
That the Uranium Molecule U2 Has a Quintuple Bond. Nature.

[175] Roos B. O., Malmqvist P.-Å., Gagliardi L. (2006). Exploring
the Actinide–Actinide Bond: Theoretical Studies of the Chemical
Bond in Ac _2_, Th _2_, Pa _2_, and U _2_. J. Am. Chem. Soc..

[176] Knecht S., Jensen H. J. Aa., Saue T. (2019). Relativistic
Quantum
Chemical Calculations Show That the Uranium Molecule U2 Has a Quadruple
Bond. Nat. Chem..

[177] Peterson K. A. (2015). Correlation Consistent Basis Sets
for Actinides. I.
The Th and U Atoms. J. Chem. Phys..

[178] Aquilante F., Autschbach J., Baiardi A., Battaglia S., Borin V. A., Chibotaru L. F., Conti I., De Vico L., Delcey M., Galván I. F., Ferré N., Freitag L., Garavelli M., Gong X., Knecht S., Larsson E. D., Lindh R., Lundberg M., Malmqvist PÅ., Nenov A., Norell J., Odelius M., Olivucci M., Pedersen T. B., Pedraza-González L., Phung Q. M., Pierloot K., Reiher M., Schapiro I., Segarra-Martí J., Segatta F., Seijo L., Sen S., Sergentu D.-C., Stein C. J., Ungur L., Vacher M., Valentini A., Veryazov V. (2020). Modern Quantum Chemistry with [Open]­Molcas. J. Chem. Phys..

[179] Glendening E. D., Landis C. R., Weinhold F. (2012). Natural Bond Orbital
Methods. WIREs Comput. Mol. Sci..

[180] Glendening, E. D. ; Badenhoop, J. K. ; Reed, A. E. ; Carpenter, J. E. ; Bohmann, J. A. ; Morales, C. M. ; Karafiloglou, P. ; Landis, C. R. ; Weinhold, F. NBO 7.0. Madison, Wisconsin. 2018.

[181] Zaichenko, A. ; Autschbach, J. MolcasTo47 2024. https://github.com/jautschbach/molcasto47. (accessed March 24, 2025).

[182] Ciborowski S. M., Mitra A., Harris R. M., Liu G., Sharma P., Khetrapal N., Blankenhorn M., Gagliardi L., Bowen K. H. (2021). Metal–Metal Bonding in Actinide
Dimers: U2 and U2-. J. Am. Chem. Soc..

[183] Wiberg K. B. (1968). Application
of the Pople-Santry-Segal CNDO Method to
the Cyclopropylcarbinyl and Cyclobutyl Cation and to Bicyclobutane. Tetrahedron.

[184] Mayer I. (2007). Bond Order and Valence Indices: A
Personal Account. J. Comput. Chem..

[185] Khokhlov D., Belov A. (2021). Toward an Accurate *Ab Initio* Description of Low-Lying Singlet Excited States
of Polyenes. J. Chem. Theory Comput..

[186] Müller T., Dallos M., Lischka H. (1999). The Ethylene 1 1B1u V
State Revisited. J. Chem. Phys..

[187] Dallos M., Lischka H. (2004). A Systematic Theoretical
Investigation
of the Lowest Valence- and Rydberg-Excited Singlet States of Trans-Butadiene.
The Character of the 1 1Bu­(V) State Revisited. Theor. Chem. Acc..

[188] Chagas J. C. V., F dos Santos L. G., Nieman R., Aquino A. J. A., do Monte S. A., Plasser F., Szalay P. G., Lischka H., Machado F. B. C. (2025). Low-Lying Excited States of Linear All- *Trans* Polyenes: The σ–π Electron Correlation and the
Description of Ionic States. Phys. Chem. Chem.
Phys..

[189] Halkier A., Helgaker T., Jørgensen P., Klopper W., Koch H., Olsen J., Wilson A. K. (1998). Basis-Set
Convergence in Correlated Calculations on Ne, N2, and H2O. Chem. Phys. Lett..

[190] Kossoski F., Boggio-Pasqua M., Loos P.-F., Jacquemin D. (2024). Reference
Energies for Double Excitations: Improvement and Extension. J. Chem. Theory Comput.

[191] Schreiber M., Silva-Junior M. R., Sauer S. P. A., Thiel W. (2008). Benchmarks
for Electronically Excited States: CASPT2, CC2, CCSD, and CC3. J. Chem. Phys..

[192] Angeli C. (2009). On the Nature of the π →
Π* Ionic
Excited States: The V State of Ethene as a Prototype. J. Comput. Chem..

[193] Brédas J.-L., Norton J. E., Cornil J., Coropceanu V. (2009). Molecular
Understanding of Organic Solar Cells: The Challenges. Acc. Chem. Res..

[194] Low J., Yu J., Jaroniec M., Wageh S., Al-Ghamdi A. A. (2017). Heterojunction
Photocatalysts. Adv. Mater..

[195] Shao X., Aquino A. J. A., Otyepka M., Nachtigallová D., Lischka H. (2020). Tuning the UV Spectrum of PAHs by
Means of Different
N-Doping Types Taking Pyrene as Paradigmatic Example: Categorization:
Via Valence Bond Theory and High-Level Computational Approaches. Phys. Chem. Chem. Phys..

[196] Pinheiro M., Ferrão L. F. A., Bettanin F., Aquino A. J. A., Machado F. B. C., Lischka H. (2017). How to Efficiently
Tune the Biradicaloid Nature of Acenes by Chemical Doping with Boron
and Nitrogen. Phys. Chem. Chem. Phys..

[197] Pimentel J. V. M., Chagas J. C. V., Pinheiro M., Aquino A. J. A., Lischka H., Machado F. B. C. (2025). Thermally Activated Delayed Fluorescence
in B,N-Substituted Tetracene Derivatives: A Theoretical Pathway to
Enhanced OLED Materials. J. Phys. Chem. A.

[198] Zeng T., Mellerup S. K., Yang D., Wang X., Wang S., Stamplecoskie K. (2018). Identifying (BN) _2_ -Pyrenes
as a New Class of Singlet Fission Chromophores: Significance of Azaborine
Substitution. J. Phys. Chem. Lett..

[199] Pinheiro M., Machado F. B. C., Plasser F., Aquino A. J. A., Lischka H. (2020). A Systematic
Analysis of Excitonic Properties to Seek
Optimal Singlet Fission: The BN-Substitution Patterns in Tetracene. J. Mater. Chem. C Mater..

[200] Hachmann J., Dorando J. J., Avilés M., Chan G. K.-L. (2007). The Radical Character of the Acenes: A Density Matrix
Renormalization Group Study. J. Chem. Phys..

[201] Torres A.
E., Guadarrama P., Fomine S. (2014). Multiconfigurational
Character of the Ground States of Polycyclic Aromatic Hydrocarbons.
A Systematic Study. J. Mol. Model.

[202] dos Santos L. G. F., Chagas J. C. V., Ferrão L. F. A., Aquino A. J. A., Nieman R., Lischka H., Machado F. B. C. (2025). Tuning
Aromaticity, Stability and Radicaloid Character of Periacenes by Chemical
BN Doping. J. Comput. Chem..

[203] Plasser F., Lischka H. (2012). Analysis of Excitonic and Charge
Transfer Interactions from Quantum Chemical Calculations. J. Chem. Theory Comput..

[204] F dos Santos L. G., Chagas J. C. V., Nieman R., Aquino A. J. A., Machado F. B. C., Lischka H. (2025). Charge Transfer within
Excited States of Boron/Nitrogen Doped Polycyclic Aromatic Hydrocarbons. Phys. Chem. Chem. Phys..

[205] do Casal M. T., Toldo J. M., Barbatti M., Plasser F. (2023). Classification
of Doubly Excited Molecular Electronic States. Chem. Sci..

[206] Krauss M., Stevens W. J. (1984). Effective Potentials in Molecular
Quantum Chemistry. Annu. Rev. Phys. Chem..

[207] Dolg M., Stoll H. (1996). Chapter 152 Electronic Structure
Calculations for Molecules Containing Lanthanide Atoms. Handb. Phys. Chem. Rare Earths.

[208] Heß B. A., Marian C. M., Wahlgren U., Gropen O. (1996). A Mean-Field
Spin-Orbit Method Applicable to Correlated Wavefunctions. Chem. Phys. Lett..

[209] Zhang, Z. Quantum Algorithm for a Convergent Series of Approximations towards the Exact Solution of the Lowest Eigenstates of a Hamiltonian. 2020, arXiv:2009.03537. arXiv.org e-Printarchive. https://arxiv.org/abs/2009.03537.

[210] Veis L., Višňák J., Fleig T., Knecht S., Saue T., Visscher L., Pittner J. (2012). Relativistic Quantum Chemistry on Quantum Computers. Phys. Rev. A.

[211] Matsika S., Zhang Z., Brozell S. R., Blaudeau J.-P., Wang Q., Pitzer R. M. (2001). Electronic Structure and Spectra
of Actinyl Ions. J. Phys. Chem. A.

[212] Tyagi R., Zhang Z., Pitzer R. M. (2014). Electronic
Spectrum
of the UO and UO ^+^ Molecules. J.
Phys. Chem. A.

[213] Zhang Z., Pitzer R. M. (1999). Application of Relativistic
Quantum
Chemistry to the Electronic Energy Levels of the Uranyl Ion. J. Phys. Chem. A.

[214] Matsika S., Pitzer R. M. (2000). Electronic Spectrum
of the NpO_2_
^2+^ and NpO_2_
^+^ Ions. J. Phys. Chem. A.

[215] Yang T., Tyagi R., Zhang Z., Pitzer R. M. (2009). Configuration
Interaction Studies on the Electronic States of the CUO Molecule. Mol. Phys..

[216] Li J., Bursten B. E., Liang B., Andrews L. (2002). Noble Gas-Actinide
Compounds: Complexation of the CUO Molecule by Ar, Kr, and Xe Atoms
in Noble Gas Matrices. Science.

[217] Andrews L., Liang B., Li J., Bursten B. E. (2000). Ground-State
Reversal by Matrix Interaction: Electronic States and Vibrational
Frequencies of Cuo in Solid Argon and Neon. Angew. Chem., Int. Ed..

[218] Barbatti M., Lischka H. (2008). Nonadiabatic Deactivation
of 9H-Adenine:
A Comprehensive Picture Based on Mixed Quantum-Classical Dynamics. J. Am. Chem. Soc..

[219] Sellner B., Barbatti M., Müller T., Domcke W., Lischka H. (2013). Ultrafast Non-Adiabatic Dynamics
of Ethylene Including Rydberg States. Mol. Phys..

[220] Mukherjee S., Mattos R. S., Toldo J. M., Lischka H., Barbatti M. (2024). Prediction Challenge: Simulating Rydberg Photoexcited
Cyclobutanone with Surface Hopping Dynamics Based on Different Electronic
Structure Methods. J. Chem. Phys..

[221] Crespo-Otero R., Barbatti M. (2018). Recent Advances and
Perspectives
on Nonadiabatic Mixed Quantum–Classical Dynamics. Chem. Rev..

[222] Persico M., Granucci G. (2014). An Overview of Nonadiabatic
Dynamics
Simulations Methods, with Focus on the Direct Approach versus the
Fitting of Potential Energy Surfaces. Theor.
Chem. Acc..

[223] Pittner J., Lischka H., Barbatti M. (2009). Optimization of Mixed
Quantum-Classical Dynamics: Time-Derivative Coupling Terms and Selected
Couplings. Chem. Phys..

[224] Plasser F., Ruckenbauer M., Mai S., Oppel M., Marquetand P., González L. (2016). Efficient
and Flexible Computation
of Many-Electron Wave Function Overlaps. J.
Chem. Theory Comput..

[225] do Casal M. T., Toldo J. M., Pinheiro M., Barbatti M. (2021). Fewest Switches Surface Hopping with
Baeck-An Couplings. Open Res. Europe.

[226] Kossoski F., Barbatti M. (2020). Nonadiabatic Dynamics
in Multidimensional
Complex Potential Energy Surfaces. Chem. Sci..

[227] Martyka M., Zhang L., Ge F., Hou Y.-F., Jankowska J., Barbatti M., Dral P. O. (2025). Charting Electronic-State
Manifolds across Molecules with Multi-State Learning and Gap-Driven
Dynamics via Efficient and Robust Active Learning. npj Comput. Mater..

[228] Westermayr J., Gastegger M., Menger M. F. S. J., Mai S., González L., Marquetand P. (2019). Machine Learning
Enables Long Time Scale Molecular Photodynamics Simulations. Chem. Sci..

[229] Westermayr J., Marquetand P. (2021). Machine Learning
for Electronically
Excited States of Molecules. Chem. Rev..

[230] Dral P. O., Barbatti M. (2021). Molecular Excited States through
a Machine Learning Lens. Nat. Rev. Chem..

[231] Li J., Lopez S. A. (2022). A Look
Inside the Black Box of Machine Learning Photodynamics
Simulations. Acc. Chem. Res..

[232] Mai S., Marquetand P., González L. (2015). A General Method to Describe Intersystem
Crossing Dynamics in Trajectory Surface Hopping. Int. J. Quantum Chem..

[233] Mai S., Marquetand P., González L. (2018). Nonadiabatic Dynamics: The SHARC
Approach. WIREs Comput. Mol. Sci..

[234] Mai, S. ; Bachmair, B. ; Gagliardi, L. ; Gallmetzer, H. G. ; Grünewald, L. ; Hennefarth, M. R. ; Høyer, N. M. ; Korsaye, F. A. ; Mausenberger, S. ; Oppel, M. ; Piteša, T. ; Polonius, S. ; Sangiogo Gil, E. ; Shu, Y. ; Singer, N. K. ; Tiefenbacher, M. X. ; Truhlar, D. G. ; Vörös, D. ; Zhang, L. ; González, L. SHARC4.0: Surface Hopping Including Arbitrary Couplings – Program Package for Non-Adiabatic Dynamics 2025. https://sharc-md.org/. (accessed June 17, 2025).

[235] Richter M., Marquetand P., González-Vázquez J., Sola I., González L. (2011). SHARC: Ab Initio Molecular Dynamics
with Surface Hopping in the Adiabatic Representation Including Arbitrary
Couplings. J. Chem. Theory Comput..

[236] Westermayr J., Gastegger M., Marquetand P. (2020). Combining
SchNet and SHARC: The SchNarc Machine Learning Approach for Excited-State
Dynamics. J. Phys. Chem. Lett..

[237] Schütt K. T., Sauceda H. E., Kindermans P.-J., Tkatchenko A., Müller K.-R. (2018). SchNet – A Deep Learning Architecture
for Molecules and Materials. J. Chem. Phys..

[238] Schütt K. T., Kessel P., Gastegger M., Nicoli K. A., Tkatchenko A., Müller K.-R. (2019). SchNetPack:
A Deep Learning Toolbox For Atomistic Systems. J. Chem. Theory Comput.

[239] Mausenberger S., Müller C., Tkatchenko A., Marquetand P., González L., Westermayr J. (2024). S pai NN:
Equivariant Message Passing for Excited-State Nonadiabatic Molecular
Dynamics. Chem. Sci..

[240] Schütt, K. T. ; Unke, O. T. ; Gastegger, M. In Equivariant Message Passing for the Prediction of Tensorial Properties and Molecular Spectra, Proceedings of Machine Learning Research, PMLR, 2021.

[241] Dral P. O., Ge F., Xue B.-X., Hou Y.-F., Pinheiro M., Huang J., Barbatti M. (2021). MLatom 2: An Integrative
Platform for Atomistic Machine Learning. Top
Curr. Chem..

[242] Li J., Reiser P., Boswell B. R., Eberhard A., Burns N. Z., Friederich P., Lopez S. A. (2021). Automatic Discovery
of Photoisomerization
Mechanisms with Nanosecond Machine Learning Photodynamics Simulations. Chem. Sci..

[243] Westermayr J., Gastegger M., Vörös D., Panzenboeck L., Joerg F., González L., Marquetand P. (2022). Deep Learning Study of Tyrosine Reveals That Roaming
Can. Lead to Photodamage. Nat. Chem..

[244] Shu Y., Varga Z., Parameswaran A. M., Truhlar D. G. (2024). Fitting of Coupled
Potential Energy Surfaces via Discovery of Companion Matrices by Machine
Intelligence. J. Chem. Theory Comput..

[245] Gutleb, T. S. ; Barrett, R. ; Westermayr, J. ; Ortner, C. Parameterizing Intersecting Surfaces via Invariants. 2024, arXiv:2407.03731. arXiv.org e-Printarchive. https://arxiv.org/abs/2407.03731.

[246] Zechmann G., Barbatti M., Lischka H., Pittner J., Bonačić-Koutecký V. (2006). Multiple Pathways
in the Photodynamics
of a Polar π-Bond: A Case Study of Silaethylene. Chem. Phys. Lett..

[247] Lischka H., Dallos M., Szalay P. G., Yarkony D. R., Shepard R. (2004). Analytic Evaluation of Nonadiabatic
Coupling Terms
at the MR-CI Level. I. Formalism. J. Chem. Phys..

[248] Dallos M., Lischka H., Shepard R., Yarkony D. R., Szalay P. G. (2004). Analytic Evaluation of Nonadiabatic
Coupling Terms
at the MR-CI Level. II. Minima on the Crossing Seam: Formaldehyde
and the Photodimerization of Ethylene. J. Chem.
Phys..

[249] Barbatti M., Bondanza M., Crespo-Otero R., Demoulin B., Dral P. O., Granucci G., Kossoski F., Lischka H., Mennucci B., Mukherjee S., Pederzoli M., Persico M., Pinheiro M., Pittner J., Plasser F., Sangiogo
Gil E., Stojanovic L. (2022). Newton-X Platform: New Software Developments for Surface
Hopping and Nuclear Ensembles. J. Chem. Theory
Comput..

[250] Pederzoli M., Pittner J. (2017). A New Approach to Molecular Dynamics
with Non-Adiabatic and Spin-Orbit Effects with Applications to QM/MM
Simulations of Thiophene and Selenophene. J.
Chem. Phys..

[251] Wasif
Baig M., Pederzoli M., Kývala M., Cwiklik L., Pittner J. (2021). Theoretical
Investigation of the
Effect of Alkylation and Bromination on Intersystem Crossing in BODIPY-Based
Photosensitizers. J. Phys. Chem. B.

[252] Bhaskaran-Nair K., Demel O., Šmydke J., Pittner J. (2011). Multireference State-Specific
Mukherjee’s Coupled
Cluster Method with Noniterative Triexcitations Using Uncoupled Approximation. J. Chem. Phys..

[253] Lang J., Brabec J., Saitow M., Pittner J., Neese F., Demel O. (2019). Perturbative Triples Correction to
Domain-Based Local Pair Natural Orbital Variants of Mukherjee’s
State Specific Coupled Cluster Method. Phys.
Chem. Chem. Phys..

[254] Neese F. (2012). The ORCA Program System. WIREs Comput. Mol.
Sci..

